# ERK1/2-dependent gene expression in the bovine ovulating follicle

**DOI:** 10.1038/s41598-018-34015-4

**Published:** 2018-11-01

**Authors:** Yasmin Schuermann, Monique T. Rovani, Bernardo Gasperin, Rogério Ferreira, Juliana Ferst, Ejimedo Madogwe, Paulo B. Gonçalves, Vilceu Bordignon, Raj Duggavathi

**Affiliations:** 1 Department of Animal Science, McGill University, Sainte-Anne-de-Bellevue, QC H9X 3V9 Canada; 2 Laboratory of Biotechnology and Animal Reproduction, BioRep, Veterinary Hospital, Federal University of Santa Maria, Santa Maria, 97105-900 Brazil; 3 Laboratory of Animal Reproduction-ReproPEL, Federal University of Pelotas, 96010-610 Capão do Leão, Brazil; 4 Department of Animal Science, Santa Catarina State University, Santa Catarina, 88040-900 Brazil

## Abstract

Ovulation is triggered by gonadotropin surge-induced signaling cascades. To study the role of extracellular signal-regulated kinase 1/2 (ERK1/2) in bovine ovulation, we administered the pharmacological inhibitor, PD0325901, into the preovulatory dominant follicle by intrafollicular injection. Four of five cows treated with 50 µM PD0325901 failed to ovulate. To uncover the molecular basis of anovulation in ERK1/2-inhibited cows, we collected granulosa and theca cells from Vehicle and PD0325901 treated follicles. Next-generation sequencing of granulosa cell RNA revealed 285 differentially expressed genes between Vehicle and PD0325901-treated granulosa cells at 6 h post-GnRH. Multiple inflammation-related pathways were enriched among the differentially expressed genes. The ERK1/2 dependent LH-induced genes in granulosa cells included *EGR1, ADAMTS1, STAT3* and *TNFAIP6*. Surprisingly, PD0325901 treatment did not affect *STAR* expression in granulosa cells at 6 h post-GnRH. Granulosa cells had higher STAR protein and theca cells had higher levels of *STAR* mRNA in ERK1/2-inhibited follicles. Further, both granulosa and theca cells of ERK1/2-inhibited follicles had higher expression of *SLC16A1*, a monocarboxylate transporter, transporting substances including β-hydroxybutyrate across the plasma membrane. Taken together, ERK1/2 plays a significant role in mediating LH surge-induced gene expression in granulosa and theca cells of the ovulating follicle in cattle.

## Introduction

The process of ovulation is dependent upon the luteinizing hormone (LH) surge to elicit a plethora of signaling cascades required for the release of a fertilizable oocyte and formation of a corpus luteum (CL) needed for pregnancy^1,2^. Granulosa and theca cells of the ovulating follicle respond to the hormonal triggers, which in turn lead to their morphological and functional differentiation. The LH-dependent changes in the gene expression program is required for follicle rupture, cumulus cell expansion, oocyte maturation, and luteinization^3^.

The LH-surge has been shown to activate, in granulosa cells, signaling pathways including: phosphatidylinositide 3-kinase (PI3K/AKT), cAMP/Protein kinase A (PKA) and the extracellular signal-regulated kinase 1 and 2 (ERK1/2), also referred to as mitogen-activated protein kinase 3/1^4–9^. The ERK1/2 pathway is well-researched in the mouse model, whereby both pharmacological inhibition *in vivo*^10^ and *in vitro*^11^, and a granulosa cell conditional knockout mouse model^12^ have demonstrated the pivotal role of the ERK1/2 pathway in ovulatory processes. A number of pharmacological inhibitors (U0126, PD98059, and PD0325901) have been developed to impede ERK1/2 signaling, where PD0325901 was shown to have the least off-target effects and thus, making it the ideal candidate^13^. Nonetheless, all three inhibitors have been used to evaluate the role of ERK1/2 signaling in theca and/or granulosa cell cultures across multiple species including mouse^14^, bovine^7^, rat^15,16^, and human^16,17^.

Mouse studies have revealed that the absence of ERK1/2 signalling reduces the expression of LH-regulated genes including: the early growth response 1 (*Egr1)* transcription factor, as well as genes required for follicular rupture (a disintegrin and metalloproteinase with thrombospondin motifs 1 (*Adamts1)* and prostaglandin-endoperoxide synthase 2 (*Ptgs2)*), cumulus expansion (pentraxin 3*(Ptx3)* and TNF alpha induced protein 6 *(Tnfaip6)*), oocyte maturation (amphiregulin (*Areg)*) and luteinization markers (steroidogenic acute regulatory protein *(Star)* and cytochrome P450 family 11 subfamily a member 1 *(Cyp11a1)*)^10–12^.

Evaluation of ERK1/2 signaling in ovulatory-sized follicles in livestock species has been predominantly performed in cows, where granulosa and theca cells were pharmacologically treated *in vitro* by one of the aforementioned inhibitors. Furthermore, the experiments involved the collection of abattoir ovaries where theca and granulosa cells were isolated and treated with gonadotropins in the presence or absence of an ERK1/2 inhibitor for 15 minutes to 24 h^4,5,7,9,18–20^. For example, pharmacological inhibition of ERK1/2 signaling with U0126 was performed in cultured bovine granulosa cells from abattoir ovaries (follicles between 8 and 12 mm in diameter were selected). The following ovulatory genes were revealed to be down-regulated when cultured in the presence of forskolin (to induce the LH-surge) and U0126: *ADAMTS1, CXCR4, ADAM71, VNN2*, and *RGS2*^9,18–21^. While treatment of bovine theca cells with PD98059 *in vitro* resulted in increased *STAR* expression and thus, enhanced progesterone production^5^. Although these bovine studies have demonstrated a key role for ERK1/2 in regulation of select LH-regulated genes including *ADAMTS1* and *STAR* in granulosa and theca cells, the global impact of ERK1/2 signalling in bovine ovulation remains to be investigated.

Based on the aforementioned studies, we hypothesized that in the absence of ERK1/2 signaling LH-regulated genes downstream ERK1/2 would be differentially expressed leading to aberrant ovulation in cows. Therefore, our objective was to determine the role of ERK1/2 in bovine ovulation by means of developing a dynamic *in vivo* model, where follicular wave synchronized cows were subjected to intrafollicular injection of PD0325901 to abolish ERK1/2 signaling specifically in the ovulatory follicle. Moreover, by use of a novel approach of next generation sequencing, we performed RNA-sequencing to identify global changes in gene expression of granulosa cells of the ovulatory follicle exposed to PD0325901 and thus, gain a greater understanding of fertility in the bovine species.

## Results

### Inhibition of ERK1/2 signaling abolishes ovulation in cattle

**Figure 1:**
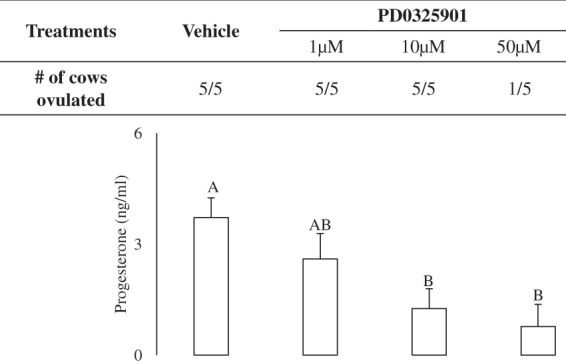
Effect of intrafollicular administration of the MEK inhibitor, PD0325901 on ovulation in cattle. All cows were subjected to follicular-wave synchronization and were treated with an ultrasound-guided intrafollicular administration of a Vehicle or different doses of PD0325901 30 minutes prior to intramuscular administration of GnRH. The number of cows ovulating in response to PD0325901 treatment are given in the table. Ultrasonography was used to identify the presence of a corpus luteum (CL). Progesterone levels in plasma samples collected five days after ovulation are presented in the graph. Bars with different letters are significantly different P < 0.05.

First, we tested the impact of inhibition of ERK1/2 signaling on ovulation in cows. The dominant follicle of the synchronized follicular wave was treated by intrafollicular injection with either a Vehicle or ERK1/2 signaling inhibitor, PD0325901 thirty minutes before GnRH treatment. Transrectal ultrasonography five days after the GnRH treatment revealed that all cows treated with Vehicle, 1 µM and 10 µM doses of PD0325901 successfully ovulated, while only one of five cows treated with 50 µM PD0325901 ovulated, however this cow had low levels of circulating progesterone, suggesting her CL was not functional (Fig. 1). Additionally, we measured plasma levels of progesterone on day 5 after GnRH treatment. Cows treated with 10 µM or 50 µM had significantly lower levels of progesterone compared to Vehicle treated counterparts (P < 0.05; Fig. 1). Therefore, we used 50 µM PD0325901 for all further experiments to investigate the molecular basis of anovulation in ERK1/2 inhibited ovulatory follicles in cattle.

**Figure 2:**
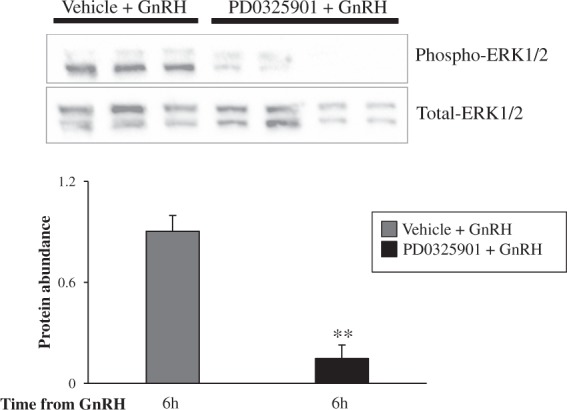
Inhibition of ERK1/2 activity in granulosa cells of ovulating follicles by an intrafollicular administration of PD0325901. Protein abundance of ERK1/2 phosphorylation in bovine granulosa cells collected from the dominant follicles of GnRH stimulated cows, which were challenged with a Vehicle control or 50 µM PD0325901. Quantification by densitometry are presented in the graph. The blot was cropped and it was first used to quantify the presence of Phospho-ERK1/2 and then stripped to quantify for the presence of Total-ERK1/2. ** denotes significant difference of Phospho-ERK1/2 normalized against Total-ERK1/2 between GnRH stimulated cows, which were challenged with a Vehicle control or 50 µM PD0325901, where P < 0.01.

Inhibition of ERK1/2 signaling in bovine granulosa cells by 50 µM PD0325901 was confirmed by protein analysis. At 6 h post-GnRH, there was lower abundance of phospho-ERK1/2 in granulosa cells of the ovulatory follicle treated with PD0325901 compared to those of follicles treated with Vehicle (P < 0.01; Fig. 2).

### Differentially expressed genes (DEGs) in granulosa cells

**Figure 3:**
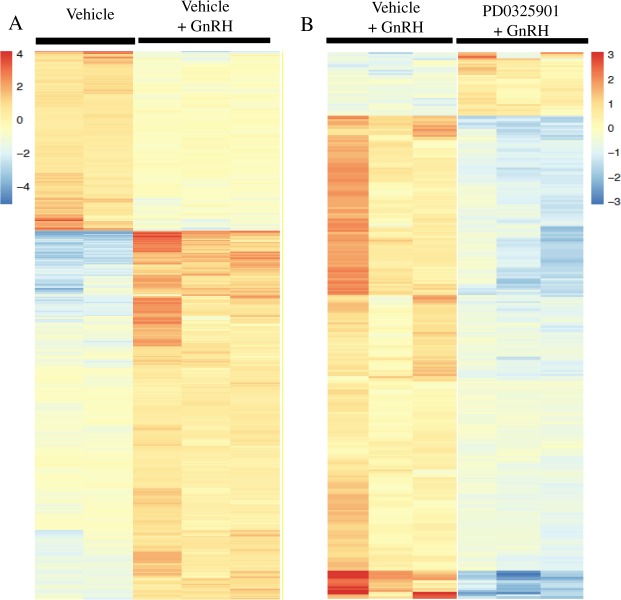
Heatmaps displaying differentially expressed genes (DEGs) in bovine granulosa cells for pairwise comparisons. (**A**) Heatmap of 2121 DEGs in granulosa cells collected from cows prior to (Vehicle; n = 2) or 6 h after GnRH stimulation (Vehicle + GnRH; n = 3). (**B**) Heatmap of 285 DEGs in granulosa cells collected at 6 h post-GnRH from cows that were pre-challenged, at 30 minutes prior to GnRH, with vehicle (Vehicle + GnRH; n = 3) or MEK inhibition (PD0325901 + GnRH; n = 3). The data from rlog transformed counts with high values and low values shown in red tones and blue tones, respectively. Significantly different genes were determined based on the statistical parameters: log2fold change < −1 and >1 with a q-value < 0.01.

**Figure 4:**
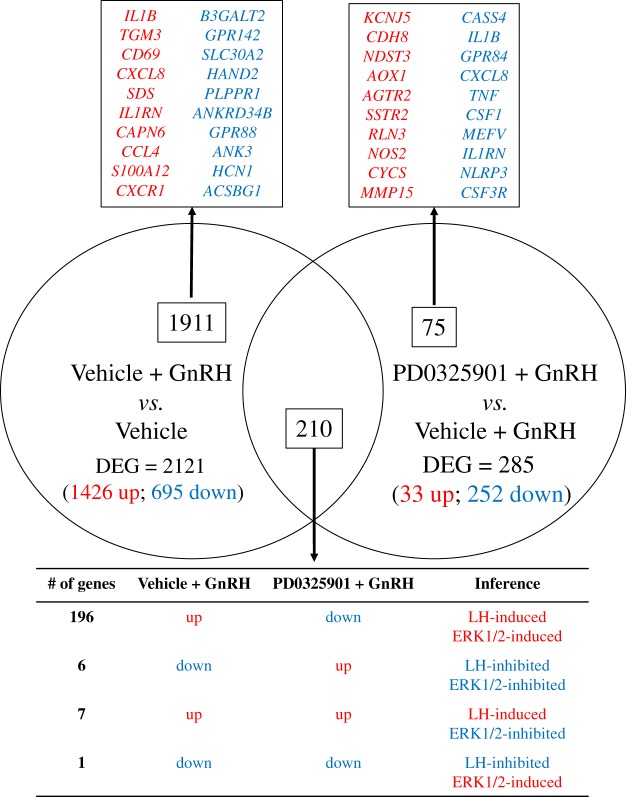
Venn diagram of differentially expressed genes (DEGs) for two pairwise comparisons: Vehicle + GnRH vs. Vehicle and PD0325901 + GnRH vs. Vehicle + GnRH. Top 10 up-regulated (red) and down-regulated (blue) DEGs are listed below for each specific comparison based on Log2FoldChange. The 210 DEGs that were common in both comparisons are represented with their respective direction of regulation in each comparison.

Having established that ERK1/2 activity is indispensable for ovulation, we next performed the second experiment to determine the ERK1/2 dependent gene expression in the bovine ovulatory follicle. We performed RNA-seq analysis on granulosa cells collected from the dominant follicles of the cows in three treatment groups: Vehicle, Vehicle + GnRH, and PD0325901 + GnRH. The reads aligned at over 86% to the bovine genome and the principle component analysis (PCA) showed that the transcriptomes of the three treatment groups separated out into three unique clusters (Supplementary Fig. 1 and Supplementary Table 1). We then performed pairwise differential genes expression analyses - first by comparing Vehicle + GnRH vs Vehicle to determine LH-induced genes and then by comparing PD0325901 + GnRH vs Vehicle + GnRH to determine ERK1/2 dependent genes. The pairwise differential analyses using the DESeq 2 R package showed that 2121 genes were differentially expressed between Vehicle + GnRH and Vehicle groups and 285 genes were differentially expressed between PD0325901 + GnRH and Vehicle + GnRH (>1 or <−1 log2fold change, q-value < 0.01; Fig. 3A,B). Of the 2121 LH-regulated DEGs from the first comparison, 1426 genes were upregulated and 695 genes were downregulated in Vehicle + GnRH granulosa cells relative to those of Vehicle group (a full list is available in Supplementary Table 2). The top ten upregulated genes were *IL1B*, *TGM3*, *CD69, CXCL8, SDS, IL1RN, CAPN6, CCL4, S100A12*, and *CXCR1* and the top ten downregulated genes included *B3GALT2, GPR142, SLC30A2, HAND2, PLPPR1, ANKRD34B, GPR88, ANK3, HCN1*, and *ACSBG1* (Fig. 4). Of the 285 ERK1/2-dependent DEGs from the second comparison, 33 genes were upregulated and 252 genes were downregulated in PD0325901 + GnRH granulosa cells relative to those of Vehicle + GnRH group (a full list is available in Supplementary Table 3). The top ten upregulated genes were *KCNJ5, CDH8, NDST3, AOX1, AGTR2, SSTR2, RLN3, NOS2, CYCS*, and *MMP15 CXCR1*, and the top ten downregulated genes included *CASS4*, *IL1B*, *GPR84*, *CXCL8*, *TNF*, *CSF1*, *MEFV*, *IL1RN*, *NLRP3*, and *CSF3R* (Fig. 4). In addition, there were 210 DEGs that were common between the two pairwise comparisons (Supplementary Table 4). Interestingly, the majority (196) of these 210 genes were upregulated in Vehicle + GnRH (LH-induced genes) and downregulated in PD0325901 + GnRH (ERK1/2-induced genes) granulosa cells. On the other hand, six genes were downregulated in Vehicle + GnRH (LH-inhibited genes) and upregulated in PD0325901 + GnRH (ERK1/2-inhibited genes) granulosa cells. Finally, only seven genes were upregulated in Vehicle + GnRH (LH-induced genes) followed by another upregulation in PD0325901 + GnRH (ERK1/2-inhibited genes) granulosa cells and only one gene was downregulated in Vehicle + GnRH (LH-inhibited gene) followed by a downregulation in PD0325901 + GnRH (ERK1/2-induced genes) granulosa cells.

### Gene ontology (GO) and pathway analysis of the DEGs

**Figure 5:**
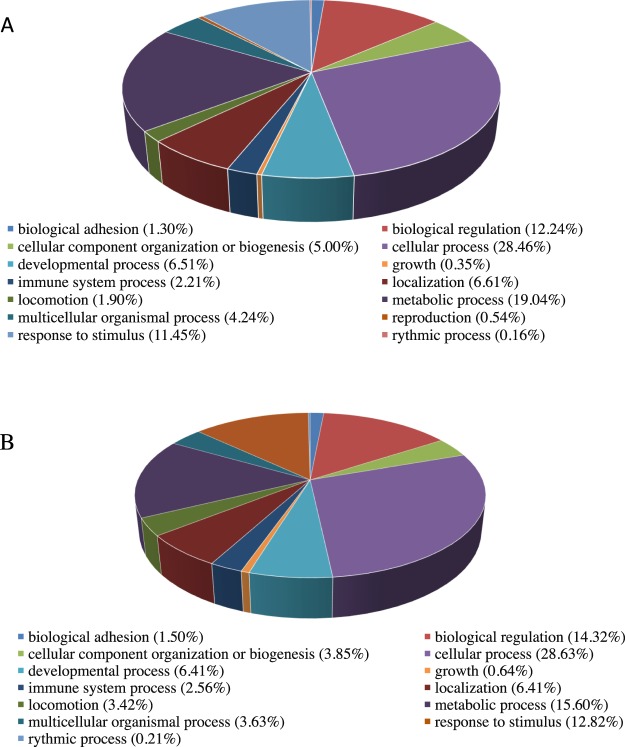
PANTHER GO-slim analysis. Pie-charts showing proportions of DEGs involved in biological processes of granulosa cells comparing Vehicle + GnRH to Vehicle (**A**) and PD032901 + GnRH to Vehicle + GnRH (**B**).

The DEGs were associated with 14 and 13 PANTHER classes (biological processes (BP)) for the comparison of Vehicle + GnRH vs Vehicle and PD0325901 + GnRH vs Vehicle + GnRH, respectively. Many of these BPs were overlapping between comparisons and include immune system processes and metabolic processes (Fig. 5A,B). To determine the pathways that were affected in the pairwise comparisons we used the DAVID tool to perform Kyoto Encyclopedia of Genes and Genomes (KEGG) pathway analysis. A table containing the list of top regulated pathways (P < 0.05) can be found in the Supplementary Table 5. A number of inflammatory pathways were altered between the two comparisons including amongst others the tumor necrosis factor (TNF) signaling pathways.

### Inhibition of ERK1/2 signaling does not inhibit global transcription in granulosa cells

**Figure 6:**
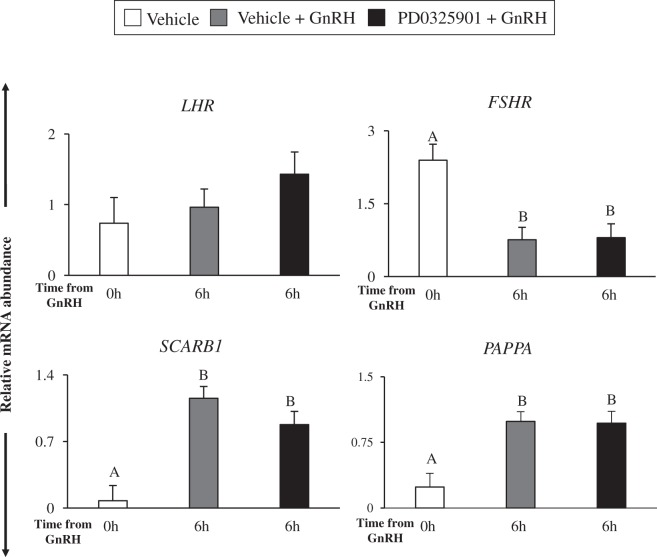
The MEK inhibitor PD0325901 treatment does not alter the global gene transcription system in bovine granulosa cells. Relative mRNA abundance of *LHR, FSHR, SCARB1*, and *PAPPA* in granulosa cells of cows in Vehicle, Vehicle + GnRH and PD0325901 + GnRH groups (N = 3–6/time-point). Cows were subject to follicular-wave synchronization and were challenged with Vehicle or PD0325901 30 minutes prior to intramuscular administration of GnRH. Granulosa cell were collected from groups of individual cows at 0 h and 6 h relative to GnRH stimulus. Transcript abundance of each gene was normalized to reference genes *ACTB, L19*, and *CYCLOPHILIN*. All data are expressed as a mean ± S.E.M, where different letters represent differences at p < 0.05 after a one-way ANOVA.

Although intrafollicular administration of PD0325901 led to abrogation of ovulation, not all genes were differentially expressed in granulosa cells of the treated follicles. Using RT-qPCR, we confirmed that the relative levels of *LHR* transcript were similar in granulosa cells of all three treatment groups (P > 0.05; Fig. 6). Relative mRNA abundance of *FSHR* was lower (P < 0.05; Fig. 6) in granulosa cells from the dominant follicles of both Vehicle and PD0325901 in GnRH-stimulated cows compared to those in cows that were not stimulated with GnRH. GnRH-induced increase in mRNA levels of *PAPPA* and *SCARB1* occurred in granulosa cells of both Vehicle and PD0325901 treated follicles (P < 0.05; Fig. 6). Similar differential expression trend for these genes was also observed in RNA-seq data (Table 1).

**Table 1:** Transcript abundance of 11 genes in bovine granulosa cells comparing Vehicle + GnRH to Vehicle and PD0325901 + GnRH to Vehicle + GnRH based on bioinformatics analysis. *q < 0.01.

ENSEMBL Gene ID	Gene Name	Vehicle + GnRH vs. Vehicle		PD0325901 + GnRH vs. Vehicle + GnRH	
		Log2FoldChange	q-value	Log2FoldChange	q-value
ENSBTAG00000016573	*LHR*	−0.60	0.57	0.68	0.34
ENSBTAG00000032424	*FSHR*	−1.17	0.15	0.27	0.99
ENSBTAG00000014269	*SCARB1*	2.36*	2.28E-05	0.06	0.99
ENSBTAG00000004010	*PAPPA*	2.73*	9.16E-4	−0.11	1.00
ENSBTAG00000007239	*TNFAIP6*	1.78	0.038	−1.51	0.05
ENSBTAG00000000706	*ADAMTS1*	3.84*	1.82E-12	−1.78*	3.6E-4
ENSBTAG00000005043	*TIMP1*	2.46*	9.9E-4	−2.48*	1.69E-05
ENSBTAG00000021523	*STAT3*	2.55*	1.50E-10	−1.19*	0.006
ENSBTAG00000010069	*EGR1*	2.67*	0.01	−2.73*	2.08E-04
ENSBTAG00000033345	*STAR*	2.87*	0.001	0.48	0.89
ENSBTAG00000015107	*SLC16A1*	−0.55	0.049	1.05*	7.50E-06

### Expression of LH-induced ovulatory genes in granulosa cells is reduced in the absence of ERK1/2 signaling

A multitude of genes are involved in follicle rupture, cumulus cell expansion, oocyte maturation, and luteinization, all of which are required for successful ovulation^3^. As previously mentioned, our bioinformatics analysis revealed that 196 genes were upregulated in Vehicle + GnRH (relative to Vehicle group) and downregulated in PD0325901 + GnRH (relative to Vehicle + GnRH group) granulosa cells (Fig. 4). From the list of 196 genes, we used RT-qPCR analysis to confirm the results of five genes (*EGR1, ADAMTS1, TNFAIP6, STAT3*, and *TIMP1*) known to be LH-induced based on studies performed in rodent^10,12,22^, primate^23^, and cattle^24,25^.

**Figure 7:**
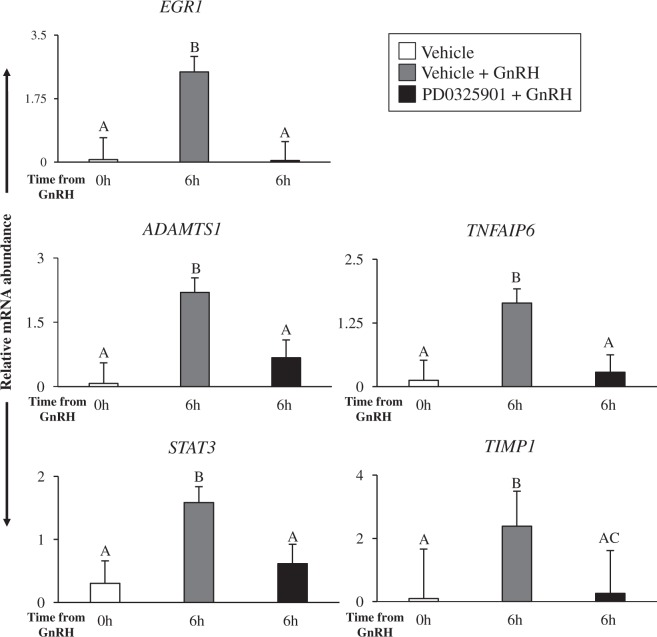
Intrafollicular injection of PD0325901 reduces the relative mRNA abundance of LH-induced ovulatory genes in granulosa cells. Relative mRNA abundance of *EGR1, ADAMTS1*, *TNFAIP6, STAT3*, and *TIMP1* in granulosa cells of cows in Vehicle, Vehicle + GnRH and PD0325901 + GnRH groups (N = 3–6/time-point). Cows were subject to follicular-wave synchronization and were challenged with Vehicle or PD0325901 30 minutes prior intramuscular administration to GnRH. Granulosa cell were collected from groups of individual cows at 0 h and 6 h relative to GnRH stimulus. Transcript abundance of each gene was normalized to reference genes *ACTB, L19*, and *CYCLOPHILIN*. All data are expressed as a mean ± S.E.M, where different letters (A&B) represent differences at p < 0.05 and C represents tendency for differences (P < 0.1) after a one-way ANOVA.

Granulosa cells in Vehicle + GnRH cows had higher levels of *EGR1* mRNA relative to Vehicle cows, whereas granulosa cells of PD0325901 + GnRH cows had lower *EGR1* mRNA abundance relative to Vehicle + GnRH cows (P < 0.05; Fig. 7). Similar mRNA abundance trend among cows of the three treatment groups was observed for *ADAMTS1, TNFAIP6*, *STAT3*, and *TIMP1* genes, wherein GnRH-induced increase in their mRNA levels was abolished by PD0325901 treatment (P < 0.05 & P < 0.1; Fig. 7). Similar differential expression trend for these genes was also observed in RNA-seq data (Table 1).

### Expression of LH-induced ovulatory genes in theca cells is altered in the absence of ERK1/2 signaling

**Figure 8:**
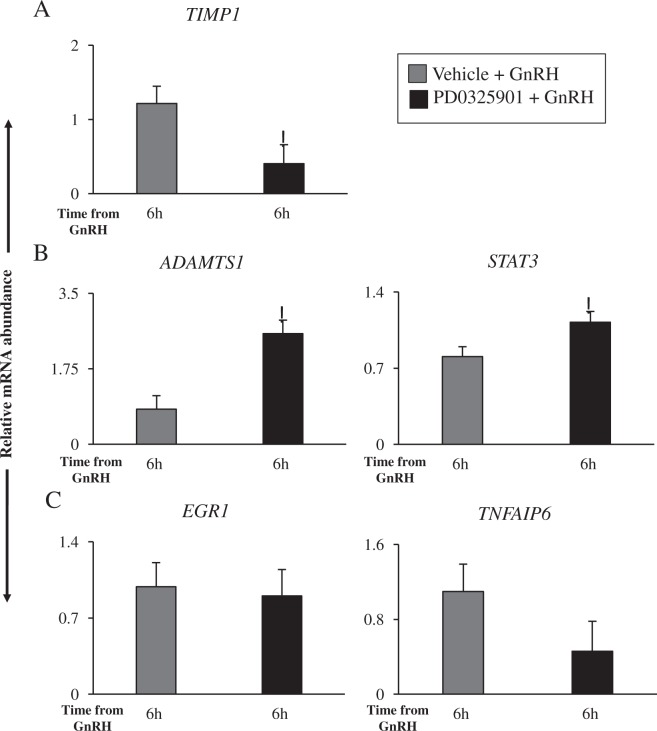
The impact of ERK1/2 pathway inhibition by PD0325901 on gene transcription in bovine theca cells. In the presence of PD0325901 following GnRH stimulation *TIMP1* is reduced in theca cells (**A**), while *ADAMTS1* and *STAT3* are induced (**B**). In bovine theca cells, *EGR1* and *TNFAIP6* remain unchanged between Vehicle + GnRH and PD0325901 + GnRH. Data were normalized to reference genes *ACTB, L19*, and *CYCLOPHILIN* (**C**). All data are expressed as a mean ± S.E.M, where * represents p < 0.05.

Successful ovulation and formation of a corpus luteum are dependent on functional and morphological changes in both granulosa and theca cells in response to the LH-surge^24,26^. Therefore, we sought to further analyze the impact of PD0325901 on the gene expression profile in bovine theca cells by comparing GnRH–stimulated theca cells in the absence or presence of PD0325901. Similar to bovine granulosa cells, relative mRNA abundance of *TIMP1* was significantly decreased in theca cells of the ovulatory follicles from the PD0325901 + GnRH group compared to the Vehicle + GnRH group (P < 0.05; Fig. 8A). Unlike in bovine granulosa cells, relative abundance of *ADAMTS1* and *STAT3* transcripts was higher in theca cells from the PD0325901 + GnRH follicles compared to the Vehicle + GnRH follicles (P < 0.05; Fig. 8B). Lastly, relative transcript levels of *EGR1* and *TNFAIP6* were unaltered between the two groups (P > 0.05; Fig. 8C).

### Inhibition of ERK1/2 signaling increases STAR abundance in granulosa and theca cells

**Figure 9:**
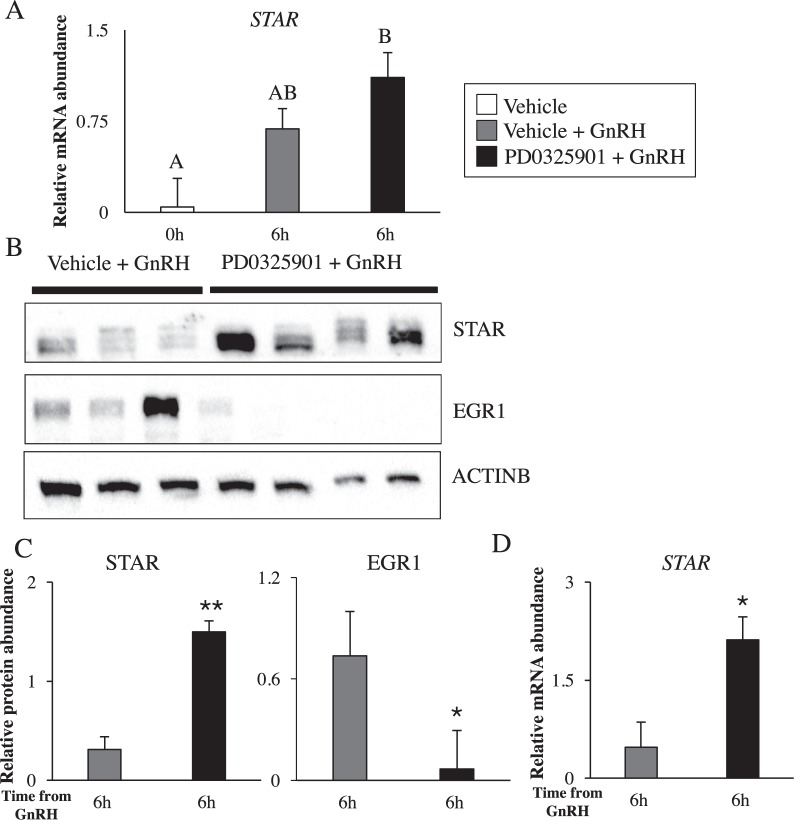
The impact of ERK1/2 pathway inhibition by PD0325901 on the transcription and translation of the steroidogenic enzyme STAR. (**A**) Relative mRNA abundance of *STAR* comparing Vehicle + GnRH and PD0325901 + GnRH. (**B**) STAR protein abundance by immunoblot in bovine granulosa cells subject to PD032590. EGR1 was used as a time-point control and ACTINB was used as a loading control. The blot was cut and cropped to analyze all three proteins by antibodies on the same blot. (**C**) Densitometry reveals quantitative abundance of STAR protein abundance in GnRH stimulated bovine granulosa cells in the presence of PD0325901. (**D**) RT-qPCR was performed on theca cells collected from cattle subject to GnRH treatment in the presence or absence of PD0325901 to establish the expression pattern of *STAR*. Transcript data were normalized to reference genes *ACTB, L19*, and *CYCLOPHILIN*. All data are expressed as a mean ± S.E.M, where letters (A&B) and * represents p < 0.05 and ** represents p < 0.01.

*STAR* is a luteinization marker expressed in bovine granulosa and theca cells^27,28^. Mouse studies have demonstrated that LH-induced *Star* expression in granulosa cells is dependent on ERK1/2 signaling^11,12^. As expected, our RNA-seq data showed an increase in *STAR* transcript abundance in Vehicle + GnRH vs Vehicle granulosa cells (Table 1). Surprisingly, there was no difference in the levels of *STAR* between PD0325901 + GnRH and Vehicle + GnRH granulosa cells (Table 1). Likewise, RT-qPCR analysis showed that there was no reduction in *STAR* mRNA levels in PD0325901 + GnRH granulosa cells when compared to Vehicle + GnRH granulosa cells (Fig. 9A). To confirm these transcript data, we used immunoblot analysis, which revealed PD0325901 + GnRH granulosa cells had higher levels of STAR protein than Vehicle + GnRH granulosa cells (P < 0.01; Fig. 9B,C). Using the same cell lysates, we observed lower abundance of EGR1 in PD0325901 + GnRH granulosa cells (P < 0.05; Fig. 9B,C). Similar to granulosa cells, relative mRNA abundance of *STAR* was higher in PD0325901 + GnRH theca cells (P < 0.05; Fig. 9D). Nonetheless, levels of progesterone in the follicular fluid collected at 6 h post-GnRH were similar between the two groups: 696.38 ± 193.82 ng/mL in Vehicle + GnRH and 827.80 ± 173.36 ng/mL in PD0325901 + GnRH (P > 0.05).

### ERK1/2 appears to regulate uptake of beta-hydroxoybutyric acid in granulosa and theca cells

**Figure 10:**
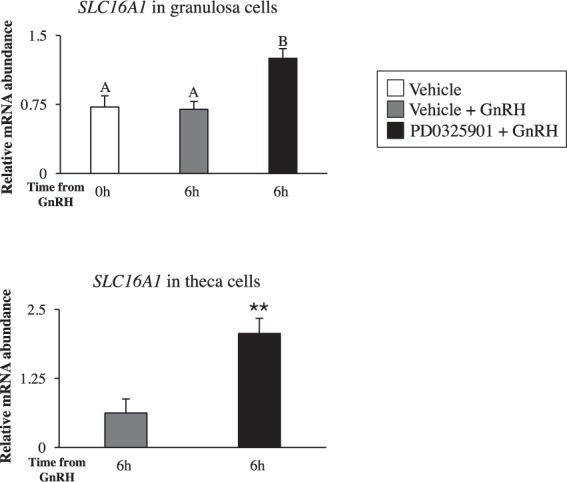
Intrafollicular administration of PD0325901 impact on SLC16A1. PD0325901 induces the relative mRNA abundance of solute carrier family 16 member 1 (*SLC16A1*), a suggested transporter of beta-hydroxybutyric acid, in both granulosa and theca cells. Transcript data were normalized to reference genes *ACTB, L19*, and *CYCLOPHILIN*. All data are expressed as a mean ± S.E.M, where different letters represents p < 0.05 and ** represents p < 0.01.

We found that in RNA-seq data solute carrier family 16 member A1 (*SLC16A1*; also known as monocarboxylate transporter-1) was one of the 33 upregulated genes in PD0325901 + GnRH granulosa cells (Table 1). Multiple studies have shown that cellular transport of β-hydroxybutyric acid (BHBA) occurs through SLC16A1^29,30^. We explored further the expression of *SLC16A1* in pre-ovulatory follicles as BHBA has been shown to increase proliferation and decrease steroidogenesis in cultured granulosa cells^31^. We confirmed upregulation of *SLC16A1* in PD0325901 + GnRH granulosa cells by RT-qPCR analysis (Fig. 10). Likewise, mRNA abundance of *SLC16A1* was significantly higher in theca cells collected from the dominant follicles of PD0325901 + GnRH compared to Vehicle + GnRH (P < 0.01; Fig. 10). No difference in the concentration levels of BHBA (0.45 ± 0.08 mmol/L Vehicle + GnRH and 0.34 ± 0.07 mmol/L PD0325901 + GnRH) and glucose (73.75 ± 17.4 mg/dL Vehicle + GnRH and 62.50 ± 17.4mgl/dL PD0325901 + GnRH) in follicular fluid was observed between the two groups of cows (P > 0.05).

## Discussion

In dairy cattle, anovulation on average persists in nearly 20% of dairy cows beyond the voluntary waiting period of breeding at 60 days in milk^32^. Detailed understanding of ovarian physiology is crucial to develop therapeutic and management strategies to enhance reproductive performance of lactating cows and to ensure sustainable dairy farming. Successful ovulation is dependent on the trigger elicited through the LH surge which in turn activates a cascade of signalling pathways, among others the ERK1/2 pathway^10^. This study is novel in its approach in using an *in vivo* model to investigate the role of ERK1/2 in bovine ovulation. We have shown for the first time in a monoovulator species, that the intrafollicular treatment with an inhibitor of ERK1/2 pathway (50 µM PD0325901) abrogates ovulation in cattle. Using next generation sequencing we were able to generate large data sets of transcript changes in bovine granulosa cells, which allowed us to confirm that anovulation is caused by downregulation of genes required for ovulation. To our knowledge, we are among few groups to analyze the transcriptome of bovine granulosa cells through RNA-sequencing^33,34^.

Our pairwise analysis of Vehicle + GnRH vs Vehicle cows revealed 2121 LH-regulated genes in granulosa cells of ovulating follicles. The immune system processes was one of the biological processes that was found to be regulated by the LH surge. Furthermore, the KEGG pathway analysis suggested that the JAK-STAT, TNF, and MAPK signaling pathways were among the pathways enriched among LH-regulated genes. All these biological processes and the pathways have been linked to inflammatory processes in multiple cell types^35,36^. Ovulation is an inflammation-like process and in line with this the genes involved in inflammation, such as *STAT3* and *TNFAIP6*^8,37–39^ were upregulated in response to the LH surge. Pairwise comparison of PD0325901 + GnRH vs Vehicle + GnRH cows showed that similar pathways were enriched among ERK1/2-dependent LH-regulated genes. These overall pairwise comparisons demonstrate that the ERK1/2 pathway regulates ovulation, similar to the mouse, by altering LH-regulated gene expression in granulosa cells of the ovulating follicle in cattle.

Some of the LH-induced gene were *SCARB1*, *PAPPA*, *EGR1*, *ADAMTS1*, *TNFAIP6, STAT3*, and *TIMP1*, which were also confirmed by RT-qPCR analyses. It has been previously shown that the expression of *SCARB1*, which is responsible for cellular uptake of high-density lipoproteins in steroidogenic tissues, increases as the bovine follicle size increases, using abattoir-collected ovaries^40^. Moreover, *Scarb1-null* mice are infertile despite normal ovarian morphology, estrus cycles, progesterone levels, and number of ovulated follicles^41^. However, the ovulated oocytes are dysfunctional and preimplantation embryos die shortly after mating, which may be caused due to abnormal lipoprotein metabolism^41,42^. Elsewhere, *PAPPA* expression has been shown in granulosa cells of the dominant and also in the ovulating follicle, suggesting that pathways downstream of LH may mediate *PAPPA* expression in cattle^43^. Additionally, *Pappa* knockout mice exhibit compromised fertility due to aberrant expression of steroidogenic genes, including *STAR*^44^. Our data along with these observations show that *SCARB1* and *PAPPA* may play critical roles in bovine ovulation. However, neither *SCARB1* nor *PAPPA* were downregulated in ERK1/2 inhibited granulosa cells. Similar lack of effect of ERK1/2 inhibition on *Scarb1* and *Pappa* expression was shown in a mouse study^10^. Therefore, these studies demonstrated that LH induction of *SCARB1* and *PAPPA* expression does not require ERK1/2 signaling and alternative signaling such as by the PKA pathway could be involved in their regulation^43^.

Comparing GnRH + PD0325901 to GnRH + Vehicle granulosa cells showed that many of the LH-induced genes, such as *EGR1, ADAMTS1, TNFAIP6, STAT3*, and *TIMP1*, were downregulated in ERK1/2 inhibited granulosa cells. Importantly, these genes were also downregulated in the absence of ERK1/2 signaling in granulosa cells of mice^10,12^. First, EGR1 plays an important role related to proliferation, differentiation, apoptosis, and gene regulation. Mice with *Egr1* deletion are sterile due to both ovarian and pituitary abnormalities^45^. In bovine ovulating follicles, *EGR1* is induced in granulosa cells at 6 h post-human chorionic gonadotropin (hCG) and suggested to play an additional role in prostaglandin biosynthesis pathway^46^. Our group previously reported that in mice, *Egr1* regulates *Ptgs2* expression by binding to its promoter^10^. Second, *Adamts1*-null mice experience subfertility^47^, which was attributed to aberrant extracellular matrix remodelling of the follicle wall required for ovulation^48^. In bovine granulosa cells *in vitro*, treatment with forskolin and ERK1/2 inhibitor (U0126) decreased *ADAMTS1* expression^21^. Third, *TNFAIP6* expression is induced between 4 h and 8 h post-hCG in rat granulosa cells^49^ and *Tnfaip6*-deficient mice are sterile due to abnormal remodelling of the cumulus extracellular matrix^50^. In cattle, *TNFAIP6* expression is up-regulated in the preovulatory follicle starting at 6 h post-hCG^8^. Moreover, this same group revealed by *in vitro* bovine granulosa cell culture that the PKA pathway was responsible for *TNFAIP6* promoter activity and transcription. However, they also confirmed that the activator protein-1 (AP-1) transcription factor was indispensable for *TNFAIP6* expression^8^. Previous work has suggested that ERK1/2 may phosphorylate the transcription factor AP-1^51^. In Supplementary Table 3, we show that FOS like 1, *AP-1* transcription factor subunit (ENSBTAG00000006194) is downregulated in GnRH + PD0325901 vs GnRH + Vehicle granulosa cells, providing a justification for the decrease in *TNFAIP6* expression. Fourth, TIMP1 is a key regulator of matrix metalloproteinases activity and production, and *Timp1*-null mice have shortened estrus period and altered serum levels of progesterone and estradiol^52^, yet surprisingly, these mice are fertile suggesting that there are redundant mechanisms that compensate for Timp1^53^. Lastly, STAT3 is a transcription factor involved in cytokine mediation and *Stat3*-null mice are embryonic lethal confirming the wide-spread expression of the gene^54^. Mice that are deficient of the progesterone receptor, which are infertile, experience a significant decrease in *Stat3* expression in ovaries^55^. Thus, STAT3, a regulator of inflammation, may play an important role in ovulation. On the contrary, there was an increase in *STAT3* expression in ERK1/2 inhibited theca cells. This difference in ERK1/2 regulation of STAT3 in response to ERK1/2 inhibition between granulosa and theca cells is not surprising. In fact, ERK1/2 appears to have different regulatory effect on *STAT3* expression and function in different cell types. Inhibition of ERK1/2 has been shown to increase *STAT3* expression in human hepatoma cell lines^56^ and cardiomyocytes^57^. In oral squamous cell carcinoma cells, it was shown that in the absence of ERK1/2 signaling, there was a decrease in phosphorylated -serine STAT3, but an increase in phosphorylated -tyrosine STAT3^58^.

The cow is an excellent model to study the impact of signaling pathways in theca cells, which is difficult in the murine model^59,60^. Christenson *et al*.^24^ have put together a list of differentially expressed genes comparing 0 h and 21 h GnRH-induced LH surge in both bovine granulosa and theca cells. This group identified *TIMP1* as being upregulated by LH in theca cells. Our data show that *TIMP1* expression in theca cells was downregulated as in granulosa cells of cows from the PD0325901 + GnRH vs Vehicle + GnRH. Similar to *STAT3*, *ADAMTS1* expression increased in ERK1/2 inhibited theca cells. Previous studies have shown that *ADAMTS1* has been detected in bovine theca cells and is increased by 24 h post-GnRH^61^, while *STAT3* has only yet been localized in theca cells of antral follicles in swine^62^. Also, expression of *EGR1* and *TNFAIP6* was not affected by the inhibitor treatment in theca cells. *EGR1* expression has been previously detected in bovine theca cells and induced by 6 h post-hCG^46,63^, while *TNFAIP6* mRNA abundance has been detected in both bovine and equine theca cells^64,65^. Theca cell transcriptome in bovine remains poorly studied, however these results contribute to the field of knowledge addressing signaling between follicular cell compartments.

The proper function of steroidogenic genes is crucial for ovulation and the formation of the corpus luteum. Of the multiple steroidogenic enzymes, STAR is the rate limiting enzyme and it facilitates the transport of intracellular cholesterol to the inner mitochondrial membrane for the onset of steroidogenesis and thus, progesterone synthesis. Mice deficient of *Star* have been shown to experience impaired ovulation^66,67^. Murine studies have shown that in the absence of ERK1/2 signaling there is a decrease in *Star* abundance and circulating progesterone concentrations^11,12^. On the contrary, immortalized steroidogenic granulosa cells and primary rat and human granulosa cells show an increase in *STAR* expression and progesterone production when cells were subjected to ERK1/2 inhibition^15,16^. Nonetheless, another study has shown that ERK1/2 inhibition attenuated *STAR* expression and progesterone synthesis in primary cell cultures of human granulosa cells^68^. In our *in vivo* bovine model, the LH-induced increase in *STAR* mRNA granulosa cells was not abolished by ERK1/2 inhibition. In fact, protein abundance was higher in both granulosa and theca cells of GnRH + PD0325901. In line with our data, treatment of bovine theca cells with PD98059 (ERK1/2 inhibitor) for 24 h increased *STAR* mRNA and protein abundance and progesterone production *in vitro*^5,7^.

RNA-sequencing of granulosa cells provided a diverse list of differentially expressed genes between different comparisons. One of the up-regulated genes in GnRH + PD0325901 granulosa cells was *SLC16A1*, which was further confirmed by RT-qPCR in both granulosa and theca cells. To our knowledge, this is the first study to report the presence of *SLC16A1* in both bovine granulosa and theca cells. Although limited data exists on the role of *SLC16A1*, it has been demonstrated that in the anterior pituitary cells of dairy cows cellular uptake of BHBA occurred through SLC16A1^29^. Moreover, elevated levels of BHBA reduced PKA activity, which subsequently prevented growth hormone and prolactin transcription and secretion from the pituitary cells^29^. Dairy cows during early lactation have elevated levels of circulating BHBA^69^. This ketone has been described as a source of energy used under conditions of starvation^70^. Previous research has shown that high levels of BHBA increased proliferation, but decreased estradiol and progesterone production in bovine granulosa cells^31^. As the preovulatory LH surge terminates proliferation and induces progesterone synthesis in granulosa cells, it is plausible that SLC16A1 may play a role in the process through regulation of BHBA uptake. Therefore, our data indicate that ERK1/2 mediates the LH-driven reduction in BHBA uptake to bring about luteinisation during ovulation.

In conclusion, ovulation in cattle, similar to the mouse, is dependent on proper ERK1/2 signaling, as a number of genes in granulosa cells required for different cascades in the ovulatory process are dysregulated in its absence. In contrast to mice, ERK1/2 inhibition increased STAR protein abundance in granulosa and theca cells. Similarly, genes such a *SLC16A1* were also elevated in granulosa cells lacking ERK1/2 signaling in both cell types. Moreover, some genes that were downregulated in response to ERK1/2 inhibition in granulosa cells were upregulated in theca cells. Taken together, ERK1/2 plays a significant role in transducing downstream signaling of LH surge during ovulation in cattle.

## Materials and Methods

### Cows and follicular synchronization

All beef cows in this study were used in accordance with procedures approved by the Ethics and Animal Welfare Committee of the Federal University of Santa Maria, Brazil. Follicular wave synchronization was performed by first (Day - 9) inserting an intravaginal device with 1 g progesterone (DIB; Intervet/Schering-Plough, Brazil), and intramuscular injection of 2 mg estradiol benzoate and 500 µg Sodium Cloprostenol. On day 0, the intravaginal device was removed and the ovaries were examined by transrectal ultrasonography using an 8 MHz linear-array transducer (AquilaVet® scanner, Pie Medical, the Netherlands), where cows having a follicle with a diameter >12 mm were subject to intrafollicular treatment described below.

### Experimental design

We conducted two experiments to establish the role of ERK1/2 activity on ovulation in cattle.

#### Experiment 1

We first tested whether or not inhibition of ERK1/2 activity in an ovulatory follicle would inhibit ovulation and formation of corpus luteum. As demonstrated by a study comparing three ERK1/2 pathway inhibitors^13^, unlike other inhibitors (U0126 and PD98059), PD0325901 does not appear to have off-target effects, thus making it an ideal ERK1/2 signaling inhibitor. Our group has previously shown that intraperitoneal administration of a single dose of PD0325901 in mice using a concentration of 25 µg/g body weight led to abrogated ovulation in mice without cytotoxicity^10^. Since intraperitoneal administration of the inhibitor in cows is not feasible, we sought to inhibit ERK1/2 activity by intrafollicular administration of PD0325901 (Selleckchem, Houston, Texas, USA).

**Figure 11:**
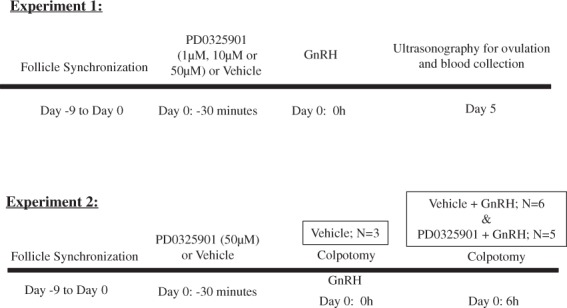
Experimental design for experiments 1 and 2. Timeline, treatment, and sample collection method for each experiment.

Experiment 1 was performed to determine the effective dose for inhibition of ovulation through dose trial. The dominant follicle of the synchronized follicular wave in separate groups of cows was treated on the morning of Day 0 with either a Vehicle control (Phosphate-buffered saline with DMSO; N = 5), or three different doses of PD0325901 (1 µM (N = 5), 10 µM (N = 5), or 50 µM (N = 5)). The diameter of the dominant follicle of each cow was measured by ultrasonography prior to injection. The accurate injection volume to obtain a final intrafollicular concentration of 1 µM, 10 µM, or 50 µM was determined based on the linear regression model equation for the follicular volume as previously described^71^: *V* = −*685.1* + *120.7D*, where *V* corresponds to the estimated follicular volume and *D* to the diameter of the follicle to be injected. The inhibitor was initially dissolved in dimethyl sulfoxide (DMSO) (Fisher Scientific, Saint-Laurent, QC, Canada). Thirty minutes following Vehicle or PD0325901 treatment (Day 0), cows were stimulated with an intramuscular dose of gonadotropin releasing hormone (GnRH) (100 µg gonadorelin acetate from Profertil®, Tortuga, Brazil) to induce the endogenous LH surge and thus, ovulation. It is well established that the peak of the endogenous LH surge occurs at 1 h after GnRH treatment^43^. Five days after GnRH treatment, a blood sample was collected from the coccygeal vein for plasma progesterone analysis and ultrasonography was performed to assess the presence of a corpus luteum. Progesterone concentrations were determined with an electrochemiluminescence immunoassay (Roche, Brazil) in a commercial lab (Laboratorio Pasin, Santa Maria, RS, Brazil). See Fig. 11 for experimental design.

#### Experiment 2

A second study was conducted to determine the molecular basis of ERK1/2 regulation of ovulation. We used the effective dose of 50 µM PD032901, which abrogated ovulation, to achieve ERK1/2 inhibition. Follicular wave synchronization and intrafollicular treatments were performed as described above. We collected ovaries from each cow at a specific developmental stage of the dominant follicle by colpotomy under caudal epidural anesthesia using 80 mg lidocaine chlorhydrate (4 ml 2% lidocaine) as previously described^72^. Ovary collection times included 0 h and 6 h relative to GnRH treatment representing before and after LH surge. The three groups of cattle involved in ovary collection included: 1) 0 h relative to GnRH treatment (Vehicle) (N = 3) – these cows were not treated with GnRH, but ovaries were collected at the expected time of GnRH treatment during the follicular wave synchronization protocol; 2) 6 h post-GnRH treatment with intrafollicular Vehicle (Vehicle + GnRH) (N = 6) and 3) 6 h post-GnRH treatment with intrafollicular PD0325901 (PD0325901 + GnRH) (N = 5). See Fig. 11 for experimental design. Upon collection, all ovaries were washed with a 0.9% saline solution followed by follicular puncture of the dominant follicle to collect samples for downstream analysis. Granulosa cells were collected through flushing of the follicle with phosphate buffered saline (PBS) for protein and gene expression analysis, followed by theca cell collection for gene expression analysis via dissection of the follicular wall. Follicular fluid was also collected. All samples, were stored in liquid nitrogen until return to the laboratory, where they were stored at −80 °C until further processing.

### RNA extraction and library preparation for RNA-sequencing of granulosa cells

To purify total RNA from granulosa cells we used the commercially available kit NucleoSpin® RNA Plus (Machery-Nagel, D-Mark Biosciences, ON, Canada) according to the manufacturer’s protocol. All samples, were quantified by absorbance at 260 nm using the NanoDrop 20000 spectrophotometer (Thermo Fischer Scientific Inc., Waltham, MA, USA). Total RNA was sent to the Functional Genomics Platform of McGill University and Génome Québec Innovation Centre, Canada for cDNA library preparation and sequencing with Illumina HiSeq 2500 PE 125, using 250 ng as input material. Only top-quality samples were prepared for sequencing, whereby a minimum RNA integrated Number (RIN) of 8.5 was accepted and library quality and quantity was confirmed using a bioanalyzer at Genome Quebec Research Center (N = 2-3 samples/treatment group). A total of 8 samples were selected based on quality assessment: Vehicle (N = 2), Vehicle + 6hGnRH (N = 3) and PD0325901 + 6hGnRH (N = 3) and they were run together on one lane. The RNA-seq data used in this study are available from GEO data repository (GSE121588). Supplementary Table 1 and Supplementary Fig. 1 provide details on RNA-seq quality.

### Bioinformatics analysis: read cleaning and genome alignment

FastQC v0.11.5 was used to visualize the quality of the sequence reads^73^. These reads were cleaned using the software Trimmomatic v0.36, where illumina adapters and reads with average PHRED scores below 20 and lengths below 63 base pairs (bp) were removed^74^. The FastQC software revealed clean per tile sequence and per base sequence quality. Quality of a representative sample can be seen in Supplementary Fig. 1A,B. The index of the bovine genome, *Bos taurus* genome B.tau 4.6.1, was built using Bowtie v2.2.1.0 and the cleaned reads were aligned to the genome using TopHat v2.0.11 with the following modifications to the default settings: final read alignments having more than 3 mismatches were removed, 100 base pairs (bp) as the expected inner distance between mate pairs, 50 bp as the standard deviation for the distribution on inner distances between mate pairs, 5 as an anchor length, 1 as the maximum number of mismatches in the anchor, 50 as the minimum intron length, 2 as the number of threads to align reads, 3 as the segment alignment mismatches, 20 as a minimum intron length found during split-segment search^75^. Finally, the number of reads mapped to each gene were counted using HTSeq v0.6.1^76^.

### Differentially expressed genes (DEGs)

R studio v3.3.0 was used for statistical analysis of granulosa cell sequencing data, where R packages were used to determine differently expressed genes (DEGs) between treatment groups. The counts were normalized using the estimateSizeFactors and variance were stabilized using varianceStabilizingTransformaton commands in the statistical package DESeq. 2 v1.12.3^77^. A principle component analysis (PCA) was used to detect treatment clustering (Supplementary Fig. 1C). DESeq 2 was used to build a list of DEGs by a negative binomial generalized linear model with the following criteria: Log2Fold Change of >1 or <−1 and a Benjamini-Hochberg false discover rate (FDR)/q-value < 0.01. Heatmaps were constructed using the R package Pheatmap v1.0.8 and a Venn diagram for pairwise comparisons of DEGs within treatments was assembled with the Bioinformatics & Evolutionary Genomics website (http://bioinformatics.psb.ugent.be/webtools/Venn/).

### Gene ontology and pathway enrichment

The identified DEGs were analyzed in the PANTHER classification system (13.1) to determine enriched biological systems. For functional annotation clustering and enrichment score representation of Gene Ontology (GO), we used the Data-base for Annotation, Visualization and Integrated Discovery (DAVID) version 6.8 to generate the Kyoto Encyclopedia of Genes and Genomes (KEGG) pathways.

### RT-qPCR of granulosa and theca cells

Total RNA from granulosa cells was extracted as previously described. Total RNA from theca cells was purified using TRizol® (Thermo Fisher Scientific [Life Technologies, Inc.], Burlington, ON, Canada) according to the manufacturer’s instructions. All samples, were quantified by absorbance at 260 nm using the NanoDrop 20000 spectrophotometer (Thermo Fischer Scientific Inc., Waltham, MA, USA). Complementary DNA was synthesized from 250 ng of total RNA using the iScript cDNA Synthesis kit (Bio-Rad, Mississauga, Canada) using the following temperature program: 25 °C for 5 min (Priming), 46 °C for 20 min (Reverse transcription) and 95 °C for 1 min (Reverse transcription inactivation).

A larger number of granulosa cells samples was used to perform RT-qPCR analysis compared to RNA-seq: Vehicle (N = 3), Vehicle + GnRH (N = 6) and PD0325901 + GnRH (N = 4). Gene expression of *CYP17A1* was used as a marker for theca cell contamination and thus, affected granulosa cell samples were removed from the analysis (one sample from PD0325901 + GnRH group was removed). The mRNA abundance of theca cells was compared between two treatment groups, Vehicle + GnRH (N = 6) and PD0325901 + GnRH (N = 5). All RT-qPCR assays were performed using previously described protocols^78^. Relative transcript abundance for each gene of interest was calculated by dividing their respective starting quantity (SQ) values by the mean SQ values of three reference genes (*ACTB, L19*, and *CYCLOPHILIN*). The primer sequences of transcripts measured in this study can be found in Supplementary Table 6, where primer design was performed using the NCBI Primer-BLAST. If variants of a gene are present, the primers were designed to include all variants.

### Protein isolation and immunoblot

Protein from granulosa cells was isolated using the AllPrep® DNA/RNA/Protein kit (Qiagen, Mississauga, ON, Canada), followed by a dilution in Laemmli buffer (Bio-Rad Laboratories, Mississauga, ON, Canada) and boiled at 95 °C for 5 min. Protein extracts were resolved by polyacrylamide electrophoresis (10% gel) and transferred to nitrocellulose membranes. Blocking after the transfer was performed for 1.5 h using 5% milk in Tris-buffered saline with 0.1% Tween-20 (TBS-T). All membrane fractions were incubated with their respective primary antibodies (STAR, Phospho-ERK1/2 and EGR1) overnight at 4 °C. Next, membranes were washed with TBS-T (4 × 7 min) followed by incubation with secondary antibody for 1.5 hours at room temperature. The Immun-Star Western Chemi luminescent Kit (Bio-Rad) and Chemidoc Analyzer were used to detect immunoblotted proteins. The membrane was stripped twice for detection of Total-ERK1/2 and β-actin using a stripping buffer (10% SDS, 0.5 M Tris-HCl, milliQ grade water and 2-mercaptoehtanol). We established ERK1/2 activity by determining the abundance of phosphorylated isoform of ERK1/2 relative to its total isoform. All antibodies used in the experiment have been validated in multiple species. The references for the studies that used these antibodies in the bovine ovary are given in Supplementary Table 7 with additional information pertaining to the required concentration and the purchasing information for each antibody. Image Lab Software from Bio-Rad was used to quantify protein abundance. The images of the immunoblots in Figs 2 and 9 were cropped and thus, uncropped images are available in Supplementary Fig. 2.

### Follicular fluid analysis

Progesterone concentrations were determined with an electrochemiluminescence immunoassay (Roche, Brazil) in a commercial lab^79^. Glucose and beta-hydroxybutyric acid (BHBA)/ketone levels were measured with the FreeStyle Optium Neo Blood Glucose and Ketone Monitoring System from Abbott (available from local pharmacies; Abbott Laboratories, Limited).

### Statistical analysis for RT-PCR, immunoblot, and follicular fluid analysis

Analysis of data was performed using SAS University Edition (SAS Institute). Data that did not follow a normal distribution (Shapiro-Wilk test) were log-transformed. Differences between means were tested with the Tukey-Kramer honestly significant difference test or by Student’s t-test. Data are presented as the mean ± SEM and significant differences were designated by P < 0.05 and a tendency was marked by P < 0.1.

## Electronic supplementary material

Supplementary Information
