# Supplementary material for: ERK1/2-dependent gene expression in the bovine ovulating follicle
Source: Sci Rep. 2018 Nov 1;8:16170. doi: 10.1038/s41598-018-34015-4 (PMC6212447; doi:10.1038/s41598-018-34015-4)
Supplement: Supplementary file 1 — Supplementary Information [file 41598_2018_34015_MOESM1_ESM.pdf]

Supplementary Information for the following manuscript (SREP-18-24310A):

ERK1/2-dependent gene expression in the bovine ovulating follicle

Yasmin Schuermann[1], Monique T. Rovani[2], Bernardo Gasperin[3], Rogério Ferreira[4], Juliana, Ferst[2], Ejimedo Madogwe[1], Paulo B. Gonçalves[2], Vilceu Bordignon[1], and Raj Duggavathi[1*]

[1]Department of Animal Science, McGill University, Sainte-Anne-de-Bellevue, QC H9X 3V9, Canada

[2]Laboratory of Biotechnology and Animal Reproduction, BioRep, Veterinary Hospital, Federal University of Santa Maria, Santa Maria, 97105-900, Brazil,

[3]Laboratory of Animal Reproduction-ReproPEL, Federal University of Pelotas, 96010-610, Capão do Leão, Brazil,

[4]Department of Animal Science, Santa Catarina State University, Santa Catarina, 88040-900, Brazil

***Corresponding author:**

Raj Duggavathi,

raj.duggavathi@mcgill.ca,

McGill University, Animal Science, 21111, Lakeshore road, Ste-Anne-de-Bellevue, QC, CAN H9X3V9

Tel: 514-398-7793

Supplementary Figure 1: Quality assessment of RNA-seq on bovine granulosa cells. A. Bioanalysis report revealing peak of a representative sample. B. Per tile sequence quality and per base sequence quality using the FastQC software of a representative sample. C. Principle component analysis of the treatment groups following alignment.

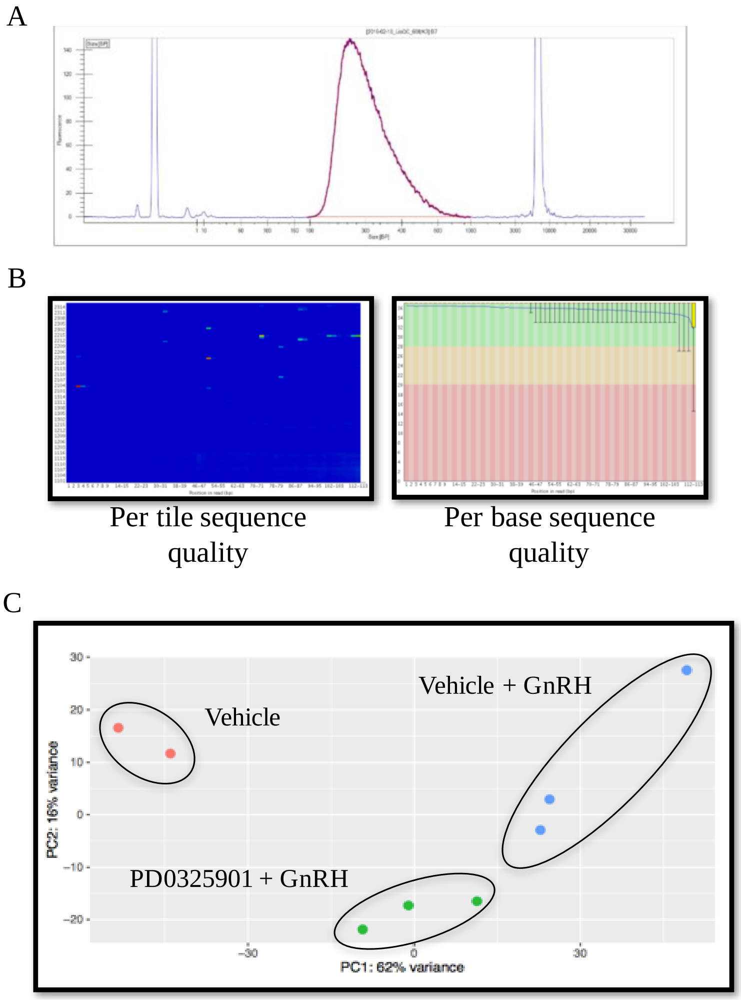

Supplementary Table 1: RNA-seq quality and alignment assessment of bovine granulosa cells

RNA-Seq Quality and Alignment			
Groups	0h Vehicle	6h Vehicle	6h PD0325901
# of Samples	2	3	3
RNA Integrated Number (Bioanalysis)	>9.2	>9.1	>8.5
260/280 (Nanodrop Quantification)	1.8	2.0-2.1	2.0-2.1
Avg. # of Raw Reads	31 580 928	34 823 800	32 781 670
Avg. # of Cleaned Reads/Sample/Group	23 577 628	26 879 332	25 478 223
Avg. Alignment/Group	87.35%	87.10%	86.93%

Supplementary Table 2: List of 2121 LH-regulated DEGs

	Vehicle + GnRH vs Vehicle					
ENSEMBL ID	baseMean	log2FoldChange	lfcSE	stat	pvalue	padj
ENSBTAG00000001321	8261.7540	12.0968	0.7821	15.4678	5.73E-54	7.72E-51
ENSBTAG00000001785	3696.4557	11.1564	0.7803	14.2968	2.29E-46	1.93E-43
ENSBTAG00000002135	2593.0253	10.9227	0.8784	12.4344	1.70E-35	6.37E-33
ENSBTAG00000047904	1431.0922	10.5501	0.8595	12.2752	1.23E-34	4.37E-32
ENSBTAG00000019716	2339.4418	10.4515	0.8099	12.9050	4.22E-38	1.72E-35
ENSBTAG00000031814	5290.3366	10.3945	0.7666	13.5585	7.05E-42	3.66E-39
ENSBTAG00000019665	13716.7991	10.2751	0.7304	14.0677	6.00E-45	4.49E-42
ENSBTAG00000000828	9861.1147	9.9304	0.6556	15.1472	7.91E-52	8.88E-49
ENSBTAG00000025257	935.9293	9.7840	0.9025	10.8413	2.19E-27	5.19E-25
ENSBTAG00000012638	2752.3662	9.7115	0.8633	11.2487	2.35E-29	5.98E-27
ENSBTAG00000026753	910.5394	9.5577	0.9241	10.3422	4.54E-25	9.14E-23
ENSBTAG00000013334	1444.2931	9.3142	0.8107	11.4891	1.50E-30	4.12E-28
ENSBTAG00000037558	1112.0383	8.7569	0.9449	9.2675	1.91E-20	2.31E-18
ENSBTAG00000015061	439.2316	8.7541	0.9447	9.2669	1.92E-20	2.31E-18
ENSBTAG00000019123	2648.8416	8.7258	0.7078	12.3279	6.41E-35	2.34E-32
ENSBTAG00000012640	1103.8561	8.6338	0.8867	9.7365	2.11E-22	3.46E-20
ENSBTAG00000020713	587.4162	8.6275	0.7312	11.7997	3.92E-32	1.17E-29
ENSBTAG00000015592	818.7520	8.6058	0.9271	9.2821	1.66E-20	2.07E-18
ENSBTAG00000038042	431.1692	8.5962	0.9602	8.9524	3.48E-19	3.61E-17
ENSBTAG00000015865	495.4450	8.3334	0.8907	9.3565	8.24E-21	1.13E-18
ENSBTAG00000006505	1588.9178	8.2961	0.8419	9.8543	6.57E-23	1.17E-20
ENSBTAG00000007901	463.4886	8.2339	0.8847	9.3072	1.31E-20	1.67E-18
ENSBTAG00000034918	294.4042	8.0883	0.9822	8.2350	1.80E-16	1.33E-14
ENSBTAG00000027513	577.1408	8.0270	0.9594	8.3669	5.92E-17	4.78E-15
ENSBTAG00000019800	261.7667	7.9654	0.8174	9.7448	1.94E-22	3.23E-20
ENSBTAG00000016771	5946.4817	7.9561	0.5101	15.5962	7.72E-55	1.30E-51
ENSBTAG00000006354	348.1341	7.9526	1.0083	7.8874	3.09E-15	2.00E-13
ENSBTAG00000039046	305.4257	7.8987	1.0044	7.8639	3.72E-15	2.39E-13
ENSBTAG00000014555	167.1680	7.8684	0.9540	8.2476	1.62E-16	1.22E-14
ENSBTAG00000027246	665.5860	7.7821	0.8559	9.0920	9.72E-20	1.07E-17
ENSBTAG00000004714	321.8837	7.7601	1.0210	7.6008	2.94E-14	1.62E-12
ENSBTAG00000020580	433.3525	7.7515	0.9600	8.0744	6.78E-16	4.64E-14
ENSBTAG00000007052	143.5162	7.7433	0.9536	8.1200	4.66E-16	3.29E-14
ENSBTAG00000023198	199.8824	7.7203	0.9896	7.8017	6.11E-15	3.71E-13
ENSBTAG00000048135	262.4241	7.7040	1.0128	7.6067	2.81E-14	1.55E-12
ENSBTAG00000013451	305.3331	7.6876	0.7630	10.0754	7.09E-24	1.37E-21
ENSBTAG00000022396	499.6435	7.6558	0.9196	8.3253	8.41E-17	6.52E-15
ENSBTAG00000018424	233.9842	7.6475	0.8670	8.8205	1.14E-18	1.09E-16
ENSBTAG00000003326	649.3799	7.5548	0.8589	8.7963	1.41E-18	1.34E-16

ENSBTAG00000002623	1362.8380	7.5297	0.7740	9.7279	2.29E-22	3.72E-20
ENSBTAG00000013210	22410.2649	7.4578	0.4618	16.1478	1.18E-58	2.27E-55
ENSBTAG00000021717	295.7356	7.4516	0.6796	10.9648	5.64E-28	1.36E-25
ENSBTAG00000013798	333.5431	7.4216	0.7436	9.9805	1.86E-23	3.38E-21
ENSBTAG00000025471	247.9527	7.4121	0.9136	8.1130	4.94E-16	3.45E-14
ENSBTAG00000019712	255.6339	7.3723	0.8099	9.1025	8.83E-20	9.84E-18
ENSBTAG00000018125	241.1259	7.3459	0.7872	9.3318	1.04E-20	1.39E-18
ENSBTAG00000007392	364.7516	7.3231	0.8064	9.0818	1.07E-19	1.17E-17
ENSBTAG00000031160	772.0731	7.3212	0.8013	9.1366	6.44E-20	7.30E-18
ENSBTAG00000004716	153.6408	7.3018	1.0141	7.1999	6.03E-13	2.82E-11
ENSBTAG00000006949	234.1523	7.2971	0.9234	7.9027	2.73E-15	1.80E-13
ENSBTAG00000000507	13486.2815	7.2711	0.4854	14.9808	9.80E-51	1.02E-47
ENSBTAG00000002340	155.9542	7.2697	1.0188	7.1353	9.66E-13	4.37E-11
ENSBTAG00000011515	313.3737	7.2380	0.7774	9.3102	1.28E-20	1.64E-18
ENSBTAG00000046158	894.0882	7.2365	0.6577	11.0028	3.71E-28	9.09E-26
ENSBTAG00000005965	886.4357	7.2350	0.6164	11.7374	8.20E-32	2.40E-29
ENSBTAG00000004261	170.2148	7.2240	1.0302	7.0124	2.34E-12	9.96E-11
ENSBTAG00000008545	1855.1721	7.2092	0.5627	12.8114	1.42E-37	5.61E-35
ENSBTAG00000001273	337.2373	7.1719	0.8459	8.4785	2.28E-17	1.90E-15
ENSBTAG00000021714	3092.7993	7.0675	0.3802	18.5892	3.93E-77	1.76E-73
ENSBTAG00000020602	289.3507	7.0652	0.8002	8.8291	1.06E-18	1.02E-16
ENSBTAG00000001414	225.7132	7.0284	0.7256	9.6864	3.45E-22	5.53E-20
ENSBTAG00000047700	169.1094	7.0046	0.9155	7.6511	1.99E-14	1.12E-12
ENSBTAG00000012894	291.7767	6.9739	0.8535	8.1706	3.07E-16	2.20E-14
ENSBTAG00000001864	996.0537	6.9088	0.5068	13.6308	2.63E-42	1.48E-39
ENSBTAG00000007191	279.2906	6.9002	0.7340	9.4004	5.43E-21	7.79E-19
ENSBTAG00000048049	308.4976	6.8803	0.9563	7.1947	6.26E-13	2.91E-11
ENSBTAG00000001198	98.1866	6.8707	1.0257	6.6987	2.10E-11	7.86E-10
ENSBTAG00000000671	686.0784	6.8491	0.8780	7.8008	6.15E-15	3.72E-13
ENSBTAG00000047379	104.1963	6.8126	1.0372	6.5682	5.09E-11	1.79E-09
ENSBTAG00000014368	168.5765	6.7992	0.9656	7.0413	1.91E-12	8.29E-11
ENSBTAG00000039037	148.5453	6.7946	1.0631	6.3912	1.65E-10	5.34E-09
ENSBTAG00000021326	198.5533	6.7724	1.0797	6.2724	3.56E-10	1.08E-08
ENSBTAG00000046257	379.3798	6.7459	0.8114	8.3138	9.27E-17	7.14E-15
ENSBTAG00000006343	95.3510	6.7426	1.0380	6.4956	8.27E-11	2.79E-09
ENSBTAG00000009768	428.0743	6.7269	0.6021	11.1717	5.61E-29	1.40E-26
ENSBTAG00000016424	145.9206	6.7050	0.9567	7.0088	2.40E-12	1.02E-10
ENSBTAG00000018223	112.6090	6.7027	0.9003	7.4450	9.69E-14	5.02E-12
ENSBTAG00000011982	174.5282	6.7023	0.8733	7.6744	1.66E-14	9.41E-13
ENSBTAG00000015707	76.3656	6.6953	1.0245	6.5355	6.34E-11	2.18E-09
ENSBTAG00000002937	117.0325	6.6845	1.0590	6.3118	2.76E-10	8.65E-09
ENSBTAG00000025250	553.0634	6.6539	0.7125	9.3386	9.76E-21	1.32E-18
ENSBTAG00000000735	476.8205	6.6044	0.7824	8.4407	3.16E-17	2.59E-15

ENSBTAG00000046266	105.2631	6.6026	1.0603	6.2271	4.75E-10	1.40E-08
ENSBTAG00000047268	78.3540	6.6000	1.0383	6.3566	2.06E-10	6.59E-09
ENSBTAG00000030940	315.7470	6.5905	0.8579	7.6823	1.56E-14	8.97E-13
ENSBTAG00000019892	70.4859	6.5880	1.0303	6.3943	1.61E-10	5.25E-09
ENSBTAG00000017116	1318.0075	6.5871	0.7642	8.6200	6.70E-18	5.98E-16
ENSBTAG00000014402	476.5076	6.5661	0.6691	9.8126	9.94E-23	1.70E-20
ENSBTAG00000010464	92.2591	6.5621	0.8718	7.5267	5.20E-14	2.80E-12
ENSBTAG00000002929	106.0193	6.5587	1.0652	6.1572	7.41E-10	2.10E-08
ENSBTAG00000037800	96.8877	6.4918	1.0661	6.0893	1.13E-09	3.11E-08
ENSBTAG00000038548	56.1929	6.4374	1.0281	6.2612	3.82E-10	1.15E-08
ENSBTAG00000021492	85.2751	6.4059	0.8989	7.1261	1.03E-12	4.64E-11
ENSBTAG00000006566	85.5741	6.3878	1.0685	5.9782	2.26E-09	5.86E-08
ENSBTAG00000018993	272.6032	6.3680	0.8370	7.6078	2.79E-14	1.55E-12
ENSBTAG00000013227	317.5898	6.3665	0.7110	8.9543	3.42E-19	3.60E-17
ENSBTAG00000002505	49.8690	6.3564	1.0270	6.1890	6.05E-10	1.74E-08
ENSBTAG00000005990	456.3003	6.3559	0.6602	9.6269	6.15E-22	9.54E-20
ENSBTAG00000005892	145.1695	6.3447	1.0164	6.2422	4.31E-10	1.28E-08
ENSBTAG00000020034	739.0268	6.3440	0.6159	10.2995	7.08E-25	1.40E-22
ENSBTAG00000016163	66.1293	6.3396	1.0548	6.0103	1.85E-09	4.93E-08
ENSBTAG00000002908	60.2767	6.3318	1.0480	6.0416	1.53E-09	4.11E-08
ENSBTAG00000007530	74.5105	6.2911	0.8900	7.0691	1.56E-12	6.87E-11
ENSBTAG00000003305	298.7140	6.2807	0.8473	7.4124	1.24E-13	6.33E-12
ENSBTAG00000006130	316.0747	6.2784	0.6496	9.6652	4.24E-22	6.72E-20
ENSBTAG00000015834	679.3553	6.2683	0.3841	16.3198	7.13E-60	1.60E-56
ENSBTAG00000018430	141.8378	6.2639	0.9262	6.7626	1.36E-11	5.25E-10
ENSBTAG00000006939	156.2200	6.2597	0.9376	6.6766	2.45E-11	9.06E-10
ENSBTAG00000018280	60.1686	6.2571	1.0568	5.9208	3.20E-09	8.09E-08
ENSBTAG00000000715	111.0114	6.2339	0.8437	7.3891	1.48E-13	7.49E-12
ENSBTAG00000017593	141.2319	6.2182	0.8034	7.7396	9.98E-15	5.85E-13
ENSBTAG00000016276	65.7804	6.2067	1.0694	5.8040	6.47E-09	1.53E-07
ENSBTAG00000015727	75.5614	6.1850	1.0812	5.7204	1.06E-08	2.43E-07
ENSBTAG00000015419	72.4418	6.1841	0.8991	6.8784	6.05E-12	2.47E-10
ENSBTAG00000025642	153.0379	6.1795	0.7028	8.7923	1.47E-18	1.38E-16
ENSBTAG00000004894	71.3999	6.1722	1.0788	5.7212	1.06E-08	2.42E-07
ENSBTAG00000011437	3137.7817	6.1668	0.5334	11.5605	6.54E-31	1.84E-28
ENSBTAG00000005628	262.1524	6.1583	0.7888	7.8075	5.83E-15	3.57E-13
ENSBTAG00000018165	423.7567	6.1518	0.7848	7.8386	4.55E-15	2.86E-13
ENSBTAG00000019432	57.6398	6.1492	1.0660	5.7687	7.99E-09	1.87E-07
ENSBTAG00000026963	89.6813	6.1490	0.9549	6.4394	1.20E-10	3.96E-09
ENSBTAG00000038154	51.8022	6.1242	1.0603	5.7758	7.66E-09	1.80E-07
ENSBTAG00000034609	64.9182	6.1149	1.0785	5.6699	1.43E-08	3.18E-07
ENSBTAG00000006453	56.2825	6.1145	1.0681	5.7246	1.04E-08	2.38E-07
ENSBTAG00000038437	75.2286	6.1053	0.9395	6.4987	8.10E-11	2.75E-09

ENSBTAG00000007758	2211.4763	6.0724	0.3602	16.8576	9.23E-64	2.49E-60
ENSBTAG00000020892	114.9622	6.0688	1.0120	5.9969	2.01E-09	5.28E-08
ENSBTAG00000018016	78.3796	6.0642	0.9585	6.3271	2.50E-10	7.89E-09
ENSBTAG00000006031	3166.5410	6.0634	0.4401	13.7764	3.53E-43	2.16E-40
ENSBTAG00000021958	50.4950	6.0608	1.0658	5.6865	1.30E-08	2.90E-07
ENSBTAG00000009064	359.4482	6.0565	0.7780	7.7845	7.00E-15	4.18E-13
ENSBTAG00000011238	401.3928	6.0386	0.7392	8.1693	3.10E-16	2.21E-14
ENSBTAG00000001186	146.0238	6.0305	1.0482	5.7532	8.75E-09	2.05E-07
ENSBTAG00000013730	81.9317	6.0302	0.9603	6.2794	3.40E-10	1.04E-08
ENSBTAG00000009850	89.2967	6.0293	0.9771	6.1707	6.80E-10	1.93E-08
ENSBTAG00000015562	96.3725	6.0284	0.8559	7.0432	1.88E-12	8.20E-11
ENSBTAG00000003255	70.2349	6.0267	0.9276	6.4971	8.19E-11	2.77E-09
ENSBTAG00000014113	82.9052	6.0174	0.9800	6.1401	8.25E-10	2.30E-08
ENSBTAG00000023283	112.1392	6.0171	0.9084	6.6239	3.50E-11	1.27E-09
ENSBTAG00000010411	85.0800	6.0109	0.9712	6.1888	6.06E-10	1.74E-08
ENSBTAG00000046768	53.1157	6.0101	1.0755	5.5880	2.30E-08	4.90E-07
ENSBTAG00000000283	481.0707	6.0077	0.5616	10.6982	1.04E-26	2.37E-24
ENSBTAG00000031397	404.1610	6.0031	0.8209	7.3130	2.61E-13	1.28E-11
ENSBTAG00000017875	331.8958	5.9859	0.8137	7.3562	1.89E-13	9.37E-12
ENSBTAG00000039119	45.0790	5.9758	1.0667	5.6020	2.12E-08	4.58E-07
ENSBTAG00000007881	607.7495	5.9727	0.8089	7.3840	1.54E-13	7.76E-12
ENSBTAG00000001219	240.5677	5.9696	0.8913	6.6977	2.12E-11	7.89E-10
ENSBTAG00000002341	288.5819	5.9646	0.9462	6.3037	2.91E-10	9.05E-09
ENSBTAG00000047727	108.2909	5.9399	0.7836	7.5803	3.45E-14	1.87E-12
ENSBTAG00000007828	207.7206	5.9378	0.7962	7.4579	8.79E-14	4.61E-12
ENSBTAG00000019519	103.4989	5.9362	0.7612	7.7987	6.26E-15	3.77E-13
ENSBTAG00000014761	2833.4555	5.9152	0.7775	7.6075	2.79E-14	1.55E-12
ENSBTAG00000017722	121.9211	5.9106	0.8493	6.9595	3.41E-12	1.43E-10
ENSBTAG00000004547	460.9535	5.9047	0.8207	7.1947	6.26E-13	2.91E-11
ENSBTAG00000023179	523.8677	5.9045	0.6573	8.9828	2.64E-19	2.82E-17
ENSBTAG00000010273	4368.7379	5.9004	0.7580	7.7844	7.00E-15	4.18E-13
ENSBTAG00000000431	115.6526	5.8634	0.6804	8.6180	6.81E-18	6.04E-16
ENSBTAG00000010448	35.6269	5.8583	1.0609	5.5218	3.36E-08	6.92E-07
ENSBTAG00000047448	185.6885	5.8566	0.8406	6.9670	3.24E-12	1.36E-10
ENSBTAG00000019975	55.6321	5.8380	0.9259	6.3053	2.88E-10	8.98E-09
ENSBTAG00000002923	234.7841	5.8241	0.9725	5.9885	2.12E-09	5.52E-08
ENSBTAG00000014016	40.7747	5.8150	1.0780	5.3944	6.88E-08	1.36E-06
ENSBTAG00000001029	124.8860	5.8073	0.9974	5.8226	5.79E-09	1.39E-07
ENSBTAG00000010023	156.9688	5.7989	0.9364	6.1926	5.92E-10	1.71E-08
ENSBTAG00000039509	96.1418	5.7981	0.7575	7.6540	1.95E-14	1.10E-12
ENSBTAG00000000653	146.7681	5.7742	0.8812	6.5529	5.64E-11	1.95E-09
ENSBTAG00000018367	42.3327	5.7569	1.0877	5.2930	1.20E-07	2.30E-06
ENSBTAG00000006582	342.6265	5.7486	0.8070	7.1238	1.05E-12	4.70E-11

ENSBTAG00000006452	124.8997	5.7443	0.7911	7.2615	3.83E-13	1.84E-11
ENSBTAG00000027204	187.9170	5.7430	0.8482	6.7706	1.28E-11	4.98E-10
ENSBTAG00000014439	59.8535	5.7330	1.1121	5.1552	2.53E-07	4.54E-06
ENSBTAG00000006240	893.8537	5.7270	0.5765	9.9340	2.96E-23	5.33E-21
ENSBTAG00000025434	17516.0994	5.7226	0.4036	14.1787	1.24E-45	9.84E-43
ENSBTAG00000001301	65.9242	5.7197	0.9945	5.7516	8.84E-09	2.06E-07
ENSBTAG00000005413	369.4671	5.7197	0.8321	6.8735	6.26E-12	2.54E-10
ENSBTAG00000016388	516.6192	5.7195	0.7272	7.8656	3.67E-15	2.37E-13
ENSBTAG00000046409	61.2862	5.7135	0.9803	5.8283	5.60E-09	1.34E-07
ENSBTAG00000023377	731.0709	5.7123	0.3670	15.5655	1.25E-54	1.87E-51
ENSBTAG00000006466	654.3450	5.7105	0.7227	7.9015	2.76E-15	1.80E-13
ENSBTAG00000002259	41.4570	5.7068	1.0919	5.2266	1.73E-07	3.21E-06
ENSBTAG00000025405	54.9222	5.6817	0.9620	5.9059	3.51E-09	8.74E-08
ENSBTAG00000001882	36.0448	5.6775	1.0847	5.2342	1.66E-07	3.09E-06
ENSBTAG00000008479	40.5624	5.6740	1.0940	5.1864	2.14E-07	3.88E-06
ENSBTAG00000006780	74.1900	5.6700	1.0256	5.5284	3.23E-08	6.71E-07
ENSBTAG00000004269	941.3128	5.6656	0.8256	6.8623	6.78E-12	2.72E-10
ENSBTAG00000012829	62.9288	5.6570	0.9979	5.6687	1.44E-08	3.19E-07
ENSBTAG00000006552	99.8226	5.6552	0.8550	6.6144	3.73E-11	1.34E-09
ENSBTAG00000033748	49.4849	5.6543	1.1091	5.0981	3.43E-07	5.90E-06
ENSBTAG00000016275	1872.9740	5.6373	0.4018	14.0310	1.01E-44	7.14E-42
ENSBTAG00000020747	355.9417	5.6261	0.5936	9.4776	2.60E-21	3.86E-19
ENSBTAG00000015844	120.9365	5.6221	1.0924	5.1468	2.65E-07	4.71E-06
ENSBTAG00000008497	52.2648	5.6190	1.1157	5.0363	4.75E-07	7.85E-06
ENSBTAG00000002006	14198.4707	5.6149	0.8350	6.7246	1.76E-11	6.65E-10
ENSBTAG00000002951	52.2307	5.6021	1.1173	5.0140	5.33E-07	8.73E-06
ENSBTAG00000040331	56.8860	5.5908	0.9750	5.7342	9.80E-09	2.26E-07
ENSBTAG00000000095	129.1531	5.5902	0.8921	6.2661	3.70E-10	1.12E-08
ENSBTAG00000033304	251.8733	5.5708	0.8613	6.4682	9.92E-11	3.30E-09
ENSBTAG00000008603	30.1651	5.5707	1.0833	5.1421	2.72E-07	4.81E-06
ENSBTAG00000009381	323.2472	5.5654	0.8890	6.2600	3.85E-10	1.16E-08
ENSBTAG00000015311	66.7405	5.5540	0.8849	6.2767	3.46E-10	1.06E-08
ENSBTAG00000034827	663.2854	5.5512	0.4048	13.7120	8.61E-43	5.05E-40
ENSBTAG00000007883	484.1313	5.5354	0.8188	6.7605	1.37E-11	5.31E-10
ENSBTAG00000015609	545.5930	5.5311	0.7756	7.1316	9.92E-13	4.47E-11
ENSBTAG00000010427	218.1002	5.5127	0.6489	8.4952	1.98E-17	1.68E-15
ENSBTAG00000011019	31.5448	5.5125	1.0941	5.0381	4.70E-07	7.79E-06
ENSBTAG00000000306	99.4737	5.5107	0.8869	6.2134	5.18E-10	1.51E-08
ENSBTAG00000014762	167.8739	5.5106	0.8616	6.3958	1.60E-10	5.22E-09
ENSBTAG00000000502	50.6477	5.4858	0.9880	5.5523	2.82E-08	5.88E-07
ENSBTAG00000048122	327.5941	5.4845	0.7140	7.6809	1.58E-14	8.99E-13
ENSBTAG00000018134	79.6727	5.4625	0.9698	5.6325	1.78E-08	3.89E-07
ENSBTAG00000012695	412.4479	5.4448	0.6592	8.2594	1.46E-16	1.11E-14

ENSBTAG00000021904	51.9322	5.4426	0.9870	5.5145	3.50E-08	7.19E-07
ENSBTAG00000047029	1323.1605	5.4393	0.5173	10.5157	7.31E-26	1.59E-23
ENSBTAG00000010300	26.2782	5.4257	1.0901	4.9774	6.44E-07	1.03E-05
ENSBTAG00000007079	1342.4463	5.4228	0.6980	7.7696	7.88E-15	4.68E-13
ENSBTAG00000015133	69.2977	5.4177	0.9096	5.9560	2.59E-09	6.64E-08
ENSBTAG00000002041	204.3453	5.4165	0.8226	6.5848	4.55E-11	1.62E-09
ENSBTAG00000014199	97.9142	5.4149	1.0133	5.3440	9.09E-08	1.77E-06
ENSBTAG00000020921	57.5908	5.4140	1.0127	5.3461	8.99E-08	1.75E-06
ENSBTAG00000014972	119.1000	5.4092	0.8005	6.7570	1.41E-11	5.43E-10
ENSBTAG00000012478	24.8282	5.4052	1.0879	4.9685	6.75E-07	1.07E-05
ENSBTAG00000011782	63.9729	5.4006	0.9195	5.8731	4.28E-09	1.04E-07
ENSBTAG00000006377	160.9074	5.4006	0.9101	5.9339	2.96E-09	7.53E-08
ENSBTAG00000035183	36.4999	5.3950	1.1175	4.8277	1.38E-06	2.06E-05
ENSBTAG00000021872	208.8955	5.3802	0.6199	8.6794	3.98E-18	3.62E-16
ENSBTAG00000038650	37.2345	5.3755	1.1208	4.7963	1.62E-06	2.36E-05
ENSBTAG00000047107	51.4756	5.3716	0.9998	5.3728	7.75E-08	1.52E-06
ENSBTAG00000010433	80.3120	5.3473	1.1556	4.6271	3.71E-06	4.97E-05
ENSBTAG00000001008	93.6416	5.3408	0.8098	6.5955	4.24E-11	1.52E-09
ENSBTAG00000001197	88.9257	5.3180	0.9216	5.7705	7.91E-09	1.86E-07
ENSBTAG00000021815	4586.2537	5.3147	0.5969	8.9040	5.39E-19	5.50E-17
ENSBTAG00000003436	8145.3800	5.3060	0.3674	14.4440	2.74E-47	2.64E-44
ENSBTAG00000010026	133.2658	5.3000	0.8705	6.0885	1.14E-09	3.12E-08
ENSBTAG00000009717	273.1533	5.2859	0.8346	6.3333	2.40E-10	7.59E-09
ENSBTAG00000026111	142.7306	5.2813	0.9157	5.7675	8.05E-09	1.88E-07
ENSBTAG00000017256	98.1490	5.2775	0.9364	5.6362	1.74E-08	3.82E-07
ENSBTAG00000000641	40.0206	5.2653	0.9681	5.4390	5.36E-08	1.07E-06
ENSBTAG00000010447	371.9605	5.2630	0.6808	7.7309	1.07E-14	6.20E-13
ENSBTAG00000014169	202.2771	5.2625	0.5694	9.2424	2.41E-20	2.85E-18
ENSBTAG00000018164	5226.5630	5.2596	0.6194	8.4918	2.03E-17	1.71E-15
ENSBTAG00000005691	3280.1343	5.2570	0.5479	9.5943	8.45E-22	1.30E-19
ENSBTAG00000019227	113.0944	5.2473	1.0672	4.9169	8.79E-07	1.38E-05
ENSBTAG00000003650	708.6262	5.2417	0.5226	10.0297	1.13E-23	2.11E-21
ENSBTAG00000015710	266.4422	5.2398	0.6552	7.9975	1.27E-15	8.60E-14
ENSBTAG00000008864	44.7064	5.2377	1.0157	5.1568	2.51E-07	4.51E-06
ENSBTAG00000047586	64.9970	5.2330	0.9563	5.4723	4.44E-08	9.01E-07
ENSBTAG00000021029	51.9847	5.2328	1.0451	5.0071	5.53E-07	8.99E-06
ENSBTAG00000016770	49.7905	5.2327	1.0376	5.0431	4.58E-07	7.63E-06
ENSBTAG00000011043	587.1969	5.2279	0.6389	8.1826	2.78E-16	2.00E-14
ENSBTAG00000019272	61.6389	5.2167	1.0583	4.9293	8.25E-07	1.29E-05
ENSBTAG00000004386	24.7261	5.2144	1.1113	4.6921	2.70E-06	3.75E-05
ENSBTAG00000046555	106.9174	5.2071	0.9175	5.6754	1.38E-08	3.08E-07
ENSBTAG00000046324	437.0088	5.2035	0.7730	6.7317	1.68E-11	6.35E-10
ENSBTAG00000007626	579.6998	5.1881	0.7118	7.2892	3.12E-13	1.51E-11

ENSBTAG00000020872	223.4640	5.1804	0.8212	6.3084	2.82E-10	8.82E-09
ENSBTAG00000017592	5832.6800	5.1742	0.4284	12.0794	1.36E-33	4.46E-31
ENSBTAG00000001209	129.9716	5.1697	0.8737	5.9171	3.28E-09	8.21E-08
ENSBTAG00000026242	25.8201	5.1664	1.1200	4.6129	3.97E-06	5.28E-05
ENSBTAG00000026326	283.0516	5.1658	0.6540	7.8983	2.83E-15	1.84E-13
ENSBTAG00000032068	25.9940	5.1647	1.1208	4.6082	4.06E-06	5.39E-05
ENSBTAG00000004322	3346.2945	5.1600	0.5448	9.4716	2.76E-21	4.00E-19
ENSBTAG00000001567	241.7337	5.1583	0.5653	9.1242	7.22E-20	8.12E-18
ENSBTAG00000047039	24.7078	5.1548	1.1183	4.6095	4.04E-06	5.36E-05
ENSBTAG00000021647	38.4542	5.1538	1.1442	4.5045	6.65E-06	8.38E-05
ENSBTAG00000004270	30.3489	5.1404	1.1331	4.5365	5.72E-06	7.29E-05
ENSBTAG00000008956	56.1036	5.1325	1.0741	4.7783	1.77E-06	2.55E-05
ENSBTAG00000005958	231.4344	5.1310	0.9379	5.4707	4.48E-08	9.08E-07
ENSBTAG00000010670	258.0085	5.1308	0.8242	6.2249	4.82E-10	1.41E-08
ENSBTAG00000047986	39.4004	5.1297	1.1475	4.4704	7.81E-06	9.70E-05
ENSBTAG00000017839	39.9770	5.1235	1.0168	5.0388	4.68E-07	7.77E-06
ENSBTAG00000011578	1001.1047	5.1227	0.7674	6.6753	2.47E-11	9.12E-10
ENSBTAG00000004680	115.6311	5.1105	0.8905	5.7389	9.53E-09	2.20E-07
ENSBTAG00000007596	7645.0201	5.1053	0.3753	13.6039	3.79E-42	2.05E-39
ENSBTAG00000014127	498.5516	5.1029	0.8118	6.2859	3.26E-10	1.00E-08
ENSBTAG00000013619	317.2504	5.0991	0.7229	7.0532	1.75E-12	7.65E-11
ENSBTAG00000034139	100.7302	5.0932	0.6093	8.3591	6.32E-17	5.04E-15
ENSBTAG00000016061	309.7472	5.0918	0.7749	6.5713	4.99E-11	1.77E-09
ENSBTAG00000000436	579.8521	5.0889	0.6291	8.0889	6.02E-16	4.16E-14
ENSBTAG00000023976	242.2018	5.0888	0.7711	6.5993	4.13E-11	1.48E-09
ENSBTAG00000003994	230.5064	5.0694	0.6961	7.2828	3.27E-13	1.58E-11
ENSBTAG00000037937	74.1433	5.0583	0.9381	5.3923	6.96E-08	1.37E-06
ENSBTAG00000006789	15255.4762	5.0568	0.4026	12.5609	3.47E-36	1.34E-33
ENSBTAG00000018984	163.8858	5.0499	0.8383	6.0240	1.70E-09	4.56E-08
ENSBTAG00000039928	126.5606	5.0469	0.8457	5.9679	2.40E-09	6.23E-08
ENSBTAG00000012847	17501.9692	5.0439	0.2987	16.8856	5.74E-64	1.94E-60
ENSBTAG00000003045	1644.9730	5.0366	0.7457	6.7541	1.44E-11	5.52E-10
ENSBTAG00000013125	697.0385	5.0297	0.7738	6.4995	8.06E-11	2.74E-09
ENSBTAG00000000711	503.2281	5.0229	0.6995	7.1803	6.96E-13	3.21E-11
ENSBTAG00000009353	268.9386	5.0118	0.7544	6.6435	3.06E-11	1.12E-09
ENSBTAG00000006707	608.9144	5.0087	0.6952	7.2044	5.83E-13	2.75E-11
ENSBTAG00000011511	209.1005	5.0072	0.8940	5.6006	2.14E-08	4.59E-07
ENSBTAG00000023338	10841.4875	5.0041	0.2256	22.1815	5.18E-109	6.99E-105
ENSBTAG00000002435	116.1260	4.9809	1.0752	4.6326	3.61E-06	4.85E-05
ENSBTAG00000016944	32.8819	4.9777	0.9885	5.0355	4.77E-07	7.87E-06
ENSBTAG00000005018	105.2350	4.9667	0.8369	5.9348	2.94E-09	7.51E-08
ENSBTAG00000006194	107.2551	4.9577	0.8790	5.6400	1.70E-08	3.75E-07
ENSBTAG00000021842	30.9595	4.9537	1.1525	4.2981	1.72E-05	1.97E-04

ENSBTAG00000020225	64.4067	4.9482	0.9218	5.3682	7.95E-08	1.56E-06
ENSBTAG00000045880	94.7082	4.9437	1.0463	4.7249	2.30E-06	3.22E-05
ENSBTAG00000026779	1478.8797	4.9396	0.8070	6.1211	9.29E-10	2.57E-08
ENSBTAG00000012817	419.0302	4.9329	0.4158	11.8645	1.81E-32	5.67E-30
ENSBTAG00000012406	70.9017	4.9309	0.9766	5.0490	4.44E-07	7.42E-06
ENSBTAG00000018193	117.3086	4.9231	0.7701	6.3927	1.63E-10	5.30E-09
ENSBTAG00000011896	21.2465	4.9146	1.1357	4.3274	1.51E-05	1.75E-04
ENSBTAG00000006851	24.9867	4.9098	1.1459	4.2846	1.83E-05	2.07E-04
ENSBTAG00000020880	8776.5526	4.9022	0.3201	15.3139	6.17E-53	7.57E-50
ENSBTAG00000026825	10355.2244	4.8775	0.3730	13.0767	4.47E-39	1.89E-36
ENSBTAG00000019832	337.5933	4.8707	0.9796	4.9720	6.63E-07	1.06E-05
ENSBTAG00000033565	238.4475	4.8702	0.7168	6.7948	1.08E-11	4.25E-10
ENSBTAG00000031302	23.7410	4.8635	1.1479	4.2370	2.27E-05	2.49E-04
ENSBTAG00000006902	22.4172	4.8543	1.1455	4.2376	2.26E-05	2.49E-04
ENSBTAG00000005234	535.1171	4.8516	0.7717	6.2871	3.23E-10	9.98E-09
ENSBTAG00000000838	278.9035	4.8494	0.5988	8.0983	5.57E-16	3.87E-14
ENSBTAG00000024470	48.8652	4.8490	0.9601	5.0504	4.41E-07	7.38E-06
ENSBTAG00000011784	698.6093	4.8425	0.6847	7.0724	1.52E-12	6.73E-11
ENSBTAG00000047529	21.6299	4.8392	1.1451	4.2260	2.38E-05	2.61E-04
ENSBTAG00000016915	249.7909	4.8360	0.7618	6.3480	2.18E-10	6.93E-09
ENSBTAG00000004305	190.9447	4.8342	1.0530	4.5909	4.41E-06	5.80E-05
ENSBTAG00000018403	52.6732	4.8300	1.0195	4.7376	2.16E-06	3.05E-05
ENSBTAG00000003297	52.3410	4.8253	1.1117	4.3403	1.42E-05	1.67E-04
ENSBTAG00000008631	994.6071	4.8202	0.6748	7.1435	9.10E-13	4.13E-11
ENSBTAG00000018765	1003.3590	4.8109	0.5417	8.8815	6.60E-19	6.69E-17
ENSBTAG00000001443	109.5537	4.8099	0.7997	6.0150	1.80E-09	4.80E-08
ENSBTAG00000022920	24151.9735	4.8055	0.5503	8.7328	2.49E-18	2.31E-16
ENSBTAG00000034366	642.7311	4.8042	0.8060	5.9604	2.52E-09	6.49E-08
ENSBTAG00000010704	63.6864	4.7912	0.9361	5.1185	3.08E-07	5.39E-06
ENSBTAG00000010664	73.3043	4.7850	0.9966	4.8013	1.58E-06	2.31E-05
ENSBTAG00000011411	58.9655	4.7834	0.9430	5.0724	3.93E-07	6.68E-06
ENSBTAG00000005120	23.7657	4.7800	1.1564	4.1337	3.57E-05	3.72E-04
ENSBTAG00000024272	91.5317	4.7780	0.9511	5.0238	5.07E-07	8.32E-06
ENSBTAG00000016345	74.4729	4.7744	0.8066	5.9189	3.24E-09	8.15E-08
ENSBTAG00000005193	19.0023	4.7740	1.1444	4.1717	3.02E-05	3.23E-04
ENSBTAG00000020736	195.5608	4.7722	0.8105	5.8877	3.92E-09	9.62E-08
ENSBTAG00000008897	255.7779	4.7614	0.9163	5.1962	2.03E-07	3.70E-06
ENSBTAG00000015043	186.3033	4.7603	0.7933	6.0008	1.96E-09	5.19E-08
ENSBTAG00000039813	19.6162	4.7596	1.1479	4.1463	3.38E-05	3.56E-04
ENSBTAG00000000432	232.5841	4.7431	0.7717	6.1460	7.95E-10	2.23E-08
ENSBTAG00000030024	21.4730	4.7413	1.1550	4.1052	4.04E-05	4.15E-04
ENSBTAG00000027727	21.8195	4.7391	1.1559	4.1000	4.13E-05	4.23E-04
ENSBTAG00000039372	16.8426	4.7345	1.1411	4.1490	3.34E-05	3.52E-04

ENSBTAG00000032089	35.9666	4.7308	0.9247	5.1162	3.12E-07	5.43E-06
ENSBTAG00000011734	112.3828	4.7303	0.9102	5.1971	2.02E-07	3.70E-06
ENSBTAG00000014441	35.3367	4.7280	1.0562	4.4766	7.59E-06	9.45E-05
ENSBTAG00000011115	160.8593	4.7268	0.9705	4.8706	1.11E-06	1.72E-05
ENSBTAG00000002096	189.0155	4.7143	0.8417	5.6007	2.13E-08	4.59E-07
ENSBTAG00000020355	454.5505	4.7128	0.7378	6.3873	1.69E-10	5.45E-09
ENSBTAG00000012052	33.3320	4.7117	1.1767	4.0042	6.22E-05	6.07E-04
ENSBTAG00000018774	88.3642	4.7097	0.6540	7.2016	5.95E-13	2.80E-11
ENSBTAG00000006859	24.1786	4.7045	1.1645	4.0400	5.35E-05	5.29E-04
ENSBTAG00000021672	96.6246	4.7017	0.9933	4.7334	2.21E-06	3.10E-05
ENSBTAG00000001444	166.3830	4.7002	0.9022	5.2098	1.89E-07	3.48E-06
ENSBTAG00000013452	22.4228	4.6991	1.1614	4.0460	5.21E-05	5.17E-04
ENSBTAG00000013333	42.0842	4.6977	1.1045	4.2532	2.11E-05	2.35E-04
ENSBTAG00000018015	31.1991	4.6974	1.0390	4.5211	6.15E-06	7.80E-05
ENSBTAG00000006685	27.3789	4.6933	1.1709	4.0083	6.12E-05	5.99E-04
ENSBTAG00000030084	29.5590	4.6931	1.0474	4.4805	7.45E-06	9.29E-05
ENSBTAG00000013689	38.3357	4.6895	1.0766	4.3558	1.33E-05	1.57E-04
ENSBTAG00000037510	46.7588	4.6889	0.9902	4.7351	2.19E-06	3.08E-05
ENSBTAG00000006143	119.4404	4.6779	0.8013	5.8379	5.29E-09	1.27E-07
ENSBTAG00000006601	407.7806	4.6762	0.3963	11.7997	3.92E-32	1.17E-29
ENSBTAG00000017280	240.3719	4.6756	0.5977	7.8225	5.18E-15	3.19E-13
ENSBTAG00000019212	61.8440	4.6713	1.1351	4.1154	3.86E-05	3.99E-04
ENSBTAG00000048268	32.5741	4.6641	1.1797	3.9535	7.70E-05	7.27E-04
ENSBTAG00000011011	149.0978	4.6640	0.7564	6.1659	7.01E-10	1.99E-08
ENSBTAG00000000812	44.9617	4.6572	1.1186	4.1635	3.13E-05	3.34E-04
ENSBTAG00000035224	32.3434	4.6371	1.1816	3.9242	8.70E-05	8.11E-04
ENSBTAG00000010284	32.8323	4.6348	0.9215	5.0298	4.91E-07	8.07E-06
ENSBTAG00000000648	23.5992	4.6324	1.1701	3.9590	7.53E-05	7.14E-04
ENSBTAG00000002596	37.7696	4.6320	1.1867	3.9032	9.49E-05	8.76E-04
ENSBTAG00000007846	27.1034	4.6204	1.0448	4.4222	9.77E-06	1.19E-04
ENSBTAG00000006606	52.4218	4.6157	1.1394	4.0510	5.10E-05	5.07E-04
ENSBTAG00000001725	73.0893	4.5951	1.1562	3.9743	7.06E-05	6.77E-04
ENSBTAG00000021435	1103.6723	4.5949	0.8180	5.6171	1.94E-08	4.22E-07
ENSBTAG00000030426	17.2039	4.5902	1.1585	3.9621	7.43E-05	7.06E-04
ENSBTAG00000021901	56.1417	4.5896	0.9703	4.7300	2.25E-06	3.15E-05
ENSBTAG00000013429	18.8263	4.5817	1.1643	3.9353	8.31E-05	7.76E-04
ENSBTAG00000012737	37.2447	4.5791	0.9887	4.6313	3.63E-06	4.88E-05
ENSBTAG00000019517	217.3514	4.5660	0.9518	4.7973	1.61E-06	2.35E-05
ENSBTAG00000014217	48.2705	4.5636	1.0449	4.3675	1.26E-05	1.49E-04
ENSBTAG00000018203	52.2790	4.5624	0.9874	4.6206	3.83E-06	5.11E-05
ENSBTAG00000011563	23.6252	4.5605	1.0088	4.5207	6.16E-06	7.80E-05
ENSBTAG00000010303	526.5848	4.5538	0.7578	6.0095	1.86E-09	4.94E-08
ENSBTAG00000007921	49.3156	4.5511	0.9702	4.6910	2.72E-06	3.76E-05

ENSBTAG00000014554	44.7854	4.5439	1.1338	4.0075	6.14E-05	6.00E-04
ENSBTAG00000014038	26.3794	4.5413	1.0569	4.2967	1.73E-05	1.98E-04
ENSBTAG00000027051	601.7902	4.5379	0.8441	5.3763	7.60E-08	1.50E-06
ENSBTAG00000035868	30.6031	4.5325	1.0682	4.2430	2.21E-05	2.44E-04
ENSBTAG00000005501	1874.0293	4.5298	0.8220	5.5104	3.58E-08	7.34E-07
ENSBTAG00000047121	16.0930	4.5167	1.1626	3.8849	1.02E-04	9.37E-04
ENSBTAG00000009614	100.0067	4.5149	0.8063	5.5997	2.15E-08	4.61E-07
ENSBTAG00000003382	47.2360	4.5038	0.9172	4.9101	9.10E-07	1.42E-05
ENSBTAG00000002333	117.8082	4.4994	0.9018	4.9895	6.05E-07	9.80E-06
ENSBTAG00000018994	27.5068	4.4944	1.0566	4.2537	2.10E-05	2.35E-04
ENSBTAG00000038985	14.8645	4.4899	1.1608	3.8679	1.10E-04	9.97E-04
ENSBTAG00000018869	135.3945	4.4886	0.8393	5.3480	8.89E-08	1.74E-06
ENSBTAG00000017473	39.1767	4.4873	1.1275	3.9797	6.90E-05	6.64E-04
ENSBTAG00000046152	24.9383	4.4817	1.1855	3.7805	1.57E-04	1.36E-03
ENSBTAG00000037989	41.9615	4.4805	1.0315	4.3438	1.40E-05	1.65E-04
ENSBTAG00000010452	82.9026	4.4782	1.0993	4.0736	4.63E-05	4.65E-04
ENSBTAG00000003961	31.2000	4.4770	1.0821	4.1375	3.51E-05	3.68E-04
ENSBTAG00000016615	6947.2718	4.4749	0.5143	8.7004	3.31E-18	3.03E-16
ENSBTAG00000017138	21.0818	4.4713	1.1800	3.7892	1.51E-04	1.32E-03
ENSBTAG00000007927	25.3470	4.4711	1.0453	4.2774	1.89E-05	2.13E-04
ENSBTAG00000031647	38.3084	4.4621	1.0116	4.4111	1.03E-05	1.25E-04
ENSBTAG00000006703	34.1140	4.4602	1.1149	4.0006	6.32E-05	6.13E-04
ENSBTAG00000000357	73.0759	4.4559	0.6336	7.0327	2.03E-12	8.76E-11
ENSBTAG00000013241	27.7692	4.4538	0.9159	4.8628	1.16E-06	1.77E-05
ENSBTAG00000014798	13.2733	4.4509	1.1585	3.8420	1.22E-04	1.09E-03
ENSBTAG00000015060	148.7272	4.4460	0.9188	4.8392	1.30E-06	1.96E-05
ENSBTAG00000001154	606.2876	4.4443	0.6166	7.2080	5.68E-13	2.70E-11
ENSBTAG00000032277	16.1799	4.4403	1.1709	3.7924	1.49E-04	1.30E-03
ENSBTAG00000000842	77.4983	4.4367	0.7513	5.9055	3.52E-09	8.75E-08
ENSBTAG00000015032	1044.1559	4.4331	0.8180	5.4197	5.97E-08	1.19E-06
ENSBTAG00000012046	3742.7196	4.4276	0.4718	9.3848	6.30E-21	8.94E-19
ENSBTAG00000014088	45.7249	4.4222	0.8581	5.1536	2.55E-07	4.57E-06
ENSBTAG00000017040	84.5482	4.4208	0.7056	6.2654	3.72E-10	1.13E-08
ENSBTAG00000021249	62.8625	4.4044	0.9808	4.4907	7.10E-06	8.91E-05
ENSBTAG00000031020	19.8113	4.3918	1.1846	3.7074	2.09E-04	1.74E-03
ENSBTAG00000018146	60.2887	4.3875	1.0008	4.3841	1.16E-05	1.39E-04
ENSBTAG00000039466	1300.4519	4.3873	0.4631	9.4732	2.71E-21	3.98E-19
ENSBTAG00000021709	355.1579	4.3869	0.8655	5.0687	4.01E-07	6.78E-06
ENSBTAG00000004608	16.2910	4.3808	1.1771	3.7217	1.98E-04	1.66E-03
ENSBTAG00000012007	546.6957	4.3795	0.4179	10.4791	1.08E-25	2.31E-23
ENSBTAG00000008008	463.8275	4.3640	0.7219	6.0449	1.49E-09	4.04E-08
ENSBTAG00000031750	266.8580	4.3600	0.6994	6.2338	4.55E-10	1.35E-08
ENSBTAG00000039347	19.5861	4.3495	1.1879	3.6615	2.51E-04	2.03E-03

ENSBTAG00000038494	21.7736	4.3403	1.1925	3.6396	2.73E-04	2.17E-03
ENSBTAG00000010047	31.8096	4.3231	1.0151	4.2589	2.05E-05	2.30E-04
ENSBTAG00000004337	27.0533	4.3193	0.9568	4.5142	6.36E-06	8.04E-05
ENSBTAG00000005647	30.3401	4.3172	0.9625	4.4854	7.28E-06	9.10E-05
ENSBTAG00000009156	37.5143	4.3136	0.9246	4.6656	3.08E-06	4.19E-05
ENSBTAG00000018744	5026.7370	4.3120	0.5279	8.1683	3.13E-16	2.22E-14
ENSBTAG00000019524	18.5337	4.3090	1.0300	4.1835	2.87E-05	3.08E-04
ENSBTAG00000020676	27.3285	4.3083	1.1093	3.8836	1.03E-04	9.41E-04
ENSBTAG00000000820	71.2845	4.2950	1.1908	3.6068	3.10E-04	2.41E-03
ENSBTAG00000007531	187.5867	4.2874	0.9234	4.6430	3.43E-06	4.64E-05
ENSBTAG00000032289	72.8336	4.2865	0.8597	4.9862	6.16E-07	9.94E-06
ENSBTAG00000004741	14.4277	4.2837	1.1809	3.6275	2.86E-04	2.26E-03
ENSBTAG00000011481	46.7883	4.2836	1.0826	3.9567	7.60E-05	7.19E-04
ENSBTAG00000010349	704.8808	4.2818	0.5453	7.8520	4.09E-15	2.59E-13
ENSBTAG00000010963	62.9913	4.2750	0.9195	4.6493	3.33E-06	4.51E-05
ENSBTAG00000027444	171.0408	4.2739	0.7644	5.5910	2.26E-08	4.83E-07
ENSBTAG00000022759	15.8569	4.2715	1.1862	3.6008	3.17E-04	2.46E-03
ENSBTAG00000003276	97.4279	4.2635	0.8319	5.1249	2.98E-07	5.23E-06
ENSBTAG00000013066	2306.8072	4.2623	0.8360	5.0984	3.43E-07	5.90E-06
ENSBTAG00000000240	2070.5105	4.2598	0.4359	9.7728	1.47E-22	2.48E-20
ENSBTAG00000009012	4199.1588	4.2577	1.1000	3.8706	1.09E-04	9.88E-04
ENSBTAG00000010057	22.2148	4.2576	1.1998	3.5486	3.87E-04	2.93E-03
ENSBTAG00000008550	181.8993	4.2566	0.9634	4.4182	9.95E-06	1.21E-04
ENSBTAG00000005622	6656.0757	4.2528	0.3037	14.0023	1.51E-44	1.02E-41
ENSBTAG00000008555	18.2566	4.2511	1.1938	3.5610	3.69E-04	2.81E-03
ENSBTAG00000012223	14.2116	4.2489	1.1836	3.5898	3.31E-04	2.55E-03
ENSBTAG00000016593	496.6370	4.2463	0.7036	6.0353	1.59E-09	4.26E-08
ENSBTAG00000000581	98.3827	4.2458	0.7976	5.3231	1.02E-07	1.97E-06
ENSBTAG00000007635	14.0606	4.2429	1.1837	3.5844	3.38E-04	2.60E-03
ENSBTAG00000039929	89.2484	4.2417	0.8201	5.1719	2.32E-07	4.18E-06
ENSBTAG00000002917	7294.5155	4.2404	0.6099	6.9529	3.58E-12	1.48E-10
ENSBTAG00000004558	179.9992	4.2389	0.7023	6.0361	1.58E-09	4.25E-08
ENSBTAG00000046733	53.1443	4.2318	0.8316	5.0886	3.61E-07	6.16E-06
ENSBTAG00000021445	44.9021	4.2298	0.9198	4.5986	4.25E-06	5.61E-05
ENSBTAG00000002585	30.5726	4.2274	0.8507	4.9695	6.71E-07	1.07E-05
ENSBTAG00000030340	134.9895	4.2265	0.7742	5.4590	4.79E-08	9.67E-07
ENSBTAG00000019788	29.1537	4.2176	1.1332	3.7220	1.98E-04	1.66E-03
ENSBTAG00000012020	29.7968	4.2060	1.1218	3.7494	1.77E-04	1.51E-03
ENSBTAG00000005313	189.5824	4.2054	0.6686	6.2899	3.18E-10	9.87E-09
ENSBTAG00000009691	47.9834	4.1928	0.8202	5.1121	3.19E-07	5.53E-06
ENSBTAG00000011236	87.9761	4.1884	0.8411	4.9795	6.38E-07	1.02E-05
ENSBTAG00000007300	311.7247	4.1805	0.5402	7.7391	1.00E-14	5.85E-13
ENSBTAG00000019567	35.3391	4.1796	0.9693	4.3121	1.62E-05	1.87E-04

ENSBTAG00000006039	690.8921	4.1795	0.8265	5.0568	4.26E-07	7.16E-06
ENSBTAG00000014560	40.9786	4.1742	0.9150	4.5619	5.07E-06	6.56E-05
ENSBTAG00000008471	171.4890	4.1733	0.9106	4.5831	4.58E-06	6.00E-05
ENSBTAG00000037735	266.3541	4.1712	0.8261	5.0494	4.43E-07	7.41E-06
ENSBTAG00000018810	1618.1637	4.1688	0.9046	4.6084	4.06E-06	5.38E-05
ENSBTAG00000010521	20.8556	4.1680	1.0712	3.8911	9.98E-05	9.17E-04
ENSBTAG00000009125	11.6356	4.1653	1.1822	3.5233	4.26E-04	3.18E-03
ENSBTAG00000001410	16073.3931	4.1648	0.6283	6.6285	3.39E-11	1.23E-09
ENSBTAG00000015157	20.9114	4.1642	1.0725	3.8828	1.03E-04	9.44E-04
ENSBTAG00000000571	40.8857	4.1637	1.0109	4.1190	3.81E-05	3.93E-04
ENSBTAG00000026080	19.5124	4.1535	1.2040	3.4497	5.61E-04	4.04E-03
ENSBTAG00000008959	115.7302	4.1530	0.6486	6.4032	1.52E-10	4.99E-09
ENSBTAG00000012419	44.6598	4.1525	0.9664	4.2967	1.73E-05	1.98E-04
ENSBTAG00000023648	28.7540	4.1462	1.2142	3.4146	6.39E-04	4.50E-03
ENSBTAG00000023372	72.6267	4.1447	0.7948	5.2146	1.84E-07	3.39E-06
ENSBTAG00000013147	17.4543	4.1438	1.0580	3.9165	8.98E-05	8.33E-04
ENSBTAG00000000005	178.6587	4.1428	0.6440	6.4325	1.26E-10	4.13E-09
ENSBTAG00000014707	3933.8267	4.1418	0.8251	5.0197	5.18E-07	8.49E-06
ENSBTAG00000019633	113.9207	4.1412	0.6420	6.4504	1.12E-10	3.70E-09
ENSBTAG00000019711	27.0432	4.1369	1.0195	4.0578	4.95E-05	4.94E-04
ENSBTAG00000037527	170.2118	4.1358	0.8534	4.8462	1.26E-06	1.89E-05
ENSBTAG00000014823	80439.8048	4.1281	0.5476	7.5379	4.78E-14	2.59E-12
ENSBTAG00000018059	20.8331	4.1261	1.0993	3.7535	1.74E-04	1.49E-03
ENSBTAG00000046727	26.4480	4.1237	1.0162	4.0579	4.95E-05	4.94E-04
ENSBTAG00000009848	23.6066	4.1163	1.1215	3.6702	2.42E-04	1.97E-03
ENSBTAG00000016906	42.1572	4.1153	0.7863	5.2337	1.66E-07	3.09E-06
ENSBTAG00000014228	56.7041	4.1142	0.9928	4.1441	3.41E-05	3.58E-04
ENSBTAG00000040399	35.5405	4.1105	0.9774	4.2058	2.60E-05	2.82E-04
ENSBTAG00000025792	13.0149	4.1054	1.1935	3.4399	5.82E-04	4.17E-03
ENSBTAG00000020455	10763.5616	4.1052	0.2197	18.6833	6.78E-78	4.57E-74
ENSBTAG00000017390	16.1936	4.1043	1.0287	3.9899	6.61E-05	6.39E-04
ENSBTAG00000013736	39.1019	4.0977	1.2224	3.3521	8.02E-04	5.44E-03
ENSBTAG00000001325	11.1758	4.0932	1.1877	3.4465	5.68E-04	4.08E-03
ENSBTAG00000002699	792.7419	4.0932	0.9509	4.3046	1.67E-05	1.93E-04
ENSBTAG00000013957	14.2706	4.0916	1.1985	3.4139	6.40E-04	4.51E-03
ENSBTAG00000002859	16.4270	4.0866	1.0576	3.8639	1.12E-04	1.01E-03
ENSBTAG00000011584	1117.0100	4.0821	0.6807	5.9972	2.01E-09	5.28E-08
ENSBTAG00000006694	39.3720	4.0740	1.0924	3.7294	1.92E-04	1.62E-03
ENSBTAG00000000244	345.1683	4.0712	1.0161	4.0066	6.16E-05	6.02E-04
ENSBTAG00000005218	51.1785	4.0709	1.0284	3.9585	7.54E-05	7.15E-04
ENSBTAG00000045849	330.7912	4.0701	0.6822	5.9663	2.43E-09	6.28E-08
ENSBTAG00000006797	141.1933	4.0665	0.7138	5.6969	1.22E-08	2.76E-07
ENSBTAG00000014455	684.1043	4.0650	0.4708	8.6345	5.90E-18	5.34E-16

ENSBTAG00000038495	96.0300	4.0621	1.0559	3.8470	1.20E-04	1.07E-03
ENSBTAG00000021145	89.0020	4.0595	0.8042	5.0481	4.46E-07	7.45E-06
ENSBTAG00000020674	223.5519	4.0561	0.7117	5.6990	1.21E-08	2.74E-07
ENSBTAG00000048155	783.7776	4.0557	1.1877	3.4148	6.38E-04	4.50E-03
ENSBTAG00000014612	190.5284	4.0545	0.8751	4.6330	3.60E-06	4.85E-05
ENSBTAG00000012623	189.8271	4.0542	0.6190	6.5498	5.76E-11	1.99E-09
ENSBTAG00000006155	47.3349	4.0527	1.0665	3.8001	1.45E-04	1.27E-03
ENSBTAG00000005290	18.0372	4.0517	1.0663	3.7998	1.45E-04	1.27E-03
ENSBTAG00000003334	98.0411	4.0503	0.7987	5.0708	3.96E-07	6.73E-06
ENSBTAG00000021215	692.6760	4.0426	0.8898	4.5430	5.55E-06	7.11E-05
ENSBTAG00000005477	2360.4130	4.0406	0.6275	6.4392	1.20E-10	3.96E-09
ENSBTAG00000013368	60.0560	4.0366	0.9453	4.2703	1.95E-05	2.19E-04
ENSBTAG00000046280	11.7901	4.0340	1.1960	3.3729	7.44E-04	5.12E-03
ENSBTAG00000013472	4098.3256	4.0332	0.8615	4.6818	2.84E-06	3.92E-05
ENSBTAG00000014090	301.1245	4.0289	0.7453	5.4058	6.45E-08	1.28E-06
ENSBTAG00000012626	4561.4211	4.0241	0.2914	13.8091	2.25E-43	1.44E-40
ENSBTAG00000012526	278.0592	4.0216	0.7002	5.7435	9.27E-09	2.16E-07
ENSBTAG00000005021	290.1242	4.0198	0.6837	5.8792	4.12E-09	1.01E-07
ENSBTAG00000045877	1317.9289	4.0151	0.2979	13.4775	2.12E-41	1.06E-38
ENSBTAG00000012349	18.7598	4.0144	1.2135	3.3080	9.40E-04	6.16E-03
ENSBTAG00000004580	20.4194	4.0142	1.2157	3.3019	9.60E-04	6.26E-03
ENSBTAG00000012674	6428.7515	4.0044	0.5999	6.6747	2.48E-11	9.13E-10
ENSBTAG00000031444	56.8334	4.0017	0.8624	4.6403	3.48E-06	4.69E-05
ENSBTAG00000024394	15.4084	4.0014	1.2088	3.3102	9.32E-04	6.13E-03
ENSBTAG00000013201	88.3757	4.0007	0.8574	4.6660	3.07E-06	4.19E-05
ENSBTAG00000026067	20.6619	3.9978	1.1040	3.6214	2.93E-04	2.30E-03
ENSBTAG00000014496	14.8940	3.9971	1.2081	3.3086	9.38E-04	6.15E-03
ENSBTAG00000032125	15.6835	3.9961	1.0693	3.7370	1.86E-04	1.58E-03
ENSBTAG00000017617	158.4949	3.9916	0.6712	5.9466	2.74E-09	7.00E-08
ENSBTAG00000007280	22.5538	3.9872	1.0048	3.9682	7.24E-05	6.92E-04
ENSBTAG00000004917	18.4297	3.9764	1.2159	3.2703	1.07E-03	6.87E-03
ENSBTAG00000014046	26.6775	3.9762	1.0613	3.7466	1.79E-04	1.53E-03
ENSBTAG00000000816	1468.6712	3.9694	0.3952	10.0441	9.76E-24	1.85E-21
ENSBTAG00000036154	15.3533	3.9670	1.0715	3.7022	2.14E-04	1.77E-03
ENSBTAG00000013670	118.3440	3.9611	0.8210	4.8248	1.40E-06	2.08E-05
ENSBTAG00000003707	41.0170	3.9558	1.0034	3.9423	8.07E-05	7.58E-04
ENSBTAG00000046461	10.5929	3.9534	1.1990	3.2971	9.77E-04	6.35E-03
ENSBTAG00000008819	9.7040	3.9465	1.1957	3.3006	9.65E-04	6.29E-03
ENSBTAG00000038464	22.7962	3.9445	1.0221	3.8594	1.14E-04	1.03E-03
ENSBTAG00000020990	16.2606	3.9428	1.0904	3.6157	2.99E-04	2.35E-03
ENSBTAG00000021283	18.0672	3.9359	1.0924	3.6029	3.15E-04	2.45E-03
ENSBTAG00000005049	9.4606	3.9357	1.1956	3.2919	9.95E-04	6.45E-03
ENSBTAG00000046144	10.2167	3.9072	1.2019	3.2508	1.15E-03	7.26E-03

ENSBTAG00000022819	21.8093	3.9061	1.0191	3.8329	1.27E-04	1.13E-03
ENSBTAG00000025782	19.8396	3.9018	0.9842	3.9644	7.36E-05	7.02E-04
ENSBTAG00000006608	67.4652	3.9000	0.8138	4.7924	1.65E-06	2.40E-05
ENSBTAG00000003668	29.0604	3.8967	0.9726	4.0063	6.17E-05	6.02E-04
ENSBTAG00000008223	127.5209	3.8935	0.9997	3.8945	9.84E-05	9.06E-04
ENSBTAG00000012931	2250.6450	3.8842	0.3403	11.4129	3.60E-30	9.72E-28
ENSBTAG00000009493	383.4560	3.8837	0.7443	5.2177	1.81E-07	3.35E-06
ENSBTAG00000031184	2224.8107	3.8833	1.1031	3.5204	4.31E-04	3.21E-03
ENSBTAG00000014151	103.2563	3.8830	0.8248	4.7075	2.51E-06	3.48E-05
ENSBTAG00000039237	38.5161	3.8823	1.2342	3.1457	1.66E-03	9.78E-03
ENSBTAG00000010032	66.8218	3.8808	1.2148	3.1945	1.40E-03	8.53E-03
ENSBTAG00000008636	1416.6220	3.8755	1.2145	3.1911	1.42E-03	8.59E-03
ENSBTAG00000004557	13.6568	3.8747	1.2153	3.1884	1.43E-03	8.66E-03
ENSBTAG00000020313	81.6640	3.8725	0.9266	4.1794	2.92E-05	3.13E-04
ENSBTAG00000007409	31.3296	3.8714	1.0304	3.7571	1.72E-04	1.47E-03
ENSBTAG00000007592	67.2580	3.8685	0.8452	4.5769	4.72E-06	6.14E-05
ENSBTAG00000031524	33.5704	3.8652	0.8887	4.3494	1.36E-05	1.61E-04
ENSBTAG00000031998	584.3988	3.8632	0.7083	5.4540	4.93E-08	9.91E-07
ENSBTAG00000010027	16.8841	3.8595	1.2221	3.1580	1.59E-03	9.44E-03
ENSBTAG00000014725	56.2834	3.8585	0.8289	4.6552	3.24E-06	4.39E-05
ENSBTAG00000012700	136.2740	3.8563	0.6767	5.6984	1.21E-08	2.74E-07
ENSBTAG00000009292	19.9199	3.8556	1.2260	3.1449	1.66E-03	9.80E-03
ENSBTAG00000006541	287.2172	3.8531	0.9458	4.0740	4.62E-05	4.65E-04
ENSBTAG00000002190	146.9705	3.8506	0.8686	4.4332	9.28E-06	1.13E-04
ENSBTAG00000001060	200.9029	3.8501	0.8983	4.2862	1.82E-05	2.06E-04
ENSBTAG00000006864	594.5267	3.8454	0.7623	5.0446	4.54E-07	7.57E-06
ENSBTAG00000000706	2573.7218	3.8447	0.5065	7.5906	3.18E-14	1.74E-12
ENSBTAG00000010888	382.1166	3.8445	0.7601	5.0577	4.24E-07	7.14E-06
ENSBTAG00000008142	353.7349	3.8428	0.6144	6.2542	4.00E-10	1.19E-08
ENSBTAG00000039588	22.1431	3.8351	1.1429	3.3556	7.92E-04	5.40E-03
ENSBTAG00000046076	10.0837	3.8333	1.2083	3.1726	1.51E-03	9.05E-03
ENSBTAG00000020172	21.2393	3.8328	1.0135	3.7819	1.56E-04	1.35E-03
ENSBTAG00000004587	376.6274	3.8306	0.5913	6.4782	9.28E-11	3.11E-09
ENSBTAG00000000381	21.8528	3.8290	1.1422	3.3523	8.01E-04	5.44E-03
ENSBTAG00000013919	432.3084	3.8288	0.7213	5.3083	1.11E-07	2.12E-06
ENSBTAG00000011095	44.7207	3.8288	1.1641	3.2889	1.01E-03	6.49E-03
ENSBTAG00000030775	47.0307	3.8285	0.8015	4.7767	1.78E-06	2.57E-05
ENSBTAG00000011518	14.3433	3.8274	1.0687	3.5815	3.42E-04	2.62E-03
ENSBTAG00000010645	1929.3493	3.8248	0.7917	4.8314	1.36E-06	2.03E-05
ENSBTAG00000039160	162.5770	3.8234	0.9261	4.1285	3.65E-05	3.79E-04
ENSBTAG00000007581	100.0186	3.8223	0.8865	4.3117	1.62E-05	1.87E-04
ENSBTAG00000038490	23.5084	3.8195	1.0694	3.5716	3.55E-04	2.72E-03
ENSBTAG00000003312	10997.0930	3.8177	0.8055	4.7398	2.14E-06	3.02E-05

ENSBTAG00000017826	42.3465	3.8144	1.0447	3.6511	2.61E-04	2.10E-03
ENSBTAG00000021855	128.1669	3.8074	0.4861	7.8322	4.79E-15	2.99E-13
ENSBTAG00000007990	19.2154	3.8041	1.0069	3.7780	1.58E-04	1.37E-03
ENSBTAG00000013064	18.4053	3.8007	1.1409	3.3313	8.64E-04	5.76E-03
ENSBTAG00000014698	43.8158	3.7842	1.0077	3.7551	1.73E-04	1.48E-03
ENSBTAG00000043695	37.8612	3.7830	1.1625	3.2542	1.14E-03	7.18E-03
ENSBTAG00000017287	118.3858	3.7780	0.8698	4.3436	1.40E-05	1.65E-04
ENSBTAG00000047449	70.9275	3.7700	0.9377	4.0206	5.81E-05	5.71E-04
ENSBTAG00000010693	1898.0835	3.7637	0.7162	5.2550	1.48E-07	2.78E-06
ENSBTAG00000007213	1607.1302	3.7564	0.8703	4.3162	1.59E-05	1.84E-04
ENSBTAG00000003219	2618.6171	3.7522	0.4357	8.6126	7.14E-18	6.26E-16
ENSBTAG00000047676	52.9694	3.7485	0.9473	3.9571	7.59E-05	7.18E-04
ENSBTAG00000014898	90.3875	3.7480	0.8319	4.5054	6.63E-06	8.36E-05
ENSBTAG00000013205	256.0784	3.7478	0.3994	9.3836	6.38E-21	8.95E-19
ENSBTAG00000038326	5959.3974	3.7454	0.7171	5.2229	1.76E-07	3.26E-06
ENSBTAG00000004037	5796.3380	3.7401	0.4543	8.2322	1.84E-16	1.35E-14
ENSBTAG00000000995	1805.1193	3.7363	0.3727	10.0247	1.19E-23	2.19E-21
ENSBTAG00000031851	274.6027	3.7301	0.7353	5.0729	3.92E-07	6.67E-06
ENSBTAG00000022890	28.6922	3.7296	1.0458	3.5663	3.62E-04	2.76E-03
ENSBTAG00000044027	30.4040	3.7251	1.1150	3.3408	8.35E-04	5.62E-03
ENSBTAG00000036260	42.5291	3.7225	0.9338	3.9866	6.70E-05	6.47E-04
ENSBTAG00000015952	23.7156	3.7202	1.0972	3.3905	6.98E-04	4.85E-03
ENSBTAG00000031658	4295.0623	3.7191	0.7984	4.6582	3.19E-06	4.33E-05
ENSBTAG00000015711	1296.6977	3.7166	0.5333	6.9697	3.18E-12	1.33E-10
ENSBTAG00000009733	2346.9117	3.7151	0.5652	6.5731	4.93E-11	1.75E-09
ENSBTAG00000013371	1654.4407	3.7129	0.7408	5.0120	5.39E-07	8.80E-06
ENSBTAG00000043301	12.7591	3.7051	1.0930	3.3897	7.00E-04	4.86E-03
ENSBTAG00000009354	63.5078	3.6984	0.8580	4.3103	1.63E-05	1.88E-04
ENSBTAG00000029930	92.0097	3.6981	0.7415	4.9876	6.11E-07	9.89E-06
ENSBTAG00000027713	3581.8098	3.6924	0.3468	10.6484	1.77E-26	3.92E-24
ENSBTAG00000013187	35276.8595	3.6917	0.2800	13.1839	1.09E-39	5.05E-37
ENSBTAG00000008283	51.0886	3.6903	1.0331	3.5719	3.54E-04	2.72E-03
ENSBTAG00000019371	18.1157	3.6897	1.1576	3.1874	1.44E-03	8.68E-03
ENSBTAG00000005504	154.1326	3.6868	0.5468	6.7427	1.55E-11	5.94E-10
ENSBTAG00000004767	133.5929	3.6835	0.8178	4.5044	6.66E-06	8.38E-05
ENSBTAG00000019545	29.7074	3.6820	1.0365	3.5524	3.82E-04	2.89E-03
ENSBTAG00000017232	4712.5631	3.6792	0.3973	9.2610	2.03E-20	2.42E-18
ENSBTAG00000009455	13.0012	3.6735	1.0830	3.3919	6.94E-04	4.82E-03
ENSBTAG00000030593	24.2520	3.6713	1.0787	3.4034	6.65E-04	4.65E-03
ENSBTAG00000021009	12.7931	3.6659	1.1036	3.3218	8.95E-04	5.92E-03
ENSBTAG00000020620	47.6000	3.6569	0.9135	4.0030	6.25E-05	6.09E-04
ENSBTAG00000010813	101.7256	3.6528	0.4864	7.5096	5.93E-14	3.17E-12
ENSBTAG00000009215	45.5106	3.6525	1.0645	3.4313	6.01E-04	4.27E-03

ENSBTAG00000019779	47.6257	3.6522	0.6599	5.5345	3.12E-08	6.49E-07
ENSBTAG00000012082	183.0709	3.6516	0.9030	4.0440	5.26E-05	5.21E-04
ENSBTAG00000013165	153.5095	3.6515	0.9194	3.9716	7.14E-05	6.84E-04
ENSBTAG00000013664	297.5152	3.6505	0.6490	5.6252	1.85E-08	4.05E-07
ENSBTAG00000009214	309.4067	3.6443	0.7504	4.8566	1.19E-06	1.82E-05
ENSBTAG00000030974	124.5432	3.6433	1.0023	3.6348	2.78E-04	2.21E-03
ENSBTAG00000022715	26.5796	3.6409	1.0481	3.4739	5.13E-04	3.73E-03
ENSBTAG00000011316	569.1984	3.6383	0.5979	6.0849	1.17E-09	3.18E-08
ENSBTAG00000033107	140.7893	3.6354	0.8682	4.1870	2.83E-05	3.04E-04
ENSBTAG00000008329	153.3538	3.6345	0.9079	4.0033	6.25E-05	6.09E-04
ENSBTAG00000027020	426.6868	3.6338	0.9401	3.8654	1.11E-04	1.01E-03
ENSBTAG00000029828	52.9041	3.6290	0.9844	3.6864	2.27E-04	1.87E-03
ENSBTAG00000003880	939.9037	3.6280	0.6128	5.9206	3.21E-09	8.09E-08
ENSBTAG00000018531	214.6998	3.6269	0.8520	4.2572	2.07E-05	2.31E-04
ENSBTAG00000010408	1196.2184	3.6248	0.4204	8.6219	6.58E-18	5.92E-16
ENSBTAG00000010820	312.0541	3.6243	0.8149	4.4474	8.69E-06	1.07E-04
ENSBTAG00000048249	78.5832	3.6219	1.0926	3.3149	9.17E-04	6.03E-03
ENSBTAG00000006125	312.3905	3.6212	0.7464	4.8513	1.23E-06	1.86E-05
ENSBTAG00000010987	1060.0956	3.6169	0.7579	4.7721	1.82E-06	2.61E-05
ENSBTAG00000001042	471.8177	3.6122	0.7095	5.0914	3.55E-07	6.08E-06
ENSBTAG00000005055	12.5776	3.6094	1.1133	3.2420	1.19E-03	7.44E-03
ENSBTAG00000017060	516.8318	3.6088	0.7516	4.8013	1.58E-06	2.31E-05
ENSBTAG00000020773	68.6677	3.6080	0.6666	5.4126	6.21E-08	1.23E-06
ENSBTAG00000011740	256.7082	3.6053	0.7088	5.0863	3.65E-07	6.22E-06
ENSBTAG00000005615	32.4967	3.5997	1.0384	3.4665	5.27E-04	3.83E-03
ENSBTAG00000007917	1287.9101	3.5936	0.6030	5.9592	2.53E-09	6.52E-08
ENSBTAG00000020394	37.6415	3.5908	0.9061	3.9627	7.41E-05	7.05E-04
ENSBTAG00000005244	416.2687	3.5824	0.7505	4.7736	1.81E-06	2.60E-05
ENSBTAG00000011914	1427.6753	3.5718	0.6726	5.3106	1.09E-07	2.09E-06
ENSBTAG00000006921	26.8153	3.5687	1.0557	3.3805	7.23E-04	5.00E-03
ENSBTAG00000031993	752.1645	3.5638	0.3836	9.2897	1.55E-20	1.95E-18
ENSBTAG00000006223	90.7374	3.5633	0.7394	4.8193	1.44E-06	2.13E-05
ENSBTAG00000011600	387.6995	3.5617	0.7342	4.8510	1.23E-06	1.86E-05
ENSBTAG00000047600	11.2571	3.5583	1.1009	3.2322	1.23E-03	7.66E-03
ENSBTAG00000031287	76.7095	3.5571	0.8631	4.1215	3.76E-05	3.90E-04
ENSBTAG00000007661	361.6172	3.5557	0.8118	4.3802	1.19E-05	1.41E-04
ENSBTAG00000001927	1294.5163	3.5527	0.6869	5.1722	2.31E-07	4.18E-06
ENSBTAG00000034349	45.2253	3.5518	0.9664	3.6755	2.37E-04	1.94E-03
ENSBTAG00000006998	142.0297	3.5511	1.0625	3.3422	8.31E-04	5.60E-03
ENSBTAG00000012815	102.2867	3.5492	1.0060	3.5280	4.19E-04	3.13E-03
ENSBTAG00000008556	31.7508	3.5466	1.0792	3.2864	1.01E-03	6.54E-03
ENSBTAG00000038337	21.5223	3.5452	1.1170	3.1738	1.50E-03	9.03E-03
ENSBTAG00000019470	160.4027	3.5367	0.8400	4.2106	2.55E-05	2.77E-04

ENSBTAG00000008099	12.2877	3.5333	1.1259	3.1382	1.70E-03	9.98E-03
ENSBTAG00000010882	1016.7808	3.5323	0.5006	7.0565	1.71E-12	7.50E-11
ENSBTAG00000015708	47.5730	3.5322	0.9831	3.5931	3.27E-04	2.53E-03
ENSBTAG00000018270	12.8105	3.5258	1.1140	3.1649	1.55E-03	9.25E-03
ENSBTAG00000006208	186.2942	3.5239	1.0052	3.5058	4.55E-04	3.38E-03
ENSBTAG00000026944	28.5275	3.5052	1.0201	3.4359	5.91E-04	4.22E-03
ENSBTAG00000003489	19.8805	3.5020	1.0712	3.2692	1.08E-03	6.89E-03
ENSBTAG00000015978	1214.7347	3.4950	0.6008	5.8169	5.99E-09	1.43E-07
ENSBTAG00000001879	28.7250	3.4934	0.8473	4.1230	3.74E-05	3.88E-04
ENSBTAG00000006676	5845.1703	3.4932	0.8158	4.2822	1.85E-05	2.09E-04
ENSBTAG00000034985	941.7837	3.4604	0.7938	4.3594	1.30E-05	1.55E-04
ENSBTAG00000047605	64.4927	3.4602	1.0531	3.2859	1.02E-03	6.55E-03
ENSBTAG00000007447	3423.0517	3.4579	0.2396	14.4317	3.27E-47	2.94E-44
ENSBTAG00000013860	935.1382	3.4546	0.6737	5.1277	2.93E-07	5.16E-06
ENSBTAG00000016857	25.9127	3.4518	1.0450	3.3031	9.56E-04	6.24E-03
ENSBTAG00000001585	364.9243	3.4498	0.7103	4.8571	1.19E-06	1.82E-05
ENSBTAG00000009639	632.5147	3.4492	0.5067	6.8078	9.91E-12	3.91E-10
ENSBTAG00000038235	18.0376	3.4376	1.0819	3.1775	1.49E-03	8.92E-03
ENSBTAG00000001009	381.7169	3.4333	0.8153	4.2110	2.54E-05	2.77E-04
ENSBTAG00000016683	4101.8910	3.4281	0.6030	5.6854	1.31E-08	2.92E-07
ENSBTAG00000000185	244.2091	3.4261	0.9392	3.6477	2.65E-04	2.12E-03
ENSBTAG00000047939	264.7179	3.4224	0.6186	5.5323	3.16E-08	6.57E-07
ENSBTAG00000013881	132.0613	3.4128	0.9008	3.7886	1.51E-04	1.32E-03
ENSBTAG00000011960	19871.0095	3.4083	0.5052	6.7467	1.51E-11	5.79E-10
ENSBTAG00000019293	34.5205	3.4063	1.0339	3.2947	9.85E-04	6.40E-03
ENSBTAG00000034295	56.1638	3.3927	0.9763	3.4751	5.11E-04	3.72E-03
ENSBTAG00000019686	201.6724	3.3841	0.7088	4.7742	1.80E-06	2.60E-05
ENSBTAG00000018137	459.2744	3.3824	0.7974	4.2416	2.22E-05	2.46E-04
ENSBTAG00000002629	127.4895	3.3797	0.7229	4.6754	2.93E-06	4.03E-05
ENSBTAG00000020884	780.5113	3.3763	0.7402	4.5613	5.08E-06	6.57E-05
ENSBTAG00000001775	20.5618	3.3740	1.0246	3.2930	9.91E-04	6.43E-03
ENSBTAG00000030845	490.3007	3.3732	0.7604	4.4359	9.17E-06	1.12E-04
ENSBTAG00000010244	41.4001	3.3720	0.9238	3.6500	2.62E-04	2.10E-03
ENSBTAG00000019947	2808.9926	3.3421	0.8050	4.1516	3.30E-05	3.49E-04
ENSBTAG00000014628	20.8875	3.3366	1.0436	3.1972	1.39E-03	8.49E-03
ENSBTAG00000015821	40.6129	3.3302	0.8645	3.8521	1.17E-04	1.05E-03
ENSBTAG00000014857	63.2777	3.3267	1.0446	3.1845	1.45E-03	8.75E-03
ENSBTAG00000002215	3554.3251	3.3218	0.8435	3.9379	8.22E-05	7.70E-04
ENSBTAG00000046924	127.9387	3.3164	0.7760	4.2735	1.92E-05	2.16E-04
ENSBTAG00000033727	92.4380	3.3068	0.7951	4.1591	3.20E-05	3.39E-04
ENSBTAG00000021474	41.0548	3.3053	0.9973	3.3142	9.19E-04	6.04E-03
ENSBTAG00000015228	3759.7133	3.3038	0.7716	4.2819	1.85E-05	2.09E-04
ENSBTAG00000013204	20010.6344	3.2921	0.5604	5.8743	4.25E-09	1.04E-07

ENSBTAG00000031430	502.6243	3.2849	0.8288	3.9635	7.39E-05	7.03E-04
ENSBTAG00000017527	76.4183	3.2816	0.8107	4.0479	5.17E-05	5.14E-04
ENSBTAG00000020592	74.5230	3.2778	0.9829	3.3350	8.53E-04	5.70E-03
ENSBTAG00000004104	52.8175	3.2748	0.9204	3.5578	3.74E-04	2.84E-03
ENSBTAG00000013103	5590.7178	3.2712	0.7272	4.4986	6.84E-06	8.60E-05
ENSBTAG00000046307	9415.6654	3.2704	0.2892	11.3087	1.19E-29	3.08E-27
ENSBTAG00000007116	6947.1953	3.2702	0.6375	5.1299	2.90E-07	5.11E-06
ENSBTAG00000021430	30.0068	3.2693	0.9308	3.5123	4.44E-04	3.30E-03
ENSBTAG00000021144	119.3592	3.2681	0.7592	4.3046	1.67E-05	1.93E-04
ENSBTAG00000000109	25.9193	3.2669	0.7731	4.2256	2.38E-05	2.61E-04
ENSBTAG00000006578	32.5263	3.2654	0.9659	3.3806	7.23E-04	5.00E-03
ENSBTAG00000045767	96.7597	3.2344	0.8683	3.7251	1.95E-04	1.64E-03
ENSBTAG00000012774	39.4305	3.2338	0.9518	3.3977	6.80E-04	4.74E-03
ENSBTAG00000014270	43.3335	3.2321	1.0072	3.2089	1.33E-03	8.22E-03
ENSBTAG00000004636	2853.3852	3.2223	0.2674	12.0515	1.90E-33	6.11E-31
ENSBTAG00000011421	191.7185	3.2160	0.6634	4.8478	1.25E-06	1.88E-05
ENSBTAG00000014806	141.7218	3.2131	0.8447	3.8039	1.42E-04	1.26E-03
ENSBTAG00000018011	96.1160	3.2113	0.7662	4.1913	2.77E-05	3.00E-04
ENSBTAG00000013984	20.7560	3.2113	0.9902	3.2430	1.18E-03	7.42E-03
ENSBTAG00000044856	20.0232	3.2106	0.9697	3.3108	9.30E-04	6.12E-03
ENSBTAG00000008507	42.9146	3.2091	0.8991	3.5692	3.58E-04	2.74E-03
ENSBTAG00000046981	216.8090	3.2037	0.7145	4.4840	7.32E-06	9.15E-05
ENSBTAG00000008182	132.9640	3.2031	0.4820	6.6450	3.03E-11	1.11E-09
ENSBTAG00000008462	45.0512	3.2017	0.9393	3.4087	6.53E-04	4.58E-03
ENSBTAG00000047879	16.3702	3.2005	0.9640	3.3202	9.00E-04	5.94E-03
ENSBTAG00000014912	4331.7913	3.1953	0.7852	4.0693	4.72E-05	4.73E-04
ENSBTAG00000012451	186.3283	3.1942	0.8367	3.8178	1.35E-04	1.19E-03
ENSBTAG00000010943	2975.9144	3.1941	0.3253	9.8198	9.25E-23	1.60E-20
ENSBTAG00000006579	268.3175	3.1861	0.8877	3.5893	3.32E-04	2.56E-03
ENSBTAG00000017797	40.9124	3.1834	1.0130	3.1426	1.67E-03	9.87E-03
ENSBTAG00000016354	1117.6477	3.1754	0.5543	5.7283	1.01E-08	2.33E-07
ENSBTAG00000013401	1584.0769	3.1718	0.3799	8.3498	6.84E-17	5.39E-15
ENSBTAG00000019903	108.6073	3.1708	0.9935	3.1915	1.42E-03	8.59E-03
ENSBTAG00000009355	457.5655	3.1676	0.6613	4.7903	1.67E-06	2.43E-05
ENSBTAG00000002416	69.6974	3.1645	0.9617	3.2904	1.00E-03	6.47E-03
ENSBTAG00000002615	193.2870	3.1639	0.5056	6.2571	3.92E-10	1.18E-08
ENSBTAG00000011343	64.0164	3.1625	0.7361	4.2965	1.73E-05	1.98E-04
ENSBTAG00000007176	433.5937	3.1581	0.5266	5.9972	2.01E-09	5.28E-08
ENSBTAG00000040512	128.9096	3.1560	0.8509	3.7089	2.08E-04	1.73E-03
ENSBTAG00000002593	72.4071	3.1527	0.5781	5.4532	4.95E-08	9.94E-07
ENSBTAG00000015666	236.5518	3.1511	0.3810	8.2716	1.32E-16	1.01E-14
ENSBTAG00000009172	28410.2637	3.1500	0.5841	5.3930	6.93E-08	1.37E-06
ENSBTAG00000032084	188.1399	3.1483	0.4730	6.6564	2.81E-11	1.03E-09

ENSBTAG00000024503	836.2579	3.1378	0.7883	3.9806	6.88E-05	6.62E-04
ENSBTAG00000039922	25.0476	3.1368	0.9739	3.2208	1.28E-03	7.92E-03
ENSBTAG00000016116	197.3992	3.1271	0.7001	4.4666	7.95E-06	9.85E-05
ENSBTAG00000016399	819.7429	3.1268	0.5063	6.1753	6.60E-10	1.89E-08
ENSBTAG00000003054	204.4681	3.1057	0.6384	4.8647	1.15E-06	1.76E-05
ENSBTAG00000019967	281.0627	3.1052	0.8756	3.5462	3.91E-04	2.95E-03
ENSBTAG00000047880	2552.3056	3.1002	0.8519	3.6394	2.73E-04	2.18E-03
ENSBTAG00000040244	139.1262	3.0979	0.9010	3.4381	5.86E-04	4.20E-03
ENSBTAG00000046763	7485.8598	3.0975	0.5968	5.1904	2.10E-07	3.80E-06
ENSBTAG00000015742	424.6134	3.0963	0.6730	4.6009	4.21E-06	5.55E-05
ENSBTAG00000009256	1090.0591	3.0958	0.5803	5.3348	9.56E-08	1.86E-06
ENSBTAG00000009845	3518.5512	3.0935	0.4344	7.1218	1.07E-12	4.76E-11
ENSBTAG00000001081	2227.8793	3.0909	0.3494	8.8476	8.94E-19	8.74E-17
ENSBTAG00000039067	47.6863	3.0898	0.9259	3.3371	8.47E-04	5.68E-03
ENSBTAG00000012779	91.0009	3.0893	0.8360	3.6952	2.20E-04	1.82E-03
ENSBTAG00000006792	130.3064	3.0866	0.7892	3.9111	9.19E-05	8.51E-04
ENSBTAG00000003016	39.9470	3.0862	0.8529	3.6187	2.96E-04	2.32E-03
ENSBTAG00000001465	22.2921	3.0855	0.9243	3.3384	8.43E-04	5.66E-03
ENSBTAG00000012444	465.1361	3.0754	0.6151	4.9996	5.74E-07	9.33E-06
ENSBTAG00000015337	75.7758	3.0749	0.8707	3.5316	4.13E-04	3.10E-03
ENSBTAG00000007450	142.8462	3.0730	0.6713	4.5778	4.70E-06	6.13E-05
ENSBTAG00000007338	197.8187	3.0705	0.9555	3.2135	1.31E-03	8.10E-03
ENSBTAG00000000720	110.8743	3.0634	0.5173	5.9219	3.18E-09	8.05E-08
ENSBTAG00000008154	196.6277	3.0608	0.7217	4.2412	2.22E-05	2.46E-04
ENSBTAG00000038428	725.4953	3.0527	0.4116	7.4172	1.20E-13	6.13E-12
ENSBTAG00000003727	547.4684	3.0468	0.5735	5.3124	1.08E-07	2.08E-06
ENSBTAG00000002624	506.4162	3.0441	0.6821	4.4629	8.09E-06	1.00E-04
ENSBTAG00000013166	1358.7153	3.0377	0.5307	5.7240	1.04E-08	2.38E-07
ENSBTAG00000046314	1037.6795	3.0306	0.5304	5.7135	1.11E-08	2.52E-07
ENSBTAG00000039335	79.1746	3.0237	0.8314	3.6367	2.76E-04	2.19E-03
ENSBTAG00000020772	197.5829	3.0209	0.8625	3.5026	4.61E-04	3.40E-03
ENSBTAG00000003721	41.9244	3.0198	0.7914	3.8160	1.36E-04	1.20E-03
ENSBTAG00000001662	933.8721	3.0195	0.4908	6.1523	7.64E-10	2.15E-08
ENSBTAG00000024715	121.3413	3.0180	0.7827	3.8559	1.15E-04	1.04E-03
ENSBTAG00000006984	421.5356	3.0164	0.6852	4.4024	1.07E-05	1.29E-04
ENSBTAG00000004189	1409.3925	3.0035	0.3900	7.7008	1.35E-14	7.79E-13
ENSBTAG00000040361	140.5811	2.9991	0.9556	3.1385	1.70E-03	9.98E-03
ENSBTAG00000007763	650.3078	2.9974	0.5271	5.6870	1.29E-08	2.90E-07
ENSBTAG00000000144	28.4155	2.9968	0.8639	3.4690	5.22E-04	3.80E-03
ENSBTAG00000011007	460.1235	2.9935	0.5452	5.4909	4.00E-08	8.15E-07
ENSBTAG00000011640	9660.9268	2.9904	0.3654	8.1851	2.72E-16	1.97E-14
ENSBTAG00000016315	1625.7506	2.9772	0.6102	4.8787	1.07E-06	1.65E-05
ENSBTAG00000045862	76.9514	2.9769	0.7964	3.7378	1.86E-04	1.57E-03

ENSBTAG00000022622	192.1988	2.9760	0.4114	7.2337	4.70E-13	2.25E-11
ENSBTAG00000005978	123.8618	2.9747	0.8356	3.5601	3.71E-04	2.82E-03
ENSBTAG00000020578	523.1109	2.9741	0.5329	5.5816	2.38E-08	5.06E-07
ENSBTAG00000009343	1298.6493	2.9734	0.2888	10.2953	7.40E-25	1.45E-22
ENSBTAG00000019988	46.4210	2.9667	0.8805	3.3693	7.54E-04	5.18E-03
ENSBTAG00000036367	49.8833	2.9665	0.8599	3.4498	5.61E-04	4.04E-03
ENSBTAG00000020294	465.7080	2.9639	0.9382	3.1592	1.58E-03	9.40E-03
ENSBTAG00000030999	429.3746	2.9621	0.3554	8.3336	7.84E-17	6.15E-15
ENSBTAG00000008612	30.3544	2.9611	0.8804	3.3632	7.71E-04	5.28E-03
ENSBTAG00000016963	30.3153	2.9498	0.7869	3.7486	1.78E-04	1.52E-03
ENSBTAG00000009772	622.0885	2.9399	0.3197	9.1955	3.73E-20	4.38E-18
ENSBTAG00000024928	602.3739	2.9281	0.6016	4.8668	1.13E-06	1.75E-05
ENSBTAG00000001602	35700.6767	2.9278	0.3158	9.2697	1.87E-20	2.29E-18
ENSBTAG00000003711	935.6162	2.9252	0.9131	3.2035	1.36E-03	8.35E-03
ENSBTAG00000003892	207.8539	2.9231	0.5779	5.0578	4.24E-07	7.14E-06
ENSBTAG00000010334	24.5806	2.9222	0.8698	3.3598	7.80E-04	5.34E-03
ENSBTAG00000012823	174.9867	2.9136	0.6188	4.7083	2.50E-06	3.47E-05
ENSBTAG00000008690	72.8032	2.9112	0.8993	3.2373	1.21E-03	7.55E-03
ENSBTAG00000020661	73.7776	2.9047	0.8145	3.5663	3.62E-04	2.76E-03
ENSBTAG00000039151	23.1227	2.9023	0.8662	3.3507	8.06E-04	5.47E-03
ENSBTAG00000016411	1240.9844	2.8983	0.6102	4.7498	2.04E-06	2.89E-05
ENSBTAG00000008953	7348.3702	2.8963	0.2706	10.7025	9.91E-27	2.30E-24
ENSBTAG00000047822	783.2219	2.8907	0.3930	7.3559	1.90E-13	9.37E-12
ENSBTAG00000008573	3183.0775	2.8894	0.5187	5.5699	2.55E-08	5.35E-07
ENSBTAG00000013496	177.8908	2.8881	0.9020	3.2019	1.37E-03	8.38E-03
ENSBTAG00000001826	178.4998	2.8837	0.6976	4.1339	3.57E-05	3.72E-04
ENSBTAG00000002129	44.3921	2.8819	0.7853	3.6697	2.43E-04	1.97E-03
ENSBTAG00000010992	585.3844	2.8784	0.8133	3.5391	4.02E-04	3.02E-03
ENSBTAG00000008886	63.1999	2.8755	0.7830	3.6726	2.40E-04	1.95E-03
ENSBTAG00000023907	1851.7257	2.8732	0.7153	4.0165	5.91E-05	5.80E-04
ENSBTAG00000033345	6893.5307	2.8662	0.7473	3.8353	1.25E-04	1.12E-03
ENSBTAG00000046054	44082.4066	2.8655	0.3282	8.7318	2.51E-18	2.31E-16
ENSBTAG00000003301	67.0954	2.8654	0.5418	5.2891	1.23E-07	2.33E-06
ENSBTAG00000012278	1126.4949	2.8633	0.2767	10.3470	4.32E-25	8.89E-23
ENSBTAG00000046862	2305.1221	2.8599	0.5771	4.9559	7.20E-07	1.14E-05
ENSBTAG00000004423	871.1363	2.8573	0.4590	6.2246	4.83E-10	1.41E-08
ENSBTAG00000046155	35712.4352	2.8548	0.5117	5.5788	2.42E-08	5.12E-07
ENSBTAG00000030932	66.3386	2.8483	0.8772	3.2469	1.17E-03	7.34E-03
ENSBTAG00000009942	4946.9475	2.8475	0.3778	7.5366	4.82E-14	2.60E-12
ENSBTAG00000004641	1211.4609	2.8369	0.3830	7.4063	1.30E-13	6.61E-12
ENSBTAG00000018691	70.9633	2.8271	0.8399	3.3661	7.62E-04	5.24E-03
ENSBTAG00000004662	1268.1307	2.8180	0.5368	5.2496	1.52E-07	2.86E-06
ENSBTAG00000005154	214.5775	2.8141	0.4802	5.8598	4.63E-09	1.13E-07

ENSBTAG00000008103	910.0460	2.8137	0.7113	3.9557	7.63E-05	7.20E-04
ENSBTAG00000026461	4802.1837	2.8135	0.2146	13.1128	2.78E-39	1.21E-36
ENSBTAG00000004777	304.3161	2.8076	0.8248	3.4038	6.65E-04	4.65E-03
ENSBTAG00000010913	180.8343	2.8010	0.6465	4.3328	1.47E-05	1.71E-04
ENSBTAG00000018119	71.3050	2.7998	0.8743	3.2024	1.36E-03	8.37E-03
ENSBTAG00000005668	24150.8023	2.7990	0.5910	4.7358	2.18E-06	3.07E-05
ENSBTAG00000046014	148.0230	2.7968	0.8398	3.3302	8.68E-04	5.78E-03
ENSBTAG00000013029	643.7374	2.7961	0.4162	6.7182	1.84E-11	6.93E-10
ENSBTAG00000011581	113.2325	2.7937	0.7878	3.5462	3.91E-04	2.95E-03
ENSBTAG00000001156	1189.5094	2.7855	0.4057	6.8652	6.64E-12	2.68E-10
ENSBTAG00000007485	621.0263	2.7850	0.5605	4.9687	6.74E-07	1.07E-05
ENSBTAG00000024918	1820.9341	2.7806	0.7645	3.6373	2.76E-04	2.19E-03
ENSBTAG00000017306	157.3633	2.7786	0.7335	3.7883	1.52E-04	1.32E-03
ENSBTAG00000006139	668.0300	2.7784	0.4971	5.5891	2.28E-08	4.88E-07
ENSBTAG00000015913	12647.3024	2.7772	0.3314	8.3811	5.24E-17	4.26E-15
ENSBTAG00000003060	17099.3485	2.7747	0.3487	7.9571	1.76E-15	1.18E-13
ENSBTAG00000020457	156.8908	2.7746	0.8340	3.3267	8.79E-04	5.84E-03
ENSBTAG00000039731	266.9642	2.7743	0.7717	3.5949	3.25E-04	2.52E-03
ENSBTAG00000000163	37441.7714	2.7690	0.5261	5.2634	1.41E-07	2.67E-06
ENSBTAG00000020173	186.3202	2.7640	0.8459	3.2675	1.08E-03	6.91E-03
ENSBTAG00000017763	2247.6335	2.7630	0.3848	7.1796	6.99E-13	3.22E-11
ENSBTAG00000002758	70.6803	2.7562	0.8346	3.3022	9.59E-04	6.26E-03
ENSBTAG00000045588	3949.8034	2.7528	0.8348	3.2973	9.76E-04	6.35E-03
ENSBTAG00000013505	99.4924	2.7523	0.6471	4.2535	2.10E-05	2.35E-04
ENSBTAG00000003836	456.1461	2.7509	0.6675	4.1209	3.77E-05	3.90E-04
ENSBTAG00000005589	2158.9638	2.7453	0.7145	3.8423	1.22E-04	1.09E-03
ENSBTAG00000045685	26.0353	2.7441	0.7565	3.6273	2.86E-04	2.26E-03
ENSBTAG00000008441	807.6836	2.7425	0.4776	5.7421	9.35E-09	2.17E-07
ENSBTAG00000015386	33.4634	2.7364	0.8519	3.2122	1.32E-03	8.14E-03
ENSBTAG00000030334	73.9195	2.7326	0.8514	3.2094	1.33E-03	8.21E-03
ENSBTAG00000001801	9720.1364	2.7316	0.5237	5.2164	1.82E-07	3.36E-06
ENSBTAG00000004273	34.3873	2.7315	0.8065	3.3869	7.07E-04	4.90E-03
ENSBTAG00000001143	353.7619	2.7279	0.7343	3.7153	2.03E-04	1.70E-03
ENSBTAG00000004010	5303.6465	2.7252	0.7003	3.8915	9.96E-05	9.16E-04
ENSBTAG00000039049	41.1595	2.7242	0.7431	3.6660	2.46E-04	2.00E-03
ENSBTAG00000007378	171.3355	2.7180	0.8571	3.1712	1.52E-03	9.09E-03
ENSBTAG00000009257	48.4241	2.7144	0.8599	3.1566	1.60E-03	9.47E-03
ENSBTAG00000012343	2291.0825	2.7132	0.2321	11.6888	1.45E-31	4.17E-29
ENSBTAG00000026919	268.8903	2.7113	0.4586	5.9122	3.38E-09	8.43E-08
ENSBTAG00000018077	204.2096	2.7073	0.4231	6.3985	1.57E-10	5.13E-09
ENSBTAG00000006846	457.2254	2.6991	0.6514	4.1433	3.42E-05	3.59E-04
ENSBTAG00000019652	249.3855	2.6959	0.4341	6.2108	5.27E-10	1.53E-08
ENSBTAG00000019017	2038.5130	2.6950	0.6215	4.3365	1.45E-05	1.69E-04

ENSBTAG00000006568	1147.0549	2.6894	0.5903	4.5558	5.22E-06	6.72E-05
ENSBTAG00000004216	390.7935	2.6878	0.3070	8.7553	2.04E-18	1.91E-16
ENSBTAG00000018255	387.6863	2.6876	0.8155	3.2956	9.82E-04	6.38E-03
ENSBTAG00000020536	536.3200	2.6855	0.7708	3.4839	4.94E-04	3.61E-03
ENSBTAG00000008293	1309.8238	2.6825	0.3942	6.8057	1.01E-11	3.95E-10
ENSBTAG00000043268	23.4523	2.6762	0.7821	3.4216	6.22E-04	4.41E-03
ENSBTAG00000010069	6423.0355	2.6736	0.8367	3.1953	1.40E-03	8.52E-03
ENSBTAG00000015188	1986.3955	2.6677	0.4193	6.3615	2.00E-10	6.40E-09
ENSBTAG00000004564	1556.6328	2.6670	0.2578	10.3462	4.35E-25	8.89E-23
ENSBTAG00000017233	119.2876	2.6668	0.7013	3.8025	1.43E-04	1.26E-03
ENSBTAG00000003889	3047.8704	2.6562	0.2176	12.2055	2.91E-34	9.80E-32
ENSBTAG00000015127	1836.2790	2.6560	0.6347	4.1843	2.86E-05	3.07E-04
ENSBTAG00000012191	2706.9528	2.6478	0.2985	8.8712	7.24E-19	7.23E-17
ENSBTAG00000020520	365.4901	2.6401	0.6429	4.1066	4.01E-05	4.13E-04
ENSBTAG00000001822	381.0149	2.6346	0.6275	4.1985	2.69E-05	2.91E-04
ENSBTAG00000010371	4849.4970	2.6276	0.5211	5.0425	4.59E-07	7.64E-06
ENSBTAG00000043432	24.2152	2.6267	0.7741	3.3935	6.90E-04	4.80E-03
ENSBTAG00000013793	376.5425	2.6254	0.3665	7.1641	7.83E-13	3.58E-11
ENSBTAG00000021837	123.4193	2.6142	0.7316	3.5732	3.53E-04	2.70E-03
ENSBTAG00000007705	135.3153	2.6071	0.5731	4.5492	5.39E-06	6.92E-05
ENSBTAG00000025233	183.8530	2.6062	0.7318	3.5611	3.69E-04	2.81E-03
ENSBTAG00000045896	250.0001	2.5989	0.5247	4.9535	7.29E-07	1.15E-05
ENSBTAG00000012436	61.3316	2.5986	0.8108	3.2049	1.35E-03	8.32E-03
ENSBTAG00000024904	33.5605	2.5970	0.7091	3.6624	2.50E-04	2.02E-03
ENSBTAG00000021505	2485.4385	2.5944	0.2895	8.9614	3.21E-19	3.41E-17
ENSBTAG00000010085	747.3401	2.5928	0.2784	9.3145	1.22E-20	1.60E-18
ENSBTAG00000006017	3879.9494	2.5907	0.3768	6.8751	6.20E-12	2.52E-10
ENSBTAG00000005745	2590.1458	2.5889	0.3336	7.7616	8.39E-15	4.96E-13
ENSBTAG00000006523	1354.0251	2.5877	0.3483	7.4303	1.08E-13	5.58E-12
ENSBTAG00000016494	1455.3213	2.5859	0.4661	5.5476	2.90E-08	6.04E-07
ENSBTAG00000044049	54.7053	2.5798	0.7816	3.3007	9.64E-04	6.29E-03
ENSBTAG00000000396	771.2555	2.5785	0.4548	5.6695	1.43E-08	3.18E-07
ENSBTAG00000014947	14708.3628	2.5743	0.2764	9.3138	1.23E-20	1.60E-18
ENSBTAG00000040128	211.0644	2.5685	0.4446	5.7773	7.59E-09	1.79E-07
ENSBTAG00000014091	4031.3192	2.5632	0.7315	3.5040	4.58E-04	3.39E-03
ENSBTAG00000014826	4320.7882	2.5611	0.4568	5.6066	2.06E-08	4.48E-07
ENSBTAG00000010826	3442.2548	2.5607	0.3639	7.0367	1.97E-12	8.53E-11
ENSBTAG00000021438	1819.1853	2.5588	0.3023	8.4651	2.56E-17	2.12E-15
ENSBTAG00000021568	44.4145	2.5552	0.7770	3.2885	1.01E-03	6.50E-03
ENSBTAG00000021523	7193.4909	2.5543	0.3675	6.9505	3.64E-12	1.50E-10
ENSBTAG00000008053	2628.2221	2.5524	0.3031	8.4201	3.76E-17	3.07E-15
ENSBTAG00000009284	72.0691	2.5500	0.6840	3.7279	1.93E-04	1.63E-03
ENSBTAG00000004468	42.5050	2.5473	0.7652	3.3291	8.71E-04	5.79E-03

ENSBTAG00000037533	7209.0251	2.5467	0.5171	4.9255	8.42E-07	1.32E-05
ENSBTAG00000024648	2202.0628	2.5443	0.4461	5.7035	1.17E-08	2.67E-07
ENSBTAG00000000815	3713.6853	2.5429	0.2957	8.5996	8.00E-18	6.92E-16
ENSBTAG00000011970	624.3854	2.5386	0.5236	4.8486	1.24E-06	1.88E-05
ENSBTAG00000018151	424.2647	2.5383	0.3655	6.9447	3.79E-12	1.56E-10
ENSBTAG00000033961	411.2068	2.5297	0.4515	5.6031	2.11E-08	4.56E-07
ENSBTAG00000031231	1742.5838	2.5274	0.5295	4.7737	1.81E-06	2.60E-05
ENSBTAG00000007514	473.9668	2.5208	0.7466	3.3765	7.34E-04	5.07E-03
ENSBTAG00000014390	647.5517	2.5199	0.5872	4.2912	1.78E-05	2.02E-04
ENSBTAG00000003237	140.1828	2.5182	0.6023	4.1811	2.90E-05	3.11E-04
ENSBTAG00000008585	3314.1926	2.5157	0.5631	4.4673	7.92E-06	9.83E-05
ENSBTAG00000019312	3744.6300	2.5107	0.3056	8.2162	2.10E-16	1.53E-14
ENSBTAG00000003345	137.3549	2.5106	0.5969	4.2058	2.60E-05	2.82E-04
ENSBTAG00000002108	77118.5803	2.5081	0.4721	5.3123	1.08E-07	2.08E-06
ENSBTAG00000015569	107.7328	2.4881	0.5198	4.7863	1.70E-06	2.47E-05
ENSBTAG00000031962	135.9291	2.4851	0.5735	4.3334	1.47E-05	1.71E-04
ENSBTAG00000037907	5402.1314	2.4837	0.5201	4.7754	1.79E-06	2.58E-05
ENSBTAG00000019900	1344.1985	2.4815	0.3885	6.3878	1.68E-10	5.44E-09
ENSBTAG00000022007	302.4522	2.4788	0.5725	4.3297	1.49E-05	1.74E-04
ENSBTAG00000021457	181.5558	2.4698	0.7082	3.4875	4.88E-04	3.58E-03
ENSBTAG00000009654	501.4597	2.4660	0.6358	3.8788	1.05E-04	9.58E-04
ENSBTAG00000011909	2071.3635	2.4635	0.2784	8.8492	8.81E-19	8.67E-17
ENSBTAG00000018043	353.6681	2.4597	0.5396	4.5585	5.15E-06	6.66E-05
ENSBTAG00000005043	3386.5329	2.4584	0.6333	3.8821	1.04E-04	9.46E-04
ENSBTAG00000019059	139.6131	2.4510	0.6593	3.7176	2.01E-04	1.68E-03
ENSBTAG00000015166	46.2610	2.4441	0.6861	3.5624	3.68E-04	2.80E-03
ENSBTAG00000011793	6342.4504	2.4441	0.3639	6.7157	1.87E-11	7.03E-10
ENSBTAG00000004787	365.0731	2.4407	0.6439	3.7905	1.50E-04	1.31E-03
ENSBTAG00000020764	558.3394	2.4395	0.6001	4.0651	4.80E-05	4.81E-04
ENSBTAG00000008809	55.1561	2.4323	0.5760	4.2229	2.41E-05	2.64E-04
ENSBTAG00000044064	1897.4203	2.4298	0.3197	7.5994	2.97E-14	1.63E-12
ENSBTAG00000013706	106.7812	2.4278	0.7671	3.1648	1.55E-03	9.25E-03
ENSBTAG00000012781	21004.3384	2.4174	0.3352	7.2111	5.55E-13	2.65E-11
ENSBTAG00000038794	2809.4131	2.4118	0.4291	5.6210	1.90E-08	4.14E-07
ENSBTAG00000001146	697.0519	2.4098	0.3975	6.0626	1.34E-09	3.64E-08
ENSBTAG00000003120	1270.6775	2.4075	0.3686	6.5308	6.54E-11	2.24E-09
ENSBTAG00000002527	176.9617	2.4052	0.3662	6.5681	5.10E-11	1.79E-09
ENSBTAG00000000446	351.4221	2.4006	0.4351	5.5176	3.44E-08	7.08E-07
ENSBTAG00000015341	827.7085	2.3992	0.5323	4.5076	6.56E-06	8.28E-05
ENSBTAG00000005072	27.2115	2.3922	0.7183	3.3303	8.68E-04	5.78E-03
ENSBTAG00000003375	2236.3283	2.3906	0.5196	4.6011	4.20E-06	5.55E-05
ENSBTAG00000002938	409.1860	2.3894	0.3863	6.1854	6.19E-10	1.78E-08
ENSBTAG00000015543	10924.0468	2.3757	0.4300	5.5253	3.29E-08	6.80E-07

ENSBTAG00000034848	1297.0319	2.3721	0.5519	4.2980	1.72E-05	1.97E-04
ENSBTAG00000014269	99216.9957	2.3645	0.4921	4.8050	1.55E-06	2.28E-05
ENSBTAG00000010171	5187.4675	2.3625	0.2209	10.6932	1.09E-26	2.46E-24
ENSBTAG00000045500	152.8593	2.3519	0.4493	5.2349	1.65E-07	3.08E-06
ENSBTAG00000009239	127.8547	2.3512	0.4994	4.7083	2.50E-06	3.47E-05
ENSBTAG00000007922	81.5115	2.3443	0.4882	4.8016	1.57E-06	2.31E-05
ENSBTAG00000011912	1256.0832	2.3430	0.4028	5.8164	6.01E-09	1.43E-07
ENSBTAG00000038079	1608.6853	2.3342	0.5069	4.6047	4.13E-06	5.47E-05
ENSBTAG00000020330	64907.1012	2.3235	0.3537	6.5683	5.09E-11	1.79E-09
ENSBTAG00000003012	833.4196	2.3160	0.4506	5.1401	2.75E-07	4.85E-06
ENSBTAG00000027655	127.7520	2.3075	0.4631	4.9826	6.27E-07	1.01E-05
ENSBTAG00000010522	278.9767	2.3045	0.7153	3.2218	1.27E-03	7.90E-03
ENSBTAG00000016060	808.9224	2.3019	0.3081	7.4713	7.94E-14	4.18E-12
ENSBTAG00000005744	5232.0558	2.2993	0.3837	5.9918	2.08E-09	5.42E-08
ENSBTAG00000006928	106847.9997	2.2963	0.5909	3.8865	1.02E-04	9.32E-04
ENSBTAG00000046561	1410.6307	2.2955	0.2757	8.3261	8.35E-17	6.51E-15
ENSBTAG00000025554	63.2922	2.2945	0.5900	3.8891	1.01E-04	9.22E-04
ENSBTAG00000005208	1060.0203	2.2840	0.3124	7.3116	2.64E-13	1.29E-11
ENSBTAG00000002804	128.7395	2.2653	0.4437	5.1058	3.29E-07	5.69E-06
ENSBTAG00000009201	379.7499	2.2589	0.5183	4.3582	1.31E-05	1.56E-04
ENSBTAG00000000266	2929.2015	2.2579	0.3066	7.3639	1.79E-13	8.92E-12
ENSBTAG00000013863	1728.7157	2.2530	0.5935	3.7960	1.47E-04	1.29E-03
ENSBTAG00000012107	9573.0485	2.2528	0.4251	5.2995	1.16E-07	2.22E-06
ENSBTAG00000003532	1075.2285	2.2526	0.3996	5.6371	1.73E-08	3.81E-07
ENSBTAG00000010388	60.5397	2.2505	0.7140	3.1521	1.62E-03	9.60E-03
ENSBTAG00000010106	3544.4755	2.2493	0.4015	5.6020	2.12E-08	4.58E-07
ENSBTAG00000004196	763.7178	2.2465	0.6393	3.5142	4.41E-04	3.28E-03
ENSBTAG00000009749	125.3801	2.2387	0.7004	3.1964	1.39E-03	8.50E-03
ENSBTAG00000000222	2475.8133	2.2370	0.3034	7.3733	1.66E-13	8.37E-12
ENSBTAG00000001579	1431.1497	2.2327	0.2811	7.9441	1.96E-15	1.29E-13
ENSBTAG00000007651	433.2489	2.2224	0.4165	5.3361	9.50E-08	1.84E-06
ENSBTAG00000017639	1267.5420	2.2189	0.2837	7.8228	5.17E-15	3.19E-13
ENSBTAG00000004964	474.7365	2.2145	0.2975	7.4446	9.72E-14	5.02E-12
ENSBTAG00000021767	12178.0845	2.2095	0.3124	7.0730	1.52E-12	6.73E-11
ENSBTAG00000024105	50.5060	2.1952	0.6665	3.2936	9.89E-04	6.42E-03
ENSBTAG00000018951	1728.9458	2.1925	0.4745	4.6207	3.83E-06	5.11E-05
ENSBTAG00000010361	8682.5872	2.1886	0.4778	4.5806	4.64E-06	6.06E-05
ENSBTAG00000021818	1523.3348	2.1835	0.3875	5.6344	1.76E-08	3.86E-07
ENSBTAG00000015147	505.9634	2.1831	0.3592	6.0783	1.21E-09	3.31E-08
ENSBTAG00000020281	1933.3779	2.1818	0.4022	5.4250	5.79E-08	1.15E-06
ENSBTAG00000013196	146.7533	2.1794	0.6303	3.4577	5.45E-04	3.94E-03
ENSBTAG00000047077	1937.7514	2.1644	0.3083	7.0212	2.20E-12	9.41E-11
ENSBTAG00000008530	301.0619	2.1620	0.3663	5.9026	3.58E-09	8.85E-08

ENSBTAG00000001141	15343.9565	2.1515	0.3251	6.6173	3.66E-11	1.32E-09
ENSBTAG00000014551	151.6153	2.1462	0.5911	3.6305	2.83E-04	2.24E-03
ENSBTAG00000018157	1952.9350	2.1331	0.4469	4.7729	1.82E-06	2.60E-05
ENSBTAG00000044077	101.9581	2.1319	0.4569	4.6656	3.08E-06	4.19E-05
ENSBTAG00000047648	347.1199	2.1299	0.6593	3.2306	1.24E-03	7.70E-03
ENSBTAG00000017488	888.5496	2.1298	0.3247	6.5584	5.44E-11	1.90E-09
ENSBTAG00000013869	4681.8428	2.1295	0.2711	7.8549	4.00E-15	2.55E-13
ENSBTAG00000011744	1572.1375	2.1293	0.2856	7.4557	8.94E-14	4.67E-12
ENSBTAG00000002836	1304.1169	2.1262	0.4109	5.1748	2.28E-07	4.12E-06
ENSBTAG00000042974	45.2399	2.1250	0.6318	3.3632	7.70E-04	5.28E-03
ENSBTAG00000015413	251.9515	2.1182	0.4400	4.8146	1.47E-06	2.18E-05
ENSBTAG00000017907	826.0190	2.1130	0.3583	5.8979	3.68E-09	9.06E-08
ENSBTAG00000015606	115.9744	2.1110	0.4145	5.0931	3.52E-07	6.04E-06
ENSBTAG00000014650	995.7060	2.1109	0.2735	7.7180	1.18E-14	6.84E-13
ENSBTAG00000014538	94.4638	2.1072	0.6181	3.4093	6.51E-04	4.57E-03
ENSBTAG00000000897	4196.7631	2.1065	0.5072	4.1535	3.27E-05	3.47E-04
ENSBTAG00000010344	780.8833	2.0989	0.5950	3.5277	4.19E-04	3.13E-03
ENSBTAG00000015498	45.7167	2.0979	0.6528	3.2135	1.31E-03	8.10E-03
ENSBTAG00000005997	8587.7135	2.0908	0.4776	4.3777	1.20E-05	1.43E-04
ENSBTAG00000020975	210.5871	2.0903	0.4002	5.2238	1.75E-07	3.25E-06
ENSBTAG00000010376	7355.2078	2.0902	0.2624	7.9655	1.65E-15	1.10E-13
ENSBTAG00000015580	2822.6730	2.0901	0.3541	5.9030	3.57E-09	8.85E-08
ENSBTAG00000012408	191.2258	2.0882	0.5148	4.0561	4.99E-05	4.97E-04
ENSBTAG00000019919	61.8296	2.0876	0.6428	3.2477	1.16E-03	7.32E-03
ENSBTAG00000000053	1717.5346	2.0773	0.3892	5.3371	9.44E-08	1.84E-06
ENSBTAG00000020780	3358.9432	2.0737	0.3993	5.1933	2.07E-07	3.75E-06
ENSBTAG00000003845	281.3724	2.0699	0.4711	4.3942	1.11E-05	1.34E-04
ENSBTAG00000024578	34.2303	2.0586	0.6152	3.3464	8.19E-04	5.53E-03
ENSBTAG00000009428	113.9638	2.0516	0.4911	4.1777	2.94E-05	3.15E-04
ENSBTAG00000014636	1188.1509	2.0328	0.2273	8.9421	3.82E-19	3.93E-17
ENSBTAG00000032905	1819.4580	2.0325	0.3232	6.2894	3.19E-10	9.88E-09
ENSBTAG00000021813	3868.6250	2.0191	0.5344	3.7786	1.58E-04	1.36E-03
ENSBTAG00000046837	15557.6721	2.0157	0.3222	6.2558	3.95E-10	1.18E-08
ENSBTAG00000000877	390.1225	2.0153	0.2918	6.9076	4.93E-12	2.02E-10
ENSBTAG00000025856	2300.3456	2.0137	0.3605	5.5858	2.33E-08	4.95E-07
ENSBTAG00000001294	1943.1496	2.0059	0.4416	4.5425	5.56E-06	7.12E-05
ENSBTAG00000011866	663.8143	1.9977	0.4463	4.4762	7.60E-06	9.46E-05
ENSBTAG00000009952	573.0594	1.9952	0.3371	5.9185	3.25E-09	8.16E-08
ENSBTAG00000017490	12520.2818	1.9945	0.4083	4.8845	1.04E-06	1.61E-05
ENSBTAG00000037804	214.4320	1.9932	0.6078	3.2794	1.04E-03	6.69E-03
ENSBTAG00000011460	439.0249	1.9905	0.4255	4.6786	2.89E-06	3.97E-05
ENSBTAG00000013996	375.6471	1.9892	0.5721	3.4772	5.07E-04	3.70E-03
ENSBTAG00000015457	1009.9585	1.9854	0.4783	4.1506	3.32E-05	3.50E-04

ENSBTAG00000004406	1442.1891	1.9845	0.2326	8.5317	1.44E-17	1.24E-15
ENSBTAG00000007646	131.9339	1.9816	0.5781	3.4278	6.08E-04	4.32E-03
ENSBTAG00000010416	451.4807	1.9813	0.2988	6.6312	3.33E-11	1.21E-09
ENSBTAG00000005523	134.8260	1.9800	0.5397	3.6690	2.43E-04	1.98E-03
ENSBTAG00000032055	3208.9579	1.9790	0.3991	4.9591	7.08E-07	1.12E-05
ENSBTAG00000016406	866.8235	1.9728	0.4732	4.1689	3.06E-05	3.27E-04
ENSBTAG00000009778	677.7437	1.9710	0.2631	7.4907	6.85E-14	3.64E-12
ENSBTAG00000000793	2670.8417	1.9640	0.4063	4.8340	1.34E-06	2.00E-05
ENSBTAG00000015381	18101.5350	1.9627	0.5290	3.7105	2.07E-04	1.72E-03
ENSBTAG00000012577	280.2613	1.9597	0.4804	4.0793	4.52E-05	4.57E-04
ENSBTAG00000018862	147.4427	1.9550	0.5896	3.3158	9.14E-04	6.02E-03
ENSBTAG00000011986	1092.8592	1.9499	0.5830	3.3447	8.24E-04	5.56E-03
ENSBTAG00000030836	1014.8909	1.9470	0.2652	7.3417	2.11E-13	1.04E-11
ENSBTAG00000002688	13318.4043	1.9369	0.3291	5.8854	3.97E-09	9.73E-08
ENSBTAG00000001511	1516.7744	1.9343	0.4747	4.0746	4.61E-05	4.65E-04
ENSBTAG00000015670	52.8482	1.9258	0.6027	3.1955	1.40E-03	8.52E-03
ENSBTAG00000002164	196.1777	1.9230	0.4847	3.9678	7.26E-05	6.92E-04
ENSBTAG00000006593	820.1949	1.9204	0.3080	6.2348	4.52E-10	1.34E-08
ENSBTAG00000000675	2198.3630	1.9171	0.4350	4.4075	1.05E-05	1.26E-04
ENSBTAG00000017457	416.0405	1.9160	0.5195	3.6880	2.26E-04	1.86E-03
ENSBTAG00000018324	124.1713	1.9149	0.4799	3.9905	6.59E-05	6.38E-04
ENSBTAG00000016933	1761.5643	1.9104	0.2661	7.1782	7.06E-13	3.24E-11
ENSBTAG00000002336	300.5272	1.9096	0.4936	3.8690	1.09E-04	9.93E-04
ENSBTAG00000019018	2329.8758	1.9023	0.5821	3.2677	1.08E-03	6.91E-03
ENSBTAG00000003587	551.0854	1.8926	0.2775	6.8209	9.05E-12	3.59E-10
ENSBTAG00000018093	1468.8979	1.8905	0.4632	4.0813	4.48E-05	4.54E-04
ENSBTAG00000007071	736.1500	1.8845	0.5498	3.4279	6.08E-04	4.32E-03
ENSBTAG00000002576	4407.0165	1.8774	0.4588	4.0920	4.28E-05	4.37E-04
ENSBTAG00000018986	4120.8416	1.8762	0.3667	5.1169	3.11E-07	5.41E-06
ENSBTAG00000011204	22481.5836	1.8667	0.5107	3.6552	2.57E-04	2.07E-03
ENSBTAG00000016217	1221.4838	1.8640	0.4867	3.8297	1.28E-04	1.14E-03
ENSBTAG00000011770	632.7575	1.8636	0.3976	4.6875	2.77E-06	3.82E-05
ENSBTAG00000046841	3334.6692	1.8615	0.2879	6.4654	1.01E-10	3.36E-09
ENSBTAG00000011765	11633.9920	1.8608	0.2559	7.2703	3.59E-13	1.73E-11
ENSBTAG00000020125	900.0137	1.8531	0.3680	5.0353	4.77E-07	7.87E-06
ENSBTAG00000046664	1124.0679	1.8515	0.2781	6.6578	2.78E-11	1.02E-09
ENSBTAG00000016529	903.0945	1.8483	0.5605	3.2974	9.76E-04	6.35E-03
ENSBTAG00000008635	535.0088	1.8458	0.5056	3.6505	2.62E-04	2.10E-03
ENSBTAG00000017745	2731.2206	1.8411	0.5019	3.6681	2.44E-04	1.98E-03
ENSBTAG00000004118	4270.2522	1.8392	0.3540	5.1953	2.04E-07	3.71E-06
ENSBTAG00000020272	2880.3367	1.8334	0.4312	4.2520	2.12E-05	2.36E-04
ENSBTAG00000013925	533.8607	1.8295	0.2870	6.3749	1.83E-10	5.89E-09
ENSBTAG00000023629	586.8013	1.8281	0.3788	4.8255	1.40E-06	2.08E-05

ENSBTAG00000013074	6812.4690	1.8279	0.1989	9.1882	3.99E-20	4.60E-18
ENSBTAG00000004291	419.2680	1.8252	0.3800	4.8034	1.56E-06	2.29E-05
ENSBTAG00000000505	1644.7991	1.8141	0.3988	4.5486	5.40E-06	6.93E-05
ENSBTAG00000019530	123.0538	1.8090	0.5327	3.3960	6.84E-04	4.76E-03
ENSBTAG00000014215	1541.7137	1.7943	0.3474	5.1643	2.41E-07	4.34E-06
ENSBTAG00000000040	672.6958	1.7924	0.2833	6.3265	2.51E-10	7.90E-09
ENSBTAG00000011933	2379.9773	1.7889	0.4017	4.4535	8.45E-06	1.04E-04
ENSBTAG00000008401	1158.5253	1.7883	0.5377	3.3258	8.82E-04	5.84E-03
ENSBTAG00000048062	1924.5580	1.7866	0.3684	4.8497	1.24E-06	1.87E-05
ENSBTAG00000018569	3867.4229	1.7832	0.2969	6.0058	1.90E-09	5.04E-08
ENSBTAG00000004659	1226.0283	1.7797	0.4492	3.9622	7.43E-05	7.06E-04
ENSBTAG00000006785	902.8090	1.7760	0.4237	4.1912	2.78E-05	3.00E-04
ENSBTAG00000012170	4950.3846	1.7736	0.2141	8.2837	1.19E-16	9.15E-15
ENSBTAG00000005025	2324.5007	1.7717	0.1996	8.8762	6.92E-19	6.96E-17
ENSBTAG00000004154	602.6215	1.7707	0.5278	3.3549	7.94E-04	5.41E-03
ENSBTAG00000045702	526.2821	1.7698	0.4450	3.9766	6.99E-05	6.72E-04
ENSBTAG00000015195	18418.0816	1.7668	0.4357	4.0556	5.00E-05	4.98E-04
ENSBTAG00000023718	2322.1586	1.7649	0.4665	3.7835	1.55E-04	1.34E-03
ENSBTAG00000013555	1163.1568	1.7647	0.3466	5.0914	3.55E-07	6.08E-06
ENSBTAG00000046971	7051.8077	1.7645	0.3670	4.8084	1.52E-06	2.24E-05
ENSBTAG00000014076	1669.3965	1.7600	0.2411	7.2999	2.88E-13	1.40E-11
ENSBTAG00000021103	15278.1700	1.7573	0.3286	5.3476	8.91E-08	1.74E-06
ENSBTAG00000009887	6473.4722	1.7563	0.4014	4.3751	1.21E-05	1.44E-04
ENSBTAG00000015969	525.2248	1.7560	0.3086	5.6903	1.27E-08	2.85E-07
ENSBTAG00000009112	1356.2842	1.7534	0.5149	3.4054	6.61E-04	4.63E-03
ENSBTAG00000007320	5177.9674	1.7529	0.3184	5.5058	3.68E-08	7.52E-07
ENSBTAG00000010546	656.4487	1.7509	0.4298	4.0739	4.62E-05	4.65E-04
ENSBTAG00000045822	4788.8394	1.7460	0.4395	3.9723	7.12E-05	6.82E-04
ENSBTAG00000008169	255.2346	1.7394	0.4624	3.7614	1.69E-04	1.45E-03
ENSBTAG00000027064	4742.4804	1.7287	0.4626	3.7369	1.86E-04	1.58E-03
ENSBTAG00000019456	413.1778	1.7275	0.2809	6.1500	7.75E-10	2.18E-08
ENSBTAG00000003960	630.1053	1.7270	0.3446	5.0110	5.41E-07	8.84E-06
ENSBTAG00000016751	11975.8988	1.7238	0.2910	5.9229	3.16E-09	8.01E-08
ENSBTAG00000020996	6402.5088	1.7224	0.2623	6.5667	5.15E-11	1.81E-09
ENSBTAG00000015649	790.0651	1.7215	0.3925	4.3863	1.15E-05	1.38E-04
ENSBTAG00000009471	645.1553	1.7206	0.5482	3.1384	1.70E-03	9.98E-03
ENSBTAG00000008323	166.1424	1.7161	0.4000	4.2904	1.78E-05	2.02E-04
ENSBTAG00000013577	391.0146	1.7116	0.4473	3.8265	1.30E-04	1.16E-03
ENSBTAG00000013063	7790.4638	1.7087	0.4515	3.7843	1.54E-04	1.34E-03
ENSBTAG00000000928	295.5750	1.6985	0.3021	5.6221	1.89E-08	4.12E-07
ENSBTAG00000016649	20759.9431	1.6963	0.4275	3.9678	7.25E-05	6.92E-04
ENSBTAG00000019414	851.4643	1.6955	0.5362	3.1620	1.57E-03	9.32E-03
ENSBTAG00000045886	163.3105	1.6940	0.4797	3.5314	4.13E-04	3.10E-03

ENSBTAG00000005833	1422.5122	1.6933	0.3023	5.6018	2.12E-08	4.58E-07
ENSBTAG00000046503	1846.7718	1.6895	0.2493	6.7766	1.23E-11	4.79E-10
ENSBTAG00000009138	437.9660	1.6885	0.3890	4.3411	1.42E-05	1.67E-04
ENSBTAG00000018334	227.5634	1.6856	0.3236	5.2093	1.90E-07	3.48E-06
ENSBTAG00000012735	124.9672	1.6784	0.4166	4.0286	5.61E-05	5.54E-04
ENSBTAG00000011772	1126.8904	1.6691	0.4026	4.1454	3.39E-05	3.57E-04
ENSBTAG00000003290	257.2887	1.6603	0.5284	3.1420	1.68E-03	9.88E-03
ENSBTAG00000008330	692.8459	1.6595	0.4693	3.5360	4.06E-04	3.05E-03
ENSBTAG00000039764	532.1311	1.6534	0.4357	3.7945	1.48E-04	1.29E-03
ENSBTAG00000010785	246.1412	1.6481	0.3398	4.8509	1.23E-06	1.86E-05
ENSBTAG00000003895	370.7871	1.6480	0.4528	3.6398	2.73E-04	2.17E-03
ENSBTAG00000005286	1991.7899	1.6422	0.3588	4.5775	4.70E-06	6.13E-05
ENSBTAG00000003039	908.5325	1.6383	0.4533	3.6144	3.01E-04	2.36E-03
ENSBTAG00000001886	400.2091	1.6369	0.4524	3.6179	2.97E-04	2.33E-03
ENSBTAG00000020454	755.9410	1.6357	0.3925	4.1669	3.09E-05	3.29E-04
ENSBTAG00000001117	1398.3916	1.6309	0.4141	3.9383	8.20E-05	7.69E-04
ENSBTAG00000013524	788.8430	1.6294	0.3185	5.1155	3.13E-07	5.44E-06
ENSBTAG00000018704	2102.8381	1.6249	0.5146	3.1573	1.59E-03	9.45E-03
ENSBTAG00000002329	654.4086	1.6234	0.3859	4.2072	2.59E-05	2.81E-04
ENSBTAG00000031829	1344.1315	1.6193	0.4394	3.6851	2.29E-04	1.88E-03
ENSBTAG00000014366	16039.5282	1.6190	0.4339	3.7311	1.91E-04	1.61E-03
ENSBTAG00000021209	12478.1271	1.6168	0.2793	5.7895	7.06E-09	1.67E-07
ENSBTAG00000012833	1076.4058	1.6155	0.2947	5.4821	4.20E-08	8.55E-07
ENSBTAG00000003295	420.1306	1.6128	0.4700	3.4318	6.00E-04	4.27E-03
ENSBTAG00000011994	2222.4870	1.6085	0.3179	5.0604	4.18E-07	7.06E-06
ENSBTAG00000021011	217.2226	1.6061	0.3500	4.5883	4.47E-06	5.87E-05
ENSBTAG00000008954	1880.6148	1.6056	0.4807	3.3401	8.37E-04	5.64E-03
ENSBTAG00000011880	596.1697	1.6022	0.4036	3.9700	7.19E-05	6.88E-04
ENSBTAG00000015113	1704.0133	1.5999	0.4418	3.6210	2.93E-04	2.30E-03
ENSBTAG00000019839	4011.0448	1.5966	0.3969	4.0223	5.76E-05	5.67E-04
ENSBTAG00000006193	887.5192	1.5932	0.4828	3.3003	9.66E-04	6.29E-03
ENSBTAG00000020616	1753.2565	1.5930	0.3093	5.1500	2.60E-07	4.65E-06
ENSBTAG00000004688	57341.4411	1.5929	0.3180	5.0086	5.48E-07	8.93E-06
ENSBTAG00000012694	204.6281	1.5901	0.4317	3.6829	2.31E-04	1.89E-03
ENSBTAG00000018937	2628.0553	1.5841	0.2683	5.9042	3.54E-09	8.80E-08
ENSBTAG00000001576	95.9240	1.5823	0.4901	3.2284	1.24E-03	7.75E-03
ENSBTAG00000003928	914.9318	1.5789	0.4292	3.6785	2.35E-04	1.92E-03
ENSBTAG00000021342	874.3578	1.5788	0.2705	5.8361	5.34E-09	1.28E-07
ENSBTAG00000010395	1152.6135	1.5781	0.3068	5.1432	2.70E-07	4.79E-06
ENSBTAG00000010906	1414.9836	1.5769	0.2509	6.2851	3.28E-10	1.01E-08
ENSBTAG00000020084	596.0426	1.5759	0.3017	5.2237	1.75E-07	3.25E-06
ENSBTAG00000044065	162.1335	1.5696	0.4674	3.3578	7.86E-04	5.37E-03
ENSBTAG00000017096	529.1194	1.5665	0.4127	3.7959	1.47E-04	1.29E-03

ENSBTAG00000003081	4145.2220	1.5658	0.2850	5.4938	3.93E-08	8.03E-07
ENSBTAG00000004881	600.2126	1.5629	0.3101	5.0407	4.64E-07	7.70E-06
ENSBTAG00000016600	199.7873	1.5621	0.4568	3.4199	6.26E-04	4.43E-03
ENSBTAG00000014021	254.0895	1.5618	0.4248	3.6764	2.37E-04	1.93E-03
ENSBTAG00000016206	3698.6734	1.5604	0.3205	4.8684	1.13E-06	1.73E-05
ENSBTAG00000019479	781.5722	1.5516	0.3285	4.7238	2.32E-06	3.24E-05
ENSBTAG00000010928	409.4299	1.5498	0.3739	4.1446	3.40E-05	3.58E-04
ENSBTAG00000008091	81.9241	1.5478	0.4691	3.2995	9.69E-04	6.31E-03
ENSBTAG00000012575	1734.3490	1.5438	0.2743	5.6291	1.81E-08	3.97E-07
ENSBTAG00000012450	1331.1655	1.5413	0.3703	4.1623	3.15E-05	3.35E-04
ENSBTAG00000010549	775.3812	1.5407	0.4467	3.4492	5.62E-04	4.05E-03
ENSBTAG00000000683	750.3654	1.5385	0.3674	4.1880	2.81E-05	3.03E-04
ENSBTAG00000009708	311.5095	1.5328	0.2748	5.5780	2.43E-08	5.13E-07
ENSBTAG00000019209	1333.8029	1.5299	0.4334	3.5297	4.16E-04	3.11E-03
ENSBTAG00000026309	397.9421	1.5266	0.3434	4.4460	8.75E-06	1.07E-04
ENSBTAG00000017560	437.4656	1.5249	0.3263	4.6731	2.97E-06	4.07E-05
ENSBTAG00000018718	145.9262	1.5229	0.4362	3.4913	4.81E-04	3.53E-03
ENSBTAG00000018160	103838.4761	1.5216	0.3476	4.3770	1.20E-05	1.43E-04
ENSBTAG00000020266	105.8249	1.5213	0.4562	3.3350	8.53E-04	5.70E-03
ENSBTAG00000002970	4927.3491	1.5211	0.3939	3.8617	1.13E-04	1.02E-03
ENSBTAG00000009886	7431.0177	1.5154	0.4050	3.7420	1.83E-04	1.55E-03
ENSBTAG00000021187	12625.5463	1.5150	0.4748	3.1905	1.42E-03	8.61E-03
ENSBTAG00000009502	360.1521	1.5107	0.3880	3.8936	9.87E-05	9.08E-04
ENSBTAG00000019187	2747.3654	1.5072	0.3098	4.8647	1.15E-06	1.76E-05
ENSBTAG00000012273	1757.8867	1.5028	0.3924	3.8301	1.28E-04	1.14E-03
ENSBTAG00000014020	191.2000	1.5018	0.3624	4.1436	3.42E-05	3.59E-04
ENSBTAG00000010803	251.0482	1.4998	0.3542	4.2340	2.30E-05	2.52E-04
ENSBTAG00000009664	351.4431	1.4976	0.4566	3.2796	1.04E-03	6.69E-03
ENSBTAG00000022808	1090.0212	1.4960	0.3161	4.7329	2.21E-06	3.11E-05
ENSBTAG00000014426	4030.2694	1.4937	0.2376	6.2880	3.22E-10	9.94E-09
ENSBTAG00000009482	2324.2180	1.4929	0.2945	5.0688	4.00E-07	6.78E-06
ENSBTAG00000013544	760.3654	1.4921	0.2701	5.5240	3.31E-08	6.84E-07
ENSBTAG00000001142	938.5489	1.4912	0.2488	5.9933	2.06E-09	5.38E-08
ENSBTAG00000008129	351.1641	1.4891	0.4144	3.5937	3.26E-04	2.52E-03
ENSBTAG00000047739	31580.6764	1.4877	0.3946	3.7697	1.63E-04	1.41E-03
ENSBTAG00000021467	531.3820	1.4855	0.3265	4.5504	5.35E-06	6.88E-05
ENSBTAG00000015936	373.1904	1.4822	0.4679	3.1680	1.54E-03	9.17E-03
ENSBTAG00000046549	1149.2300	1.4755	0.2408	6.1263	9.00E-10	2.50E-08
ENSBTAG00000008732	169.1853	1.4733	0.4506	3.2693	1.08E-03	6.89E-03
ENSBTAG00000012314	30017.3816	1.4725	0.3123	4.7154	2.41E-06	3.37E-05
ENSBTAG00000004977	285.3915	1.4700	0.3528	4.1669	3.09E-05	3.29E-04
ENSBTAG00000019219	3617.6295	1.4628	0.2170	6.7400	1.58E-11	6.03E-10
ENSBTAG00000015226	1013.3967	1.4591	0.3927	3.7152	2.03E-04	1.70E-03

ENSBTAG00000000332	708.5445	1.4585	0.3213	4.5392	5.65E-06	7.22E-05
ENSBTAG00000019116	2311.3031	1.4563	0.2131	6.8336	8.28E-12	3.31E-10
ENSBTAG00000010863	3078.8282	1.4559	0.2359	6.1708	6.80E-10	1.93E-08
ENSBTAG00000006654	1780.7979	1.4547	0.3721	3.9095	9.25E-05	8.56E-04
ENSBTAG00000019604	1624.2449	1.4498	0.4507	3.2167	1.30E-03	8.03E-03
ENSBTAG00000011541	861.8666	1.4448	0.4315	3.3485	8.13E-04	5.50E-03
ENSBTAG00000014522	990.2143	1.4385	0.3146	4.5719	4.83E-06	6.28E-05
ENSBTAG00000000363	1078.0847	1.4374	0.3316	4.3343	1.46E-05	1.71E-04
ENSBTAG00000012112	1042.5283	1.4374	0.3475	4.1370	3.52E-05	3.68E-04
ENSBTAG00000005960	5845.8660	1.4336	0.2691	5.3284	9.91E-08	1.92E-06
ENSBTAG00000016271	3763.7557	1.4318	0.3468	4.1286	3.65E-05	3.79E-04
ENSBTAG00000021894	1931.5747	1.4289	0.2838	5.0346	4.79E-07	7.89E-06
ENSBTAG00000017799	2961.9305	1.4264	0.2556	5.5796	2.41E-08	5.10E-07
ENSBTAG00000045980	2059.4593	1.4182	0.3410	4.1589	3.20E-05	3.39E-04
ENSBTAG00000001634	3539.2437	1.4175	0.3213	4.4121	1.02E-05	1.24E-04
ENSBTAG00000015802	3343.0982	1.4149	0.3359	4.2125	2.53E-05	2.75E-04
ENSBTAG00000002287	1906.8353	1.4105	0.3732	3.7791	1.57E-04	1.36E-03
ENSBTAG00000035907	6946.2285	1.4102	0.2297	6.1384	8.33E-10	2.32E-08
ENSBTAG00000016415	2870.4687	1.4036	0.3721	3.7723	1.62E-04	1.39E-03
ENSBTAG00000003401	10964.2503	1.4016	0.2657	5.2742	1.33E-07	2.52E-06
ENSBTAG00000031861	4326.1638	1.3996	0.2994	4.6746	2.94E-06	4.04E-05
ENSBTAG00000006124	1094.1809	1.3984	0.2810	4.9768	6.47E-07	1.04E-05
ENSBTAG00000015212	986.8776	1.3979	0.3732	3.7462	1.80E-04	1.53E-03
ENSBTAG00000020192	5638.0586	1.3977	0.2468	5.6637	1.48E-08	3.27E-07
ENSBTAG00000040585	101.0975	1.3937	0.4068	3.4263	6.12E-04	4.34E-03
ENSBTAG00000006933	3994.3107	1.3928	0.2905	4.7948	1.63E-06	2.38E-05
ENSBTAG00000020469	136.5722	1.3912	0.4002	3.4764	5.08E-04	3.70E-03
ENSBTAG00000017582	295.5784	1.3884	0.3985	3.4841	4.94E-04	3.61E-03
ENSBTAG00000017764	529.0649	1.3881	0.2695	5.1505	2.60E-07	4.64E-06
ENSBTAG00000017626	820.1243	1.3851	0.2474	5.5994	2.15E-08	4.61E-07
ENSBTAG00000047718	100.1063	1.3845	0.4030	3.4353	5.92E-04	4.22E-03
ENSBTAG00000017542	653.1159	1.3753	0.2787	4.9340	8.06E-07	1.27E-05
ENSBTAG00000026233	134.2409	1.3689	0.4191	3.2662	1.09E-03	6.93E-03
ENSBTAG00000001660	859.7483	1.3674	0.2257	6.0591	1.37E-09	3.71E-08
ENSBTAG00000007543	451.8706	1.3384	0.3170	4.2219	2.42E-05	2.65E-04
ENSBTAG00000007427	2297.0026	1.3380	0.1949	6.8644	6.68E-12	2.69E-10
ENSBTAG00000046255	983.9080	1.3356	0.2777	4.8090	1.52E-06	2.24E-05
ENSBTAG00000021020	1888.7092	1.3327	0.3576	3.7272	1.94E-04	1.63E-03
ENSBTAG00000000937	1669.1229	1.3321	0.2675	4.9797	6.37E-07	1.02E-05
ENSBTAG00000001440	2796.0451	1.3316	0.2883	4.6187	3.86E-06	5.15E-05
ENSBTAG00000046533	282.7832	1.3310	0.3523	3.7777	1.58E-04	1.37E-03
ENSBTAG00000002292	26251.1710	1.3250	0.2209	5.9984	1.99E-09	5.26E-08
ENSBTAG00000039296	162.4786	1.3239	0.4169	3.1756	1.50E-03	8.98E-03

ENSBTAG00000046840	1047.9043	1.3208	0.3668	3.6009	3.17E-04	2.46E-03
ENSBTAG00000023933	4565.0385	1.3155	0.2098	6.2691	3.63E-10	1.11E-08
ENSBTAG00000018694	10875.2784	1.3151	0.3819	3.4436	5.74E-04	4.12E-03
ENSBTAG00000006511	402.5110	1.3142	0.3018	4.3551	1.33E-05	1.57E-04
ENSBTAG00000002176	10650.8647	1.3135	0.3412	3.8496	1.18E-04	1.06E-03
ENSBTAG00000004901	731.4075	1.3070	0.4090	3.1960	1.39E-03	8.51E-03
ENSBTAG00000010763	612.9825	1.3068	0.2945	4.4369	9.13E-06	1.12E-04
ENSBTAG00000012589	11023.7504	1.3058	0.2513	5.1962	2.03E-07	3.70E-06
ENSBTAG00000009704	561.1257	1.3040	0.3327	3.9193	8.88E-05	8.26E-04
ENSBTAG00000047027	888.0770	1.3037	0.3312	3.9357	8.29E-05	7.75E-04
ENSBTAG00000047706	1382.8291	1.3028	0.3822	3.4091	6.52E-04	4.58E-03
ENSBTAG00000010353	1127.1353	1.2981	0.3363	3.8601	1.13E-04	1.02E-03
ENSBTAG00000020166	1424.0886	1.2975	0.2788	4.6545	3.25E-06	4.40E-05
ENSBTAG00000016076	354.3457	1.2933	0.3354	3.8558	1.15E-04	1.04E-03
ENSBTAG00000001749	1445.8569	1.2828	0.3766	3.4067	6.58E-04	4.61E-03
ENSBTAG00000008649	373.5299	1.2818	0.3413	3.7553	1.73E-04	1.48E-03
ENSBTAG00000010149	338.9375	1.2810	0.3891	3.2926	9.93E-04	6.44E-03
ENSBTAG00000019804	2896.2646	1.2802	0.3511	3.6461	2.66E-04	2.13E-03
ENSBTAG00000001483	781.6096	1.2759	0.3916	3.2577	1.12E-03	7.10E-03
ENSBTAG00000045904	1587.0267	1.2746	0.4058	3.1406	1.69E-03	9.93E-03
ENSBTAG00000014927	830.8283	1.2657	0.2788	4.5403	5.62E-06	7.19E-05
ENSBTAG00000009213	3078.5686	1.2651	0.2776	4.5576	5.17E-06	6.68E-05
ENSBTAG00000003325	338.6473	1.2643	0.3503	3.6092	3.07E-04	2.40E-03
ENSBTAG00000007634	1159.9481	1.2548	0.2747	4.5673	4.94E-06	6.42E-05
ENSBTAG00000023867	1100.8600	1.2531	0.2393	5.2354	1.65E-07	3.08E-06
ENSBTAG00000002708	1061.9404	1.2474	0.3045	4.0964	4.20E-05	4.29E-04
ENSBTAG00000010547	494.1077	1.2473	0.3899	3.1989	1.38E-03	8.46E-03
ENSBTAG00000014261	4126.4892	1.2468	0.2875	4.3373	1.44E-05	1.69E-04
ENSBTAG00000046724	492.8690	1.2465	0.3554	3.5069	4.53E-04	3.36E-03
ENSBTAG00000005278	1022.0193	1.2424	0.3802	3.2679	1.08E-03	6.91E-03
ENSBTAG00000033077	1164.5001	1.2405	0.2530	4.9028	9.45E-07	1.47E-05
ENSBTAG00000011287	280.9415	1.2380	0.3183	3.8895	1.00E-04	9.21E-04
ENSBTAG00000024633	238.3799	1.2374	0.3943	3.1381	1.70E-03	9.98E-03
ENSBTAG00000026851	1360.0102	1.2325	0.3860	3.1930	1.41E-03	8.56E-03
ENSBTAG00000012425	233.0051	1.2233	0.3887	3.1467	1.65E-03	9.75E-03
ENSBTAG00000003916	1371.9761	1.2212	0.3397	3.5945	3.25E-04	2.52E-03
ENSBTAG00000001856	4839.0509	1.2205	0.2727	4.4757	7.62E-06	9.47E-05
ENSBTAG00000002048	3231.1553	1.2203	0.2862	4.2642	2.01E-05	2.25E-04
ENSBTAG00000047715	1935.4140	1.2195	0.3118	3.9112	9.18E-05	8.51E-04
ENSBTAG00000032083	295.5493	1.2181	0.2815	4.3269	1.51E-05	1.76E-04
ENSBTAG00000003282	245.6628	1.2150	0.3085	3.9390	8.18E-05	7.68E-04
ENSBTAG00000016908	711.5222	1.2145	0.3095	3.9240	8.71E-05	8.11E-04
ENSBTAG00000000356	493.5612	1.2101	0.3252	3.7207	1.99E-04	1.66E-03

ENSBTAG00000021819	2349.3057	1.2068	0.3672	3.2862	1.02E-03	6.54E-03
ENSBTAG00000017597	475.0578	1.2047	0.3571	3.3739	7.41E-04	5.11E-03
ENSBTAG00000009552	5924.4219	1.2041	0.3713	3.2432	1.18E-03	7.42E-03
ENSBTAG00000010206	3368.9013	1.2039	0.3668	3.2817	1.03E-03	6.64E-03
ENSBTAG00000009785	2637.2067	1.2024	0.2737	4.3929	1.12E-05	1.34E-04
ENSBTAG00000015384	1137.5230	1.1992	0.2814	4.2617	2.03E-05	2.27E-04
ENSBTAG00000005865	2143.1183	1.1855	0.2132	5.5603	2.69E-08	5.64E-07
ENSBTAG00000030587	3312.5074	1.1853	0.3255	3.6419	2.71E-04	2.16E-03
ENSBTAG00000003062	752.2512	1.1839	0.3364	3.5187	4.34E-04	3.23E-03
ENSBTAG00000039662	377.3711	1.1825	0.3614	3.2717	1.07E-03	6.84E-03
ENSBTAG00000010899	53517.4797	1.1783	0.3605	3.2688	1.08E-03	6.90E-03
ENSBTAG00000017694	214.2627	1.1745	0.3688	3.1850	1.45E-03	8.74E-03
ENSBTAG00000020030	10118.9515	1.1737	0.3363	3.4906	4.82E-04	3.54E-03
ENSBTAG00000016619	483.5020	1.1728	0.3233	3.6274	2.86E-04	2.26E-03
ENSBTAG00000000744	1674.8921	1.1698	0.3230	3.6219	2.92E-04	2.30E-03
ENSBTAG00000005826	225.8041	1.1615	0.3326	3.4918	4.80E-04	3.53E-03
ENSBTAG00000011597	1692.9017	1.1595	0.2873	4.0365	5.43E-05	5.36E-04
ENSBTAG00000006002	1291.9001	1.1573	0.2803	4.1289	3.65E-05	3.79E-04
ENSBTAG00000021582	2608.5169	1.1512	0.2528	4.5546	5.25E-06	6.75E-05
ENSBTAG00000020681	1100.4932	1.1493	0.3478	3.3045	9.52E-04	6.22E-03
ENSBTAG00000019938	648.1729	1.1479	0.3483	3.2955	9.82E-04	6.38E-03
ENSBTAG00000021008	3024.1820	1.1435	0.3434	3.3299	8.69E-04	5.78E-03
ENSBTAG00000000108	1288.0230	1.1418	0.3409	3.3493	8.10E-04	5.49E-03
ENSBTAG00000012999	533.3731	1.1405	0.3431	3.3237	8.88E-04	5.88E-03
ENSBTAG00000000786	726.8882	1.1326	0.3074	3.6850	2.29E-04	1.88E-03
ENSBTAG00000013138	604.4065	1.1293	0.3247	3.4783	5.05E-04	3.68E-03
ENSBTAG00000014884	2906.5403	1.1271	0.2333	4.8305	1.36E-06	2.03E-05
ENSBTAG00000003264	2635.4317	1.1255	0.2826	3.9826	6.82E-05	6.57E-04
ENSBTAG00000017211	444.0645	1.1215	0.3214	3.4892	4.85E-04	3.56E-03
ENSBTAG00000006616	836.9563	1.1213	0.3221	3.4810	5.00E-04	3.65E-03
ENSBTAG00000032674	718.3819	1.1203	0.3025	3.7038	2.12E-04	1.76E-03
ENSBTAG00000017958	647.3346	1.1198	0.2445	4.5797	4.66E-06	6.08E-05
ENSBTAG00000026716	2879.8581	1.1195	0.2265	4.9428	7.70E-07	1.21E-05
ENSBTAG00000002175	1765.2766	1.1193	0.3040	3.6820	2.31E-04	1.90E-03
ENSBTAG00000016104	1662.8401	1.1185	0.2748	4.0695	4.71E-05	4.73E-04
ENSBTAG00000004497	1291.4429	1.1171	0.2297	4.8630	1.16E-06	1.77E-05
ENSBTAG00000020332	2782.0521	1.1148	0.3054	3.6499	2.62E-04	2.10E-03
ENSBTAG00000020417	2053.2235	1.1142	0.2678	4.1606	3.17E-05	3.37E-04
ENSBTAG00000009267	703.8997	1.1140	0.3297	3.3783	7.29E-04	5.04E-03
ENSBTAG00000018383	241.5042	1.1130	0.3148	3.5355	4.07E-04	3.05E-03
ENSBTAG00000019202	1233.6131	1.1114	0.2500	4.4463	8.74E-06	1.07E-04
ENSBTAG00000013699	3776.8663	1.1112	0.2867	3.8762	1.06E-04	9.67E-04
ENSBTAG00000010178	2427.4077	1.1096	0.2982	3.7208	1.99E-04	1.66E-03

ENSBTAG00000009415	526.1019	1.1090	0.3040	3.6477	2.65E-04	2.12E-03
ENSBTAG00000021980	382.9260	1.1085	0.3257	3.4032	6.66E-04	4.65E-03
ENSBTAG00000005847	1405.5212	1.1078	0.3320	3.3361	8.50E-04	5.69E-03
ENSBTAG00000013612	814.6898	1.1059	0.3198	3.4585	5.43E-04	3.93E-03
ENSBTAG00000021134	2013.5382	1.1055	0.3055	3.6190	2.96E-04	2.32E-03
ENSBTAG00000003399	17297.4869	1.1037	0.2314	4.7703	1.84E-06	2.63E-05
ENSBTAG00000001518	1628.0862	1.1037	0.2473	4.4631	8.08E-06	1.00E-04
ENSBTAG00000031609	1361.1497	1.1032	0.2564	4.3031	1.68E-05	1.93E-04
ENSBTAG00000025516	1653.5239	1.0979	0.3012	3.6451	2.67E-04	2.14E-03
ENSBTAG00000000184	3636.4448	1.0936	0.1965	5.5657	2.61E-08	5.48E-07
ENSBTAG00000046545	1519.5089	1.0920	0.2453	4.4516	8.52E-06	1.05E-04
ENSBTAG00000033170	236.3885	1.0906	0.3437	3.1733	1.51E-03	9.03E-03
ENSBTAG00000003708	5229.9581	1.0878	0.3261	3.3359	8.50E-04	5.69E-03
ENSBTAG00000019538	2770.7396	1.0854	0.2696	4.0255	5.69E-05	5.60E-04
ENSBTAG00000016572	472.9355	1.0843	0.2963	3.6599	2.52E-04	2.04E-03
ENSBTAG00000013782	996.5061	1.0840	0.3153	3.4378	5.86E-04	4.20E-03
ENSBTAG00000007186	365.2627	1.0833	0.3326	3.2570	1.13E-03	7.11E-03
ENSBTAG00000000957	1068.1589	1.0800	0.2833	3.8124	1.38E-04	1.22E-03
ENSBTAG00000011895	966.2639	1.0794	0.3207	3.3653	7.65E-04	5.25E-03
ENSBTAG00000008695	1649.8417	1.0776	0.3136	3.4358	5.91E-04	4.22E-03
ENSBTAG00000010013	5113.5628	1.0742	0.2281	4.7096	2.48E-06	3.46E-05
ENSBTAG00000019495	579.7258	1.0719	0.3021	3.5484	3.88E-04	2.93E-03
ENSBTAG00000002291	486.7567	1.0678	0.2880	3.7077	2.09E-04	1.74E-03
ENSBTAG00000018898	707.5726	1.0675	0.2846	3.7507	1.76E-04	1.51E-03
ENSBTAG00000016733	653.1007	1.0665	0.2610	4.0864	4.38E-05	4.45E-04
ENSBTAG00000021573	5123.1555	1.0662	0.2577	4.1374	3.51E-05	3.68E-04
ENSBTAG00000047428	753.7068	1.0661	0.3194	3.3373	8.46E-04	5.68E-03
ENSBTAG00000009271	804.7538	1.0586	0.2878	3.6786	2.35E-04	1.92E-03
ENSBTAG00000009378	674.7399	1.0585	0.3127	3.3848	7.12E-04	4.94E-03
ENSBTAG00000037457	3261.8375	1.0555	0.3086	3.4204	6.25E-04	4.42E-03
ENSBTAG00000035230	1465.5874	1.0552	0.2424	4.3539	1.34E-05	1.58E-04
ENSBTAG00000019197	2197.9557	1.0532	0.3130	3.3653	7.65E-04	5.25E-03
ENSBTAG00000001529	2074.7976	1.0521	0.2400	4.3831	1.17E-05	1.40E-04
ENSBTAG00000031134	3158.3643	1.0499	0.2911	3.6065	3.10E-04	2.42E-03
ENSBTAG00000010784	1514.6158	1.0472	0.2138	4.8969	9.74E-07	1.51E-05
ENSBTAG00000034978	4322.1190	1.0458	0.3034	3.4472	5.67E-04	4.08E-03
ENSBTAG00000006072	4091.6381	1.0447	0.2869	3.6409	2.72E-04	2.17E-03
ENSBTAG00000046625	173.9781	1.0365	0.3176	3.2632	1.10E-03	6.99E-03
ENSBTAG00000011415	5401.5826	1.0359	0.1974	5.2476	1.54E-07	2.89E-06
ENSBTAG00000008854	810.8375	1.0346	0.2506	4.1292	3.64E-05	3.79E-04
ENSBTAG00000012890	17504.5391	1.0338	0.2648	3.9040	9.46E-05	8.74E-04
ENSBTAG00000001666	379.8340	1.0337	0.3090	3.3459	8.20E-04	5.54E-03
ENSBTAG00000007061	1572.1154	1.0311	0.2423	4.2550	2.09E-05	2.34E-04

ENSBTAG00000011067	1408.7989	1.0280	0.3177	3.2352	1.22E-03	7.59E-03
ENSBTAG00000016367	1053.0844	1.0272	0.2148	4.7829	1.73E-06	2.51E-05
ENSBTAG00000004496	3877.6234	1.0246	0.2666	3.8433	1.21E-04	1.09E-03
ENSBTAG00000016592	393.4910	1.0245	0.2890	3.5451	3.92E-04	2.96E-03
ENSBTAG00000015554	1584.6005	1.0244	0.2786	3.6768	2.36E-04	1.93E-03
ENSBTAG00000007935	3691.1501	1.0145	0.2093	4.8472	1.25E-06	1.89E-05
ENSBTAG00000013259	1990.5844	1.0131	0.2716	3.7303	1.91E-04	1.61E-03
ENSBTAG00000010810	1655.8366	1.0122	0.2784	3.6351	2.78E-04	2.20E-03
ENSBTAG00000020655	2480.8057	1.0112	0.2228	4.5376	5.69E-06	7.26E-05
ENSBTAG00000010951	641.2116	1.0078	0.3006	3.3527	8.00E-04	5.44E-03
ENSBTAG00000019133	2456.0281	1.0015	0.1960	5.1087	3.24E-07	5.62E-06
ENSBTAG00000009888	798.4222	-1.0003	0.2846	-3.5144	4.41E-04	3.28E-03
ENSBTAG00000011136	799.9974	-1.0011	0.3135	-3.1935	1.41E-03	8.55E-03
ENSBTAG00000040132	503.3164	-1.0063	0.3157	-3.1877	1.43E-03	8.68E-03
ENSBTAG00000009611	627.1732	-1.0104	0.2979	-3.3920	6.94E-04	4.82E-03
ENSBTAG00000015392	648.7636	-1.0116	0.2588	-3.9082	9.30E-05	8.60E-04
ENSBTAG00000009263	1150.4658	-1.0118	0.2929	-3.4540	5.52E-04	3.99E-03
ENSBTAG00000014015	1145.4513	-1.0121	0.3096	-3.2696	1.08E-03	6.88E-03
ENSBTAG00000014779	4070.1079	-1.0144	0.2856	-3.5524	3.82E-04	2.89E-03
ENSBTAG00000027764	2648.0042	-1.0146	0.2728	-3.7186	2.00E-04	1.68E-03
ENSBTAG00000014553	4378.5831	-1.0156	0.2580	-3.9358	8.29E-05	7.75E-04
ENSBTAG00000001950	1185.8056	-1.0172	0.2433	-4.1814	2.90E-05	3.11E-04
ENSBTAG00000030172	2582.7174	-1.0205	0.2353	-4.3378	1.44E-05	1.69E-04
ENSBTAG00000012024	541.4947	-1.0229	0.3025	-3.3818	7.20E-04	4.98E-03
ENSBTAG00000007315	1027.3436	-1.0236	0.3096	-3.3060	9.46E-04	6.19E-03
ENSBTAG00000030839	1857.1534	-1.0248	0.2278	-4.4982	6.85E-06	8.61E-05
ENSBTAG00000004328	2443.0018	-1.0254	0.2973	-3.4487	5.63E-04	4.06E-03
ENSBTAG00000006644	6573.7368	-1.0267	0.3007	-3.4145	6.39E-04	4.50E-03
ENSBTAG00000039916	8464.8794	-1.0278	0.3191	-3.2214	1.28E-03	7.91E-03
ENSBTAG00000025452	2236.4103	-1.0282	0.2902	-3.5431	3.96E-04	2.98E-03
ENSBTAG00000005787	629.5612	-1.0319	0.3281	-3.1450	1.66E-03	9.80E-03
ENSBTAG00000012723	407.3706	-1.0321	0.2946	-3.5040	4.58E-04	3.39E-03
ENSBTAG00000019780	2749.3173	-1.0325	0.2435	-4.2396	2.24E-05	2.47E-04
ENSBTAG00000007149	2402.9427	-1.0339	0.3009	-3.4356	5.91E-04	4.22E-03
ENSBTAG00000043956	888.1699	-1.0343	0.2877	-3.5947	3.25E-04	2.52E-03
ENSBTAG00000004430	695.1325	-1.0361	0.2756	-3.7600	1.70E-04	1.46E-03
ENSBTAG00000016501	1795.7944	-1.0405	0.2517	-4.1334	3.57E-05	3.73E-04
ENSBTAG00000003469	1554.3413	-1.0405	0.2910	-3.5753	3.50E-04	2.68E-03
ENSBTAG00000015611	2560.9257	-1.0437	0.2398	-4.3524	1.35E-05	1.59E-04
ENSBTAG00000005448	709.4574	-1.0476	0.2955	-3.5458	3.91E-04	2.95E-03
ENSBTAG00000019828	353.4147	-1.0502	0.2806	-3.7428	1.82E-04	1.55E-03
ENSBTAG00000009928	806.2952	-1.0513	0.2973	-3.5360	4.06E-04	3.05E-03
ENSBTAG00000017161	1583.5408	-1.0528	0.2167	-4.8574	1.19E-06	1.82E-05

ENSBTAG00000015734	693.0611	-1.0535	0.2883	-3.6545	2.58E-04	2.07E-03
ENSBTAG00000003679	642.9310	-1.0609	0.2954	-3.5913	3.29E-04	2.54E-03
ENSBTAG00000012789	2400.1347	-1.0635	0.3043	-3.4950	4.74E-04	3.49E-03
ENSBTAG00000025589	399.8222	-1.0664	0.2945	-3.6214	2.93E-04	2.30E-03
ENSBTAG00000019274	2549.9252	-1.0699	0.2801	-3.8202	1.33E-04	1.18E-03
ENSBTAG00000003746	2997.8210	-1.0702	0.2603	-4.1113	3.93E-05	4.05E-04
ENSBTAG00000006663	1484.6330	-1.0706	0.2998	-3.5715	3.55E-04	2.72E-03
ENSBTAG00000001780	490.1634	-1.0740	0.2941	-3.6524	2.60E-04	2.09E-03
ENSBTAG00000013096	1076.6496	-1.0764	0.2196	-4.9004	9.56E-07	1.49E-05
ENSBTAG00000013781	514.5503	-1.0810	0.2721	-3.9723	7.12E-05	6.82E-04
ENSBTAG00000014377	7797.1152	-1.0834	0.2461	-4.4016	1.07E-05	1.29E-04
ENSBTAG00000008730	305.6100	-1.0844	0.3138	-3.4558	5.49E-04	3.97E-03
ENSBTAG00000001036	2229.3621	-1.0894	0.2431	-4.4813	7.42E-06	9.26E-05
ENSBTAG00000017074	407.7327	-1.0899	0.3004	-3.6282	2.85E-04	2.25E-03
ENSBTAG00000014454	1287.2913	-1.0906	0.2137	-5.1030	3.34E-07	5.77E-06
ENSBTAG00000013244	1853.6904	-1.0911	0.2588	-4.2155	2.49E-05	2.72E-04
ENSBTAG00000039161	3217.0186	-1.0918	0.2001	-5.4555	4.88E-08	9.84E-07
ENSBTAG00000004922	1532.0626	-1.0959	0.3168	-3.4594	5.41E-04	3.92E-03
ENSBTAG00000018116	377.8324	-1.0971	0.3251	-3.3742	7.40E-04	5.11E-03
ENSBTAG00000012582	5806.5387	-1.1002	0.2665	-4.1281	3.66E-05	3.79E-04
ENSBTAG00000000026	532.0157	-1.1002	0.2752	-3.9980	6.39E-05	6.19E-04
ENSBTAG00000006034	471.7987	-1.1016	0.3479	-3.1670	1.54E-03	9.20E-03
ENSBTAG00000015117	285.2794	-1.1033	0.3495	-3.1571	1.59E-03	9.46E-03
ENSBTAG00000003885	765.9620	-1.1055	0.2562	-4.3145	1.60E-05	1.85E-04
ENSBTAG00000022167	501.2489	-1.1063	0.2503	-4.4198	9.88E-06	1.20E-04
ENSBTAG00000022294	2091.4681	-1.1106	0.2726	-4.0738	4.63E-05	4.65E-04
ENSBTAG00000000056	720.5330	-1.1143	0.2654	-4.1992	2.68E-05	2.90E-04
ENSBTAG00000015602	162.9813	-1.1152	0.3333	-3.3458	8.20E-04	5.54E-03
ENSBTAG00000036262	1148.6243	-1.1167	0.2741	-4.0748	4.61E-05	4.65E-04
ENSBTAG00000026199	66012.1948	-1.1183	0.3232	-3.4606	5.39E-04	3.90E-03
ENSBTAG00000005617	130.9831	-1.1186	0.3470	-3.2236	1.27E-03	7.86E-03
ENSBTAG00000009813	3660.8827	-1.1198	0.2457	-4.5566	5.20E-06	6.70E-05
ENSBTAG00000016349	896.8558	-1.1200	0.3162	-3.5420	3.97E-04	2.99E-03
ENSBTAG00000004631	547.9909	-1.1213	0.2654	-4.2250	2.39E-05	2.61E-04
ENSBTAG00000012490	2122.1292	-1.1220	0.3105	-3.6131	3.03E-04	2.37E-03
ENSBTAG00000033412	402.2951	-1.1235	0.3269	-3.4369	5.88E-04	4.21E-03
ENSBTAG00000014170	938.7345	-1.1243	0.3536	-3.1795	1.48E-03	8.88E-03
ENSBTAG00000005635	243.3542	-1.1258	0.3491	-3.2250	1.26E-03	7.83E-03
ENSBTAG00000014729	1087.5572	-1.1275	0.2566	-4.3935	1.12E-05	1.34E-04
ENSBTAG00000005683	625.8445	-1.1346	0.2987	-3.7990	1.45E-04	1.27E-03
ENSBTAG00000016240	487.8972	-1.1364	0.3170	-3.5845	3.38E-04	2.60E-03
ENSBTAG00000004907	1354.1849	-1.1412	0.2351	-4.8533	1.21E-06	1.85E-05
ENSBTAG00000016369	606.3103	-1.1446	0.3480	-3.2888	1.01E-03	6.50E-03

ENSBTAG00000011108	507.4691	-1.1472	0.3438	-3.3363	8.49E-04	5.69E-03
ENSBTAG00000007013	227.1343	-1.1502	0.3428	-3.3554	7.92E-04	5.40E-03
ENSBTAG00000044150	508.7437	-1.1502	0.3363	-3.4204	6.25E-04	4.42E-03
ENSBTAG00000009855	3461.9400	-1.1526	0.2796	-4.1224	3.75E-05	3.88E-04
ENSBTAG00000002714	545.1648	-1.1529	0.3203	-3.5992	3.19E-04	2.48E-03
ENSBTAG00000010132	644.0699	-1.1546	0.3085	-3.7431	1.82E-04	1.54E-03
ENSBTAG00000013287	3017.6181	-1.1547	0.3618	-3.1916	1.41E-03	8.59E-03
ENSBTAG00000019419	1650.8824	-1.1549	0.3022	-3.8215	1.33E-04	1.18E-03
ENSBTAG00000009698	2249.5292	-1.1559	0.2687	-4.3020	1.69E-05	1.94E-04
ENSBTAG00000002073	1030.3226	-1.1581	0.2553	-4.5369	5.71E-06	7.28E-05
ENSBTAG00000013414	4672.8644	-1.1602	0.3601	-3.2221	1.27E-03	7.90E-03
ENSBTAG00000014417	721.4464	-1.1623	0.3639	-3.1936	1.41E-03	8.55E-03
ENSBTAG00000003589	565.3844	-1.1627	0.3118	-3.7286	1.93E-04	1.62E-03
ENSBTAG00000009374	803.5970	-1.1657	0.2937	-3.9694	7.21E-05	6.89E-04
ENSBTAG00000013465	737.8764	-1.1676	0.3631	-3.2156	1.30E-03	8.05E-03
ENSBTAG00000007147	311.3780	-1.1710	0.3289	-3.5607	3.70E-04	2.81E-03
ENSBTAG00000020617	2168.1190	-1.1711	0.3088	-3.7920	1.49E-04	1.30E-03
ENSBTAG00000012272	1173.2763	-1.1713	0.3070	-3.8155	1.36E-04	1.20E-03
ENSBTAG00000013726	520.6401	-1.1722	0.3593	-3.2622	1.11E-03	7.01E-03
ENSBTAG00000009127	1904.5212	-1.1762	0.2907	-4.0454	5.22E-05	5.18E-04
ENSBTAG00000007364	146.4769	-1.1773	0.3479	-3.3843	7.14E-04	4.95E-03
ENSBTAG00000007271	331.2024	-1.1819	0.3139	-3.7656	1.66E-04	1.43E-03
ENSBTAG00000015490	269.9353	-1.1830	0.3554	-3.3289	8.72E-04	5.79E-03
ENSBTAG00000010460	4409.3655	-1.1861	0.3291	-3.6043	3.13E-04	2.44E-03
ENSBTAG00000018041	1560.7341	-1.1869	0.2375	-4.9972	5.82E-07	9.44E-06
ENSBTAG00000000371	2222.5727	-1.1911	0.2596	-4.5880	4.48E-06	5.87E-05
ENSBTAG00000040338	28274.7900	-1.1932	0.3574	-3.3390	8.41E-04	5.66E-03
ENSBTAG00000013274	313.3984	-1.1968	0.3397	-3.5228	4.27E-04	3.19E-03
ENSBTAG00000020646	278.1602	-1.1977	0.3653	-3.2786	1.04E-03	6.70E-03
ENSBTAG00000016969	872.9898	-1.1983	0.2617	-4.5786	4.68E-06	6.11E-05
ENSBTAG00000004203	600.6900	-1.2000	0.2546	-4.7129	2.44E-06	3.41E-05
ENSBTAG00000017082	13294.4217	-1.2003	0.3699	-3.2453	1.17E-03	7.37E-03
ENSBTAG00000001551	609.8057	-1.2039	0.3684	-3.2681	1.08E-03	6.91E-03
ENSBTAG00000043961	974.9219	-1.2050	0.3135	-3.8431	1.21E-04	1.09E-03
ENSBTAG00000030175	714.6352	-1.2069	0.2754	-4.3817	1.18E-05	1.41E-04
ENSBTAG00000013663	1051.4599	-1.2088	0.3214	-3.7613	1.69E-04	1.45E-03
ENSBTAG00000012511	1026.1653	-1.2097	0.3528	-3.4285	6.07E-04	4.31E-03
ENSBTAG00000021658	413.6447	-1.2100	0.3759	-3.2186	1.29E-03	7.98E-03
ENSBTAG00000008222	200.2928	-1.2104	0.3348	-3.6153	3.00E-04	2.35E-03
ENSBTAG00000019217	1181.9056	-1.2106	0.2546	-4.7546	1.99E-06	2.83E-05
ENSBTAG00000003491	223.1893	-1.2139	0.3506	-3.4620	5.36E-04	3.89E-03
ENSBTAG00000019177	910.1197	-1.2143	0.3468	-3.5019	4.62E-04	3.41E-03
ENSBTAG00000012982	509.3225	-1.2149	0.2603	-4.6668	3.06E-06	4.18E-05

ENSBTAG00000010867	1192.0213	-1.2225	0.2073	-5.8980	3.68E-09	9.06E-08
ENSBTAG00000038620	761.7442	-1.2237	0.2300	-5.3195	1.04E-07	2.00E-06
ENSBTAG00000013311	573.8566	-1.2239	0.3494	-3.5033	4.59E-04	3.40E-03
ENSBTAG00000001847	270.1388	-1.2239	0.3897	-3.1404	1.69E-03	9.93E-03
ENSBTAG00000012940	279.0856	-1.2246	0.3678	-3.3298	8.69E-04	5.78E-03
ENSBTAG00000021586	207.5915	-1.2276	0.3690	-3.3264	8.80E-04	5.84E-03
ENSBTAG00000012012	1968.1751	-1.2280	0.2954	-4.1565	3.23E-05	3.43E-04
ENSBTAG00000016967	1120.6687	-1.2280	0.2992	-4.1038	4.06E-05	4.17E-04
ENSBTAG00000000899	879.1412	-1.2292	0.3704	-3.3188	9.04E-04	5.96E-03
ENSBTAG00000043581	579.1582	-1.2294	0.3589	-3.4256	6.13E-04	4.35E-03
ENSBTAG00000016139	17065.3704	-1.2322	0.3483	-3.5377	4.04E-04	3.03E-03
ENSBTAG00000003718	266.8764	-1.2382	0.2901	-4.2681	1.97E-05	2.21E-04
ENSBTAG00000013489	766.1573	-1.2396	0.3401	-3.6447	2.68E-04	2.14E-03
ENSBTAG00000002813	1120.7326	-1.2421	0.2792	-4.4480	8.67E-06	1.07E-04
ENSBTAG00000018690	2319.0062	-1.2428	0.2795	-4.4472	8.70E-06	1.07E-04
ENSBTAG00000003813	378.9314	-1.2469	0.3333	-3.7414	1.83E-04	1.55E-03
ENSBTAG00000017662	953.0755	-1.2475	0.3442	-3.6243	2.90E-04	2.28E-03
ENSBTAG00000020642	898.1267	-1.2512	0.2833	-4.4167	1.00E-05	1.22E-04
ENSBTAG00000011709	434.7218	-1.2557	0.3131	-4.0107	6.05E-05	5.93E-04
ENSBTAG00000038691	1206.9780	-1.2589	0.2379	-5.2910	1.22E-07	2.31E-06
ENSBTAG00000009022	405.4926	-1.2601	0.3320	-3.7951	1.48E-04	1.29E-03
ENSBTAG00000019615	1170.7361	-1.2603	0.2447	-5.1494	2.61E-07	4.65E-06
ENSBTAG00000032961	6391.9255	-1.2623	0.2021	-6.2472	4.18E-10	1.25E-08
ENSBTAG00000001364	249.6468	-1.2655	0.3811	-3.3205	8.99E-04	5.94E-03
ENSBTAG00000020498	2394.6294	-1.2663	0.2736	-4.6273	3.70E-06	4.97E-05
ENSBTAG00000018796	675.8044	-1.2665	0.3302	-3.8360	1.25E-04	1.12E-03
ENSBTAG00000010698	479.1501	-1.2668	0.3460	-3.6613	2.51E-04	2.03E-03
ENSBTAG00000032852	198.7170	-1.2693	0.3791	-3.3481	8.14E-04	5.51E-03
ENSBTAG00000004064	600.8401	-1.2701	0.3631	-3.4980	4.69E-04	3.45E-03
ENSBTAG00000004790	282.8256	-1.2712	0.2962	-4.2920	1.77E-05	2.01E-04
ENSBTAG00000006995	4832.4662	-1.2715	0.3720	-3.4178	6.31E-04	4.46E-03
ENSBTAG00000018971	167.1342	-1.2735	0.3823	-3.3314	8.64E-04	5.76E-03
ENSBTAG00000018415	642.6062	-1.2748	0.3736	-3.4123	6.44E-04	4.53E-03
ENSBTAG00000014923	1017.0703	-1.2767	0.2728	-4.6792	2.88E-06	3.96E-05
ENSBTAG00000012126	315.4053	-1.2795	0.3600	-3.5544	3.79E-04	2.87E-03
ENSBTAG00000014007	1303.8847	-1.2868	0.2523	-5.1009	3.38E-07	5.83E-06
ENSBTAG00000000501	366.5225	-1.2890	0.3076	-4.1905	2.78E-05	3.00E-04
ENSBTAG00000012632	8902.1169	-1.2921	0.3998	-3.2315	1.23E-03	7.68E-03
ENSBTAG00000013152	1001.2632	-1.2953	0.3274	-3.9564	7.61E-05	7.19E-04
ENSBTAG00000021102	362.1478	-1.2959	0.2608	-4.9698	6.70E-07	1.07E-05
ENSBTAG00000021829	324.4382	-1.2993	0.3704	-3.5082	4.51E-04	3.35E-03
ENSBTAG00000012926	782.5375	-1.3014	0.3991	-3.2610	1.11E-03	7.03E-03
ENSBTAG00000008836	109.6296	-1.3063	0.4117	-3.1725	1.51E-03	9.05E-03

ENSBTAG00000015019	301.3908	-1.3075	0.4082	-3.2029	1.36E-03	8.36E-03
ENSBTAG00000005827	425.8612	-1.3105	0.3192	-4.1054	4.04E-05	4.15E-04
ENSBTAG00000005676	1710.3934	-1.3124	0.3278	-4.0033	6.25E-05	6.09E-04
ENSBTAG00000039138	1845.7366	-1.3137	0.2707	-4.8522	1.22E-06	1.86E-05
ENSBTAG00000003245	1729.9639	-1.3143	0.4010	-3.2778	1.05E-03	6.71E-03
ENSBTAG00000009477	693.9115	-1.3189	0.3228	-4.0863	4.38E-05	4.45E-04
ENSBTAG00000015728	685.0403	-1.3189	0.2571	-5.1296	2.90E-07	5.11E-06
ENSBTAG00000020527	12689.6281	-1.3242	0.3426	-3.8650	1.11E-04	1.01E-03
ENSBTAG00000044079	365.4380	-1.3308	0.3602	-3.6943	2.21E-04	1.82E-03
ENSBTAG00000014372	1453.6652	-1.3311	0.2779	-4.7901	1.67E-06	2.43E-05
ENSBTAG00000015158	471.3088	-1.3324	0.3397	-3.9226	8.76E-05	8.15E-04
ENSBTAG00000021019	341.9630	-1.3327	0.3533	-3.7717	1.62E-04	1.40E-03
ENSBTAG00000008943	249.3138	-1.3371	0.3480	-3.8426	1.22E-04	1.09E-03
ENSBTAG00000007133	1045.6067	-1.3402	0.2760	-4.8553	1.20E-06	1.83E-05
ENSBTAG00000013530	557.9079	-1.3414	0.3138	-4.2753	1.91E-05	2.15E-04
ENSBTAG00000007371	349.9716	-1.3425	0.3323	-4.0395	5.36E-05	5.30E-04
ENSBTAG00000011512	299.7586	-1.3436	0.3965	-3.3882	7.03E-04	4.88E-03
ENSBTAG00000000942	1453.8171	-1.3438	0.4146	-3.2411	1.19E-03	7.46E-03
ENSBTAG00000015894	117.5884	-1.3448	0.4211	-3.1937	1.40E-03	8.55E-03
ENSBTAG00000006212	158.4959	-1.3466	0.3578	-3.7639	1.67E-04	1.44E-03
ENSBTAG00000018299	535.1230	-1.3472	0.3524	-3.8232	1.32E-04	1.17E-03
ENSBTAG00000019071	648.2771	-1.3480	0.2565	-5.2560	1.47E-07	2.77E-06
ENSBTAG00000005385	515.9548	-1.3494	0.3725	-3.6221	2.92E-04	2.30E-03
ENSBTAG00000016156	2044.9322	-1.3500	0.2378	-5.6761	1.38E-08	3.08E-07
ENSBTAG00000047809	504.8858	-1.3521	0.3978	-3.3985	6.78E-04	4.73E-03
ENSBTAG00000006222	204.8329	-1.3526	0.4255	-3.1789	1.48E-03	8.89E-03
ENSBTAG00000034269	413.4223	-1.3544	0.3050	-4.4412	8.95E-06	1.09E-04
ENSBTAG00000000251	364.7892	-1.3566	0.4275	-3.1733	1.51E-03	9.03E-03
ENSBTAG00000021205	1343.8574	-1.3589	0.3994	-3.4022	6.68E-04	4.67E-03
ENSBTAG00000015097	1007.8680	-1.3589	0.2652	-5.1236	3.00E-07	5.25E-06
ENSBTAG00000012885	2737.7686	-1.3633	0.2919	-4.6713	2.99E-06	4.10E-05
ENSBTAG00000006775	4828.1123	-1.3642	0.4265	-3.1983	1.38E-03	8.48E-03
ENSBTAG00000017945	139.1367	-1.3645	0.3606	-3.7842	1.54E-04	1.34E-03
ENSBTAG00000008306	4464.1522	-1.3647	0.2483	-5.4951	3.90E-08	7.98E-07
ENSBTAG00000013653	43957.8112	-1.3658	0.4169	-3.2762	1.05E-03	6.75E-03
ENSBTAG00000001104	223.2037	-1.3673	0.3565	-3.8350	1.26E-04	1.12E-03
ENSBTAG00000013127	1372.0186	-1.3677	0.3971	-3.4445	5.72E-04	4.11E-03
ENSBTAG00000012030	225.0859	-1.3707	0.2953	-4.6419	3.45E-06	4.66E-05
ENSBTAG00000017566	209.9704	-1.3731	0.3267	-4.2028	2.64E-05	2.86E-04
ENSBTAG00000003504	985.9071	-1.3760	0.3776	-3.6440	2.68E-04	2.15E-03
ENSBTAG00000017554	259.4623	-1.3779	0.3494	-3.9429	8.05E-05	7.57E-04
ENSBTAG00000011969	25790.7860	-1.3798	0.4361	-3.1641	1.56E-03	9.26E-03
ENSBTAG00000001640	519.3898	-1.3846	0.3397	-4.0760	4.58E-05	4.63E-04

ENSBTAG00000002742	784.8761	-1.3883	0.3334	-4.1643	3.12E-05	3.33E-04
ENSBTAG00000020371	313.8887	-1.3897	0.3348	-4.1507	3.31E-05	3.50E-04
ENSBTAG00000010449	2875.8163	-1.3912	0.4184	-3.3250	8.84E-04	5.86E-03
ENSBTAG00000012241	742.2754	-1.3915	0.3622	-3.8419	1.22E-04	1.09E-03
ENSBTAG00000018513	884.5092	-1.3954	0.4160	-3.3543	7.96E-04	5.41E-03
ENSBTAG00000005249	1247.9176	-1.3956	0.2761	-5.0539	4.33E-07	7.26E-06
ENSBTAG00000006489	939.3451	-1.3993	0.3045	-4.5953	4.32E-06	5.69E-05
ENSBTAG00000025893	4636.9439	-1.4027	0.3155	-4.4464	8.73E-06	1.07E-04
ENSBTAG00000025931	550.8488	-1.4044	0.3696	-3.7992	1.45E-04	1.27E-03
ENSBTAG00000017571	244.7361	-1.4085	0.3851	-3.6576	2.55E-04	2.05E-03
ENSBTAG00000012365	2250.0865	-1.4129	0.3532	-4.0000	6.33E-05	6.15E-04
ENSBTAG00000000231	1804.0746	-1.4133	0.2715	-5.2057	1.93E-07	3.55E-06
ENSBTAG00000006526	682.4802	-1.4169	0.2489	-5.6932	1.25E-08	2.82E-07
ENSBTAG00000015221	2422.1838	-1.4174	0.2987	-4.7448	2.09E-06	2.96E-05
ENSBTAG00000010348	675.3308	-1.4174	0.3472	-4.0825	4.46E-05	4.52E-04
ENSBTAG00000032951	604.6076	-1.4177	0.3130	-4.5289	5.93E-06	7.54E-05
ENSBTAG00000026842	211.2076	-1.4215	0.4167	-3.4113	6.47E-04	4.55E-03
ENSBTAG00000011885	4731.5625	-1.4220	0.1979	-7.1849	6.73E-13	3.12E-11
ENSBTAG00000021798	721.9735	-1.4222	0.4474	-3.1792	1.48E-03	8.88E-03
ENSBTAG00000001598	158.9813	-1.4227	0.3608	-3.9437	8.02E-05	7.56E-04
ENSBTAG00000015805	2225.1580	-1.4358	0.2786	-5.1542	2.55E-07	4.56E-06
ENSBTAG00000045925	5053.4330	-1.4386	0.4421	-3.2540	1.14E-03	7.18E-03
ENSBTAG00000012800	202.8714	-1.4396	0.3588	-4.0118	6.03E-05	5.91E-04
ENSBTAG00000011648	174.7433	-1.4401	0.4171	-3.4525	5.55E-04	4.01E-03
ENSBTAG00000007622	11493.6391	-1.4409	0.4421	-3.2596	1.12E-03	7.07E-03
ENSBTAG00000025809	758.3147	-1.4411	0.4148	-3.4742	5.12E-04	3.73E-03
ENSBTAG00000008173	835.0316	-1.4430	0.2820	-5.1173	3.10E-07	5.41E-06
ENSBTAG00000007684	1216.0388	-1.4451	0.3402	-4.2480	2.16E-05	2.40E-04
ENSBTAG00000012729	154.5693	-1.4482	0.4612	-3.1397	1.69E-03	9.95E-03
ENSBTAG00000015131	31970.3261	-1.4503	0.2956	-4.9070	9.25E-07	1.44E-05
ENSBTAG00000021158	451.1629	-1.4538	0.3632	-4.0026	6.27E-05	6.09E-04
ENSBTAG00000004471	1803.7318	-1.4549	0.2612	-5.5710	2.53E-08	5.33E-07
ENSBTAG00000014601	4589.4660	-1.4556	0.2773	-5.2484	1.53E-07	2.88E-06
ENSBTAG00000011819	865.1849	-1.4559	0.2842	-5.1236	3.00E-07	5.25E-06
ENSBTAG00000015980	7231.0415	-1.4588	0.2957	-4.9335	8.08E-07	1.27E-05
ENSBTAG00000000532	196.5773	-1.4602	0.3728	-3.9164	8.99E-05	8.33E-04
ENSBTAG00000039968	197.6484	-1.4606	0.3370	-4.3337	1.47E-05	1.71E-04
ENSBTAG00000008346	418.2696	-1.4621	0.3320	-4.4041	1.06E-05	1.28E-04
ENSBTAG00000007220	1156.6415	-1.4647	0.3235	-4.5281	5.95E-06	7.56E-05
ENSBTAG00000003607	967.7802	-1.4669	0.3424	-4.2846	1.83E-05	2.07E-04
ENSBTAG00000021065	467.4725	-1.4734	0.3997	-3.6866	2.27E-04	1.87E-03
ENSBTAG00000006985	1110.6514	-1.4809	0.2717	-5.4506	5.02E-08	1.01E-06
ENSBTAG00000008275	4852.5281	-1.4831	0.3324	-4.4622	8.11E-06	1.00E-04

ENSBTAG00000036101	129.3037	-1.4881	0.4051	-3.6736	2.39E-04	1.95E-03
ENSBTAG00000017867	88.2650	-1.4897	0.4229	-3.5221	4.28E-04	3.19E-03
ENSBTAG00000007933	187.9444	-1.4900	0.4353	-3.4230	6.19E-04	4.39E-03
ENSBTAG00000038845	96.5036	-1.4906	0.4615	-3.2298	1.24E-03	7.71E-03
ENSBTAG00000003199	112.5138	-1.4907	0.4492	-3.3190	9.03E-04	5.96E-03
ENSBTAG00000011104	6276.1712	-1.4914	0.2400	-6.2135	5.18E-10	1.51E-08
ENSBTAG00000005373	6480.7183	-1.4926	0.3925	-3.8024	1.43E-04	1.26E-03
ENSBTAG00000040569	118.9623	-1.4960	0.4115	-3.6352	2.78E-04	2.20E-03
ENSBTAG00000009655	2904.5571	-1.4975	0.3135	-4.7773	1.78E-06	2.57E-05
ENSBTAG00000012243	2199.8561	-1.4991	0.4619	-3.2458	1.17E-03	7.36E-03
ENSBTAG00000016724	816.9109	-1.5017	0.3507	-4.2823	1.85E-05	2.09E-04
ENSBTAG00000005138	1357.7937	-1.5045	0.2115	-7.1139	1.13E-12	5.02E-11
ENSBTAG00000018192	1161.0187	-1.5052	0.4042	-3.7241	1.96E-04	1.65E-03
ENSBTAG00000024107	453.5479	-1.5090	0.3779	-3.9934	6.51E-05	6.30E-04
ENSBTAG00000004425	108.8864	-1.5091	0.4724	-3.1946	1.40E-03	8.53E-03
ENSBTAG00000031277	1009.7219	-1.5096	0.3072	-4.9137	8.94E-07	1.39E-05
ENSBTAG00000021301	964.7847	-1.5109	0.4216	-3.5836	3.39E-04	2.61E-03
ENSBTAG00000017067	2301.7699	-1.5162	0.4611	-3.2881	1.01E-03	6.50E-03
ENSBTAG00000019552	6383.2081	-1.5167	0.3535	-4.2902	1.79E-05	2.02E-04
ENSBTAG00000017502	882.0180	-1.5167	0.2787	-5.4422	5.26E-08	1.05E-06
ENSBTAG00000011072	1673.3140	-1.5196	0.2337	-6.5034	7.85E-11	2.68E-09
ENSBTAG00000002515	97.7696	-1.5198	0.4646	-3.2709	1.07E-03	6.86E-03
ENSBTAG00000006054	100.3940	-1.5214	0.4533	-3.3566	7.89E-04	5.39E-03
ENSBTAG00000004281	1167.0957	-1.5214	0.4415	-3.4462	5.68E-04	4.09E-03
ENSBTAG00000002863	1226.0366	-1.5216	0.2322	-6.5535	5.62E-11	1.95E-09
ENSBTAG00000012900	7309.7925	-1.5231	0.3548	-4.2926	1.77E-05	2.01E-04
ENSBTAG00000032711	157.8157	-1.5271	0.3542	-4.3109	1.63E-05	1.88E-04
ENSBTAG00000000037	262.2195	-1.5274	0.3402	-4.4897	7.13E-06	8.94E-05
ENSBTAG00000012087	90.0868	-1.5284	0.4479	-3.4124	6.44E-04	4.53E-03
ENSBTAG00000033180	130.1520	-1.5287	0.3882	-3.9385	8.20E-05	7.69E-04
ENSBTAG00000006966	1129.6971	-1.5319	0.4034	-3.7977	1.46E-04	1.28E-03
ENSBTAG00000004622	876.3293	-1.5321	0.3413	-4.4897	7.13E-06	8.94E-05
ENSBTAG00000021372	609.9971	-1.5336	0.3288	-4.6639	3.10E-06	4.22E-05
ENSBTAG00000005934	333.5528	-1.5354	0.3931	-3.9062	9.37E-05	8.66E-04
ENSBTAG00000004011	149.7469	-1.5431	0.4785	-3.2249	1.26E-03	7.83E-03
ENSBTAG00000037673	175.1287	-1.5432	0.4664	-3.3091	9.36E-04	6.14E-03
ENSBTAG00000019010	313.0357	-1.5459	0.3925	-3.9383	8.21E-05	7.69E-04
ENSBTAG00000004409	1855.9531	-1.5459	0.3234	-4.7802	1.75E-06	2.53E-05
ENSBTAG00000033217	47064.8258	-1.5525	0.2728	-5.6910	1.26E-08	2.85E-07
ENSBTAG00000020791	1214.9071	-1.5552	0.4234	-3.6729	2.40E-04	1.95E-03
ENSBTAG00000019045	583.5110	-1.5570	0.3324	-4.6847	2.80E-06	3.87E-05
ENSBTAG00000001385	296.8545	-1.5588	0.3438	-4.5334	5.80E-06	7.39E-05
ENSBTAG00000012566	74.0986	-1.5600	0.4673	-3.3381	8.43E-04	5.67E-03

ENSBTAG00000002186	831.6380	-1.5605	0.4654	-3.3532	7.99E-04	5.43E-03
ENSBTAG00000000831	200.7842	-1.5627	0.3515	-4.4453	8.78E-06	1.08E-04
ENSBTAG00000021803	748.0747	-1.5637	0.4359	-3.5872	3.34E-04	2.58E-03
ENSBTAG00000020980	1733.2693	-1.5638	0.3896	-4.0142	5.97E-05	5.85E-04
ENSBTAG00000011893	1402.7811	-1.5643	0.3606	-4.3375	1.44E-05	1.69E-04
ENSBTAG00000003076	308.5136	-1.5735	0.3876	-4.0600	4.91E-05	4.91E-04
ENSBTAG00000001721	339.3462	-1.5740	0.3298	-4.7718	1.83E-06	2.61E-05
ENSBTAG00000002258	13381.9094	-1.5740	0.3140	-5.0131	5.36E-07	8.77E-06
ENSBTAG00000007107	99.7166	-1.5764	0.4527	-3.4825	4.97E-04	3.63E-03
ENSBTAG00000020346	110.7599	-1.5772	0.4198	-3.7575	1.72E-04	1.47E-03
ENSBTAG00000013478	1232.9498	-1.5780	0.3370	-4.6823	2.84E-06	3.91E-05
ENSBTAG00000020223	1789.7073	-1.5784	0.3316	-4.7595	1.94E-06	2.77E-05
ENSBTAG00000005595	2174.4175	-1.5849	0.2560	-6.1901	6.01E-10	1.74E-08
ENSBTAG00000000447	353.0065	-1.5856	0.4284	-3.7008	2.15E-04	1.78E-03
ENSBTAG00000009017	3443.4663	-1.5867	0.3326	-4.7706	1.84E-06	2.62E-05
ENSBTAG00000013485	115.4803	-1.5870	0.3649	-4.3489	1.37E-05	1.61E-04
ENSBTAG00000021859	214.4948	-1.5872	0.3787	-4.1909	2.78E-05	3.00E-04
ENSBTAG00000000341	986.1031	-1.5888	0.2901	-5.4775	4.31E-08	8.76E-07
ENSBTAG00000012096	548.2937	-1.5919	0.2698	-5.9011	3.61E-09	8.92E-08
ENSBTAG00000012955	78.5152	-1.5920	0.4981	-3.1964	1.39E-03	8.50E-03
ENSBTAG00000018373	10955.3752	-1.5958	0.3300	-4.8354	1.33E-06	1.99E-05
ENSBTAG00000012504	3393.5343	-1.6003	0.3714	-4.3089	1.64E-05	1.89E-04
ENSBTAG00000005478	1411.9233	-1.6089	0.3388	-4.7485	2.05E-06	2.91E-05
ENSBTAG00000017051	212.2213	-1.6093	0.4337	-3.7103	2.07E-04	1.72E-03
ENSBTAG00000038085	151.2000	-1.6120	0.4972	-3.2422	1.19E-03	7.44E-03
ENSBTAG00000018688	65.7438	-1.6142	0.4827	-3.3442	8.25E-04	5.56E-03
ENSBTAG00000015807	290.9201	-1.6163	0.3646	-4.4334	9.28E-06	1.13E-04
ENSBTAG00000009021	1321.6204	-1.6172	0.2592	-6.2393	4.40E-10	1.31E-08
ENSBTAG00000021768	552.2400	-1.6173	0.3820	-4.2337	2.30E-05	2.52E-04
ENSBTAG00000018661	1552.1824	-1.6197	0.3415	-4.7425	2.11E-06	2.99E-05
ENSBTAG00000006754	143.7829	-1.6208	0.3425	-4.7326	2.22E-06	3.11E-05
ENSBTAG00000018178	670.2074	-1.6242	0.3556	-4.5669	4.95E-06	6.42E-05
ENSBTAG00000002417	566.3611	-1.6302	0.4978	-3.2751	1.06E-03	6.77E-03
ENSBTAG00000005370	1479.9848	-1.6313	0.4102	-3.9768	6.99E-05	6.72E-04
ENSBTAG00000010657	2877.1034	-1.6338	0.3996	-4.0884	4.34E-05	4.43E-04
ENSBTAG00000046989	75.2985	-1.6344	0.4626	-3.5334	4.10E-04	3.08E-03
ENSBTAG00000008109	5241.0582	-1.6352	0.3304	-4.9490	7.46E-07	1.18E-05
ENSBTAG00000024240	2297.2039	-1.6368	0.3074	-5.3251	1.01E-07	1.95E-06
ENSBTAG00000046156	214.8647	-1.6397	0.4903	-3.3443	8.25E-04	5.56E-03
ENSBTAG00000016472	304.8036	-1.6402	0.4077	-4.0231	5.74E-05	5.66E-04
ENSBTAG00000014907	370.2214	-1.6402	0.2861	-5.7324	9.90E-09	2.28E-07
ENSBTAG00000008424	7553.6495	-1.6425	0.2813	-5.8380	5.28E-09	1.27E-07
ENSBTAG00000017833	7211.4712	-1.6506	0.2703	-6.1061	1.02E-09	2.82E-08

ENSBTAG00000010472	401.9156	-1.6514	0.3296	-5.0098	5.45E-07	8.88E-06
ENSBTAG00000008492	2497.2761	-1.6517	0.2676	-6.1711	6.78E-10	1.93E-08
ENSBTAG00000030432	321.7360	-1.6548	0.3217	-5.1441	2.69E-07	4.78E-06
ENSBTAG00000016486	731.3218	-1.6555	0.4243	-3.9012	9.57E-05	8.82E-04
ENSBTAG00000000905	861.2332	-1.6582	0.4066	-4.0786	4.53E-05	4.58E-04
ENSBTAG00000006610	53.2710	-1.6593	0.5207	-3.1867	1.44E-03	8.69E-03
ENSBTAG00000016731	350.8907	-1.6602	0.4176	-3.9755	7.02E-05	6.74E-04
ENSBTAG00000010326	123.1892	-1.6625	0.4885	-3.4029	6.67E-04	4.66E-03
ENSBTAG00000009111	424.0193	-1.6645	0.3778	-4.4055	1.06E-05	1.27E-04
ENSBTAG00000016277	1439.4225	-1.6652	0.4701	-3.5420	3.97E-04	2.99E-03
ENSBTAG00000005203	149.5372	-1.6658	0.4035	-4.1288	3.65E-05	3.79E-04
ENSBTAG00000014945	51.1291	-1.6672	0.5267	-3.1653	1.55E-03	9.24E-03
ENSBTAG00000006209	3032.4739	-1.6704	0.4946	-3.3774	7.32E-04	5.06E-03
ENSBTAG00000021036	1089.5160	-1.6831	0.3995	-4.2132	2.52E-05	2.75E-04
ENSBTAG00000019382	1032.7279	-1.6835	0.4732	-3.5577	3.74E-04	2.84E-03
ENSBTAG00000016455	286.3828	-1.6862	0.5143	-3.2786	1.04E-03	6.70E-03
ENSBTAG00000005129	970.0957	-1.6866	0.2570	-6.5630	5.27E-11	1.85E-09
ENSBTAG00000013109	4042.4593	-1.6868	0.3189	-5.2901	1.22E-07	2.32E-06
ENSBTAG00000011413	624.7854	-1.6879	0.4071	-4.1463	3.38E-05	3.56E-04
ENSBTAG00000004222	603.4519	-1.6904	0.4246	-3.9809	6.87E-05	6.62E-04
ENSBTAG00000016484	1791.3604	-1.6990	0.4765	-3.5655	3.63E-04	2.77E-03
ENSBTAG00000012380	4567.3802	-1.7056	0.2861	-5.9607	2.51E-09	6.49E-08
ENSBTAG00000008682	509.3291	-1.7069	0.4554	-3.7479	1.78E-04	1.52E-03
ENSBTAG00000046552	102.9456	-1.7229	0.4599	-3.7467	1.79E-04	1.53E-03
ENSBTAG00000018555	50.3115	-1.7321	0.5472	-3.1655	1.55E-03	9.24E-03
ENSBTAG00000021921	1969.6760	-1.7321	0.2819	-6.1437	8.06E-10	2.26E-08
ENSBTAG00000015276	1241.3832	-1.7322	0.4281	-4.0464	5.20E-05	5.16E-04
ENSBTAG00000015527	1433.4125	-1.7384	0.4345	-4.0006	6.32E-05	6.13E-04
ENSBTAG00000008884	458.1494	-1.7398	0.3684	-4.7226	2.33E-06	3.25E-05
ENSBTAG00000021992	101.4887	-1.7403	0.4723	-3.6849	2.29E-04	1.88E-03
ENSBTAG00000001770	167.0767	-1.7437	0.5077	-3.4347	5.93E-04	4.23E-03
ENSBTAG00000011400	994.4450	-1.7442	0.3820	-4.5663	4.96E-06	6.44E-05
ENSBTAG00000008348	247.9311	-1.7450	0.3618	-4.8226	1.42E-06	2.10E-05
ENSBTAG00000005633	4458.0156	-1.7487	0.5353	-3.2668	1.09E-03	6.92E-03
ENSBTAG00000047461	190.3375	-1.7504	0.4420	-3.9604	7.48E-05	7.11E-04
ENSBTAG00000019075	64.6588	-1.7510	0.4769	-3.6716	2.41E-04	1.96E-03
ENSBTAG00000047717	655.4102	-1.7531	0.5252	-3.3378	8.44E-04	5.67E-03
ENSBTAG00000018181	3099.0358	-1.7533	0.3017	-5.8112	6.20E-09	1.47E-07
ENSBTAG00000014958	9984.8631	-1.7612	0.3718	-4.7375	2.16E-06	3.05E-05
ENSBTAG00000010254	71.6223	-1.7637	0.5362	-3.2894	1.00E-03	6.49E-03
ENSBTAG00000016629	481.1958	-1.7642	0.4459	-3.9565	7.61E-05	7.19E-04
ENSBTAG00000019044	762.2852	-1.7648	0.4787	-3.6866	2.27E-04	1.87E-03
ENSBTAG00000017330	908.5951	-1.7661	0.5068	-3.4845	4.93E-04	3.61E-03

ENSBTAG00000014809	7609.9970	-1.7690	0.5618	-3.1487	1.64E-03	9.69E-03
ENSBTAG00000002910	114.2182	-1.7744	0.4453	-3.9852	6.74E-05	6.51E-04
ENSBTAG00000000668	273.4894	-1.7751	0.4587	-3.8701	1.09E-04	9.90E-04
ENSBTAG00000019434	1138.6112	-1.7846	0.5381	-3.3167	9.11E-04	6.00E-03
ENSBTAG00000015426	1444.8664	-1.7889	0.3834	-4.6656	3.08E-06	4.19E-05
ENSBTAG00000009037	171.3006	-1.7913	0.5395	-3.3204	8.99E-04	5.94E-03
ENSBTAG00000015085	154.7108	-1.7918	0.4895	-3.6605	2.52E-04	2.03E-03
ENSBTAG00000006832	575.1347	-1.7927	0.5245	-3.4175	6.32E-04	4.46E-03
ENSBTAG00000007835	6228.1835	-1.8009	0.3463	-5.1999	1.99E-07	3.65E-06
ENSBTAG00000004799	2816.7471	-1.8046	0.3140	-5.7462	9.13E-09	2.13E-07
ENSBTAG00000011917	365.8020	-1.8071	0.2846	-6.3499	2.15E-10	6.87E-09
ENSBTAG00000001748	103.8145	-1.8122	0.4229	-4.2847	1.83E-05	2.07E-04
ENSBTAG00000048296	447.7982	-1.8138	0.3327	-5.4509	5.01E-08	1.01E-06
ENSBTAG00000019887	182.1528	-1.8199	0.4347	-4.1871	2.83E-05	3.04E-04
ENSBTAG00000015692	89.9662	-1.8247	0.5500	-3.3179	9.07E-04	5.98E-03
ENSBTAG00000020489	2216.7872	-1.8258	0.3464	-5.2704	1.36E-07	2.57E-06
ENSBTAG00000021156	1532.9778	-1.8370	0.2835	-6.4795	9.20E-11	3.09E-09
ENSBTAG00000032148	795.1635	-1.8374	0.4328	-4.2452	2.18E-05	2.42E-04
ENSBTAG00000015142	1096.8529	-1.8438	0.2700	-6.8297	8.51E-12	3.39E-10
ENSBTAG00000003467	613.2450	-1.8479	0.3174	-5.8220	5.82E-09	1.39E-07
ENSBTAG00000011027	2610.2796	-1.8510	0.5427	-3.4109	6.48E-04	4.55E-03
ENSBTAG00000004772	78.0296	-1.8517	0.5064	-3.6568	2.55E-04	2.06E-03
ENSBTAG00000004498	151.0205	-1.8541	0.5612	-3.3037	9.54E-04	6.23E-03
ENSBTAG00000009372	826.7069	-1.8542	0.2914	-6.3632	1.98E-10	6.34E-09
ENSBTAG00000018474	1249.9333	-1.8547	0.4693	-3.9522	7.74E-05	7.30E-04
ENSBTAG00000010389	5751.7435	-1.8621	0.4249	-4.3821	1.18E-05	1.40E-04
ENSBTAG00000007983	61.1878	-1.8640	0.5843	-3.1901	1.42E-03	8.62E-03
ENSBTAG00000019194	142.7150	-1.8695	0.4939	-3.7849	1.54E-04	1.34E-03
ENSBTAG00000018935	314.9975	-1.8719	0.4838	-3.8689	1.09E-04	9.93E-04
ENSBTAG00000012106	184.3100	-1.8760	0.4834	-3.8811	1.04E-04	9.49E-04
ENSBTAG00000000305	1062.6015	-1.8817	0.3678	-5.1169	3.11E-07	5.41E-06
ENSBTAG00000002145	413.0446	-1.8819	0.4432	-4.2458	2.18E-05	2.42E-04
ENSBTAG00000004722	86.4818	-1.8839	0.4429	-4.2535	2.10E-05	2.35E-04
ENSBTAG00000002590	6973.8187	-1.8851	0.4231	-4.4553	8.38E-06	1.03E-04
ENSBTAG00000009287	1041.9418	-1.8856	0.3667	-5.1418	2.72E-07	4.81E-06
ENSBTAG00000038181	777.8345	-1.8885	0.3138	-6.0191	1.75E-09	4.69E-08
ENSBTAG00000001652	1784.5754	-1.8901	0.3729	-5.0694	3.99E-07	6.77E-06
ENSBTAG00000000843	1180.0922	-1.8936	0.2832	-6.6862	2.29E-11	8.51E-10
ENSBTAG00000014564	218.4932	-1.8978	0.4668	-4.0654	4.79E-05	4.80E-04
ENSBTAG00000005574	4806.9965	-1.8982	0.5810	-3.2671	1.09E-03	6.92E-03
ENSBTAG00000015968	348.1743	-1.8986	0.3837	-4.9484	7.48E-07	1.18E-05
ENSBTAG00000017922	428.2921	-1.9018	0.3939	-4.8280	1.38E-06	2.05E-05
ENSBTAG00000015505	3615.2727	-1.9077	0.4015	-4.7516	2.02E-06	2.87E-05

ENSBTAG00000004364	90.9334	-1.9084	0.6000	-3.1809	1.47E-03	8.85E-03
ENSBTAG00000001752	135.2729	-1.9129	0.3777	-5.0641	4.10E-07	6.94E-06
ENSBTAG00000006528	114.6997	-1.9194	0.5893	-3.2569	1.13E-03	7.11E-03
ENSBTAG00000001694	1278.5770	-1.9208	0.5678	-3.3832	7.16E-04	4.96E-03
ENSBTAG00000013688	764.5016	-1.9225	0.3979	-4.8313	1.36E-06	2.03E-05
ENSBTAG00000004277	675.8960	-1.9252	0.5170	-3.7241	1.96E-04	1.65E-03
ENSBTAG00000033679	207.5312	-1.9263	0.3130	-6.1551	7.50E-10	2.12E-08
ENSBTAG00000012725	744.8440	-1.9310	0.5786	-3.3372	8.46E-04	5.68E-03
ENSBTAG00000034963	87.8160	-1.9334	0.4970	-3.8904	1.00E-04	9.19E-04
ENSBTAG00000025496	121.6029	-1.9416	0.5516	-3.5198	4.32E-04	3.22E-03
ENSBTAG00000005596	2692.3964	-1.9463	0.4742	-4.1047	4.05E-05	4.16E-04
ENSBTAG00000037729	63.0495	-1.9521	0.6201	-3.1483	1.64E-03	9.70E-03
ENSBTAG00000037962	164.9769	-1.9551	0.6120	-3.1949	1.40E-03	8.53E-03
ENSBTAG00000012629	533.5800	-1.9557	0.4045	-4.8349	1.33E-06	2.00E-05
ENSBTAG00000018394	190.6029	-1.9561	0.6040	-3.2388	1.20E-03	7.51E-03
ENSBTAG00000018638	70.7171	-1.9587	0.5950	-3.2921	9.95E-04	6.44E-03
ENSBTAG00000021072	137.5696	-1.9612	0.4103	-4.7804	1.75E-06	2.53E-05
ENSBTAG00000008433	501.3435	-1.9672	0.2440	-8.0636	7.41E-16	5.04E-14
ENSBTAG00000017685	7026.1447	-1.9764	0.4293	-4.6043	4.14E-06	5.48E-05
ENSBTAG00000019421	252.8585	-1.9845	0.5040	-3.9372	8.24E-05	7.71E-04
ENSBTAG00000016757	4110.9970	-1.9902	0.5244	-3.7949	1.48E-04	1.29E-03
ENSBTAG00000038540	127.8077	-1.9967	0.6145	-3.2493	1.16E-03	7.29E-03
ENSBTAG00000014649	869.3397	-2.0075	0.2942	-6.8238	8.86E-12	3.53E-10
ENSBTAG00000014103	331.1418	-2.0083	0.5003	-4.0142	5.96E-05	5.85E-04
ENSBTAG00000000243	1310.0085	-2.0084	0.4275	-4.6975	2.63E-06	3.65E-05
ENSBTAG00000031439	213.0605	-2.0094	0.4853	-4.1407	3.46E-05	3.63E-04
ENSBTAG00000005565	520.2784	-2.0107	0.6212	-3.2370	1.21E-03	7.55E-03
ENSBTAG00000024958	1435.0124	-2.0173	0.4450	-4.5329	5.82E-06	7.40E-05
ENSBTAG00000010967	51.7645	-2.0189	0.4976	-4.0572	4.97E-05	4.95E-04
ENSBTAG00000018410	60.0138	-2.0307	0.5906	-3.4386	5.85E-04	4.19E-03
ENSBTAG00000034867	408.2368	-2.0316	0.3397	-5.9802	2.23E-09	5.80E-08
ENSBTAG00000010541	63.2677	-2.0316	0.5980	-3.3973	6.81E-04	4.74E-03
ENSBTAG00000000286	993.0973	-2.0324	0.3332	-6.0995	1.06E-09	2.93E-08
ENSBTAG00000003069	262.1452	-2.0326	0.6359	-3.1966	1.39E-03	8.50E-03
ENSBTAG00000030369	287.6425	-2.0371	0.3445	-5.9137	3.35E-09	8.37E-08
ENSBTAG00000010356	4841.1873	-2.0413	0.3141	-6.4986	8.11E-11	2.75E-09
ENSBTAG00000005340	197.3180	-2.0436	0.4445	-4.5977	4.27E-06	5.63E-05
ENSBTAG00000046823	1742.9508	-2.0444	0.2257	-9.0600	1.30E-19	1.41E-17
ENSBTAG00000000146	649.6227	-2.0463	0.2941	-6.9582	3.45E-12	1.43E-10
ENSBTAG00000004653	15982.4627	-2.0489	0.4645	-4.4108	1.03E-05	1.25E-04
ENSBTAG00000011810	629.4284	-2.0533	0.6420	-3.1982	1.38E-03	8.48E-03
ENSBTAG00000013048	794.5911	-2.0565	0.2587	-7.9507	1.85E-15	1.23E-13
ENSBTAG00000013929	1220.6838	-2.0567	0.4300	-4.7836	1.72E-06	2.50E-05

ENSBTAG00000016544	74.1325	-2.0569	0.5546	-3.7090	2.08E-04	1.73E-03
ENSBTAG00000015335	226.5362	-2.0579	0.4275	-4.8134	1.48E-06	2.19E-05
ENSBTAG00000019373	2138.0613	-2.0598	0.2504	-8.2275	1.91E-16	1.40E-14
ENSBTAG00000010976	726.6403	-2.0617	0.3695	-5.5797	2.41E-08	5.10E-07
ENSBTAG00000005356	4105.5297	-2.0628	0.4803	-4.2943	1.75E-05	2.00E-04
ENSBTAG00000001473	69.4825	-2.0629	0.6509	-3.1696	1.53E-03	9.13E-03
ENSBTAG00000010644	4815.3081	-2.0654	0.5788	-3.5683	3.59E-04	2.75E-03
ENSBTAG00000012342	1113.6969	-2.0673	0.5101	-4.0524	5.07E-05	5.04E-04
ENSBTAG00000008895	240.6606	-2.0700	0.4264	-4.8542	1.21E-06	1.84E-05
ENSBTAG00000004617	349.5694	-2.0715	0.4822	-4.2960	1.74E-05	1.98E-04
ENSBTAG00000020844	964.9914	-2.0794	0.4183	-4.9712	6.65E-07	1.06E-05
ENSBTAG00000001754	832.9408	-2.0795	0.3211	-6.4758	9.43E-11	3.15E-09
ENSBTAG00000044112	432.1433	-2.0860	0.4231	-4.9301	8.22E-07	1.29E-05
ENSBTAG00000020060	11901.7900	-2.0935	0.4162	-5.0304	4.89E-07	8.06E-06
ENSBTAG00000021636	266.7854	-2.0966	0.6433	-3.2591	1.12E-03	7.07E-03
ENSBTAG00000019024	559.2832	-2.1033	0.2553	-8.2394	1.73E-16	1.29E-14
ENSBTAG00000015513	2334.0561	-2.1081	0.4617	-4.5660	4.97E-06	6.44E-05
ENSBTAG00000011338	600.3545	-2.1177	0.3415	-6.2014	5.60E-10	1.62E-08
ENSBTAG00000033174	83.6420	-2.1178	0.6724	-3.1495	1.64E-03	9.68E-03
ENSBTAG00000013224	160.8469	-2.1188	0.4310	-4.9162	8.83E-07	1.38E-05
ENSBTAG00000009998	335.1078	-2.1219	0.6413	-3.3084	9.38E-04	6.15E-03
ENSBTAG00000015794	1827.4680	-2.1241	0.6680	-3.1799	1.47E-03	8.87E-03
ENSBTAG00000002817	201.1909	-2.1256	0.5964	-3.5639	3.65E-04	2.79E-03
ENSBTAG00000039789	53.0837	-2.1296	0.6445	-3.3044	9.52E-04	6.22E-03
ENSBTAG00000012907	3200.0388	-2.1336	0.3584	-5.9526	2.64E-09	6.77E-08
ENSBTAG00000000120	290.0377	-2.1404	0.4231	-5.0584	4.23E-07	7.13E-06
ENSBTAG00000021934	575.4382	-2.1691	0.2556	-8.4865	2.13E-17	1.78E-15
ENSBTAG00000007281	337.1625	-2.1773	0.5493	-3.9636	7.38E-05	7.03E-04
ENSBTAG00000006894	3926.8669	-2.1926	0.6900	-3.1779	1.48E-03	8.91E-03
ENSBTAG00000020934	100.3294	-2.1952	0.6869	-3.1956	1.40E-03	8.52E-03
ENSBTAG00000007796	63.8095	-2.2001	0.5087	-4.3250	1.52E-05	1.77E-04
ENSBTAG00000016197	988.7269	-2.2055	0.2825	-7.8063	5.89E-15	3.59E-13
ENSBTAG00000003957	47.5777	-2.2058	0.6966	-3.1666	1.54E-03	9.21E-03
ENSBTAG00000010745	407.2490	-2.2196	0.4814	-4.6105	4.02E-06	5.34E-05
ENSBTAG00000021252	277.2062	-2.2327	0.2995	-7.4548	9.00E-14	4.68E-12
ENSBTAG00000012510	1042.2395	-2.2335	0.3214	-6.9502	3.65E-12	1.50E-10
ENSBTAG00000015534	399.5722	-2.2415	0.3955	-5.6670	1.45E-08	3.22E-07
ENSBTAG00000006349	72.4605	-2.2474	0.5492	-4.0923	4.27E-05	4.36E-04
ENSBTAG00000017373	1083.6921	-2.2487	0.2996	-7.5069	6.05E-14	3.23E-12
ENSBTAG00000001417	242.9812	-2.2491	0.6729	-3.3422	8.31E-04	5.60E-03
ENSBTAG00000021811	1427.9031	-2.2522	0.4529	-4.9730	6.59E-07	1.05E-05
ENSBTAG00000019550	118.3708	-2.2538	0.3961	-5.6901	1.27E-08	2.85E-07
ENSBTAG00000023374	899.6741	-2.2673	0.5351	-4.2372	2.26E-05	2.49E-04

ENSBTAG00000031352	30.2441	-2.2680	0.7022	-3.2297	1.24E-03	7.71E-03
ENSBTAG00000014506	299.1410	-2.2681	0.4546	-4.9897	6.05E-07	9.80E-06
ENSBTAG00000000188	95.6480	-2.2688	0.7016	-3.2339	1.22E-03	7.62E-03
ENSBTAG00000022751	36.3743	-2.2746	0.6881	-3.3054	9.48E-04	6.20E-03
ENSBTAG00000047357	74.7523	-2.2805	0.6813	-3.3471	8.17E-04	5.52E-03
ENSBTAG00000016194	124.5236	-2.2805	0.5944	-3.8365	1.25E-04	1.11E-03
ENSBTAG00000021991	255.6433	-2.2901	0.5199	-4.4050	1.06E-05	1.28E-04
ENSBTAG00000004954	1615.9688	-2.2934	0.3423	-6.6998	2.09E-11	7.82E-10
ENSBTAG00000019621	3836.6449	-2.2935	0.2333	-9.8305	8.32E-23	1.46E-20
ENSBTAG00000012262	692.3407	-2.3008	0.6340	-3.6289	2.85E-04	2.25E-03
ENSBTAG00000018206	228.5276	-2.3020	0.5946	-3.8712	1.08E-04	9.87E-04
ENSBTAG00000007121	1738.9332	-2.3029	0.5487	-4.1971	2.70E-05	2.92E-04
ENSBTAG00000007031	295.2074	-2.3076	0.4679	-4.9319	8.14E-07	1.28E-05
ENSBTAG00000009545	93.8533	-2.3253	0.6896	-3.3718	7.47E-04	5.14E-03
ENSBTAG00000016549	778.5831	-2.3405	0.3944	-5.9339	2.96E-09	7.53E-08
ENSBTAG00000010278	92.2219	-2.3522	0.6909	-3.4046	6.63E-04	4.64E-03
ENSBTAG00000032331	389.7828	-2.3526	0.4807	-4.8945	9.86E-07	1.53E-05
ENSBTAG00000020415	74.1018	-2.3600	0.5147	-4.5854	4.53E-06	5.94E-05
ENSBTAG00000008576	72.4830	-2.3628	0.5121	-4.6137	3.96E-06	5.27E-05
ENSBTAG00000008464	58.8833	-2.3630	0.6255	-3.7776	1.58E-04	1.37E-03
ENSBTAG00000031737	78.0526	-2.3674	0.4975	-4.7582	1.95E-06	2.78E-05
ENSBTAG00000016045	32442.1320	-2.3902	0.4096	-5.8358	5.35E-09	1.28E-07
ENSBTAG00000019630	290.4017	-2.3914	0.5828	-4.1036	4.07E-05	4.17E-04
ENSBTAG00000013856	113.2168	-2.3932	0.7298	-3.2791	1.04E-03	6.69E-03
ENSBTAG00000034626	23.1579	-2.4083	0.7598	-3.1695	1.53E-03	9.13E-03
ENSBTAG00000043985	512.7240	-2.4106	0.4008	-6.0149	1.80E-09	4.80E-08
ENSBTAG00000014124	253.8794	-2.4130	0.4734	-5.0968	3.45E-07	5.93E-06
ENSBTAG00000032642	415.3050	-2.4479	0.7714	-3.1733	1.51E-03	9.03E-03
ENSBTAG00000038865	1107.5211	-2.4481	0.4811	-5.0888	3.60E-07	6.16E-06
ENSBTAG00000020040	416.8064	-2.4494	0.4445	-5.5108	3.57E-08	7.33E-07
ENSBTAG00000008467	71.2521	-2.4567	0.6797	-3.6142	3.01E-04	2.36E-03
ENSBTAG00000012319	688.1951	-2.4621	0.6783	-3.6299	2.84E-04	2.24E-03
ENSBTAG00000011076	106.7901	-2.4641	0.7800	-3.1592	1.58E-03	9.40E-03
ENSBTAG00000009830	810.9596	-2.4760	0.6018	-4.1147	3.88E-05	4.00E-04
ENSBTAG00000003474	1845.9323	-2.4825	0.4837	-5.1325	2.86E-07	5.05E-06
ENSBTAG00000004732	55.1517	-2.4906	0.7194	-3.4619	5.36E-04	3.89E-03
ENSBTAG00000019658	223.8922	-2.4953	0.7291	-3.4224	6.21E-04	4.40E-03
ENSBTAG00000003634	317.8566	-2.5037	0.3085	-8.1156	4.83E-16	3.39E-14
ENSBTAG00000040298	389.9707	-2.5094	0.4141	-6.0602	1.36E-09	3.69E-08
ENSBTAG00000009065	3157.0752	-2.5095	0.3702	-6.7780	1.22E-11	4.76E-10
ENSBTAG00000006227	5623.3444	-2.5107	0.4832	-5.1960	2.04E-07	3.70E-06
ENSBTAG00000000153	682.8749	-2.5174	0.6159	-4.0873	4.36E-05	4.44E-04
ENSBTAG00000004542	6283.3443	-2.5193	0.6767	-3.7227	1.97E-04	1.65E-03

ENSBTAG00000014249	17708.1531	-2.5385	0.6212	-4.0867	4.38E-05	4.45E-04
ENSBTAG00000004078	323.3808	-2.5433	0.6590	-3.8593	1.14E-04	1.03E-03
ENSBTAG00000018530	51.9997	-2.5488	0.5967	-4.2716	1.94E-05	2.18E-04
ENSBTAG00000021025	1144.6834	-2.5561	0.4374	-5.8444	5.08E-09	1.23E-07
ENSBTAG00000017823	29.0839	-2.5620	0.7637	-3.3546	7.95E-04	5.41E-03
ENSBTAG00000005034	154.3475	-2.5739	0.4483	-5.7417	9.37E-09	2.17E-07
ENSBTAG00000013303	1279.2721	-2.5819	0.3037	-8.5004	1.89E-17	1.61E-15
ENSBTAG00000019138	294.9387	-2.5823	0.3673	-7.0303	2.06E-12	8.88E-11
ENSBTAG00000020205	761.6081	-2.5871	0.4250	-6.0870	1.15E-09	3.15E-08
ENSBTAG00000047536	73.1767	-2.5917	0.7706	-3.3630	7.71E-04	5.28E-03
ENSBTAG00000017564	1470.3434	-2.5917	0.2763	-9.3800	6.60E-21	9.17E-19
ENSBTAG00000014899	110.8708	-2.5996	0.5931	-4.3828	1.17E-05	1.40E-04
ENSBTAG00000010198	222.8214	-2.6066	0.7057	-3.6936	2.21E-04	1.82E-03
ENSBTAG00000044061	32.4134	-2.6072	0.7720	-3.3772	7.32E-04	5.06E-03
ENSBTAG00000012972	578.7218	-2.6206	0.4267	-6.1422	8.14E-10	2.28E-08
ENSBTAG00000011660	85.1898	-2.6355	0.7854	-3.3558	7.91E-04	5.40E-03
ENSBTAG00000003986	1464.8398	-2.6481	0.3931	-6.7359	1.63E-11	6.19E-10
ENSBTAG00000008739	92.4617	-2.6495	0.7567	-3.5012	4.63E-04	3.41E-03
ENSBTAG00000006515	37.3497	-2.6694	0.8025	-3.3262	8.80E-04	5.84E-03
ENSBTAG00000017540	269.3464	-2.6784	0.5798	-4.6196	3.84E-06	5.13E-05
ENSBTAG00000046408	124.0855	-2.6832	0.6102	-4.3975	1.09E-05	1.32E-04
ENSBTAG00000020551	5234.7881	-2.6833	0.4371	-6.1389	8.31E-10	2.31E-08
ENSBTAG00000033367	38.4144	-2.7017	0.7752	-3.4852	4.92E-04	3.60E-03
ENSBTAG00000014324	2774.6786	-2.7084	0.4682	-5.7844	7.28E-09	1.72E-07
ENSBTAG00000009029	231.0740	-2.7101	0.5588	-4.8500	1.23E-06	1.87E-05
ENSBTAG00000010360	470.3733	-2.7247	0.5118	-5.3235	1.02E-07	1.97E-06
ENSBTAG00000047670	3959.3648	-2.7265	0.6042	-4.5126	6.40E-06	8.09E-05
ENSBTAG00000016255	164.7976	-2.7456	0.3921	-7.0014	2.53E-12	1.07E-10
ENSBTAG00000011525	212.8660	-2.7497	0.4338	-6.3393	2.31E-10	7.32E-09
ENSBTAG00000011187	1687.9778	-2.7516	0.4639	-5.9313	3.01E-09	7.63E-08
ENSBTAG00000010999	70.2053	-2.7906	0.5729	-4.8713	1.11E-06	1.71E-05
ENSBTAG00000003754	353.6063	-2.8064	0.4502	-6.2330	4.58E-10	1.35E-08
ENSBTAG00000003779	192.6689	-2.8096	0.4001	-7.0216	2.19E-12	9.41E-11
ENSBTAG00000015656	73.9847	-2.8356	0.7236	-3.9187	8.90E-05	8.27E-04
ENSBTAG00000015345	43.8357	-2.8650	0.8173	-3.5054	4.56E-04	3.38E-03
ENSBTAG00000001609	19.2253	-2.8720	0.8732	-3.2890	1.01E-03	6.49E-03
ENSBTAG00000015735	215.3120	-2.8723	0.4381	-6.5563	5.51E-11	1.92E-09
ENSBTAG00000009337	384.5463	-2.8752	0.4900	-5.8675	4.42E-09	1.08E-07
ENSBTAG00000047216	79.9696	-2.8753	0.5947	-4.8352	1.33E-06	1.99E-05
ENSBTAG00000012307	775.9691	-2.8819	0.5417	-5.3206	1.03E-07	1.99E-06
ENSBTAG00000010581	151.7561	-2.8873	0.8092	-3.5681	3.60E-04	2.75E-03
ENSBTAG00000030686	345.8703	-2.8994	0.4949	-5.8589	4.66E-09	1.13E-07
ENSBTAG00000046017	315.9154	-2.9081	0.5368	-5.4175	6.04E-08	1.20E-06

ENSBTAG00000027426	456.6499	-2.9306	0.8716	-3.3623	7.73E-04	5.29E-03
ENSBTAG00000019453	89.5162	-2.9367	0.7337	-4.0025	6.27E-05	6.09E-04
ENSBTAG00000000245	103.9015	-2.9444	0.8002	-3.6798	2.33E-04	1.91E-03
ENSBTAG00000002081	4485.5274	-2.9537	0.5520	-5.3508	8.76E-08	1.72E-06
ENSBTAG00000002914	501.7634	-2.9768	0.7174	-4.1495	3.33E-05	3.52E-04
ENSBTAG00000015108	1018.7504	-2.9957	0.2628	-11.3993	4.22E-30	1.11E-27
ENSBTAG00000011838	1060.5557	-3.0062	0.4823	-6.2327	4.58E-10	1.35E-08
ENSBTAG00000019164	656.8833	-3.0208	0.5408	-5.5862	2.32E-08	4.94E-07
ENSBTAG00000007374	1687.4152	-3.0308	0.4050	-7.4828	7.28E-14	3.85E-12
ENSBTAG00000014001	2709.3616	-3.0337	0.3919	-7.7414	9.83E-15	5.79E-13
ENSBTAG00000009362	4117.7047	-3.0345	0.5442	-5.5757	2.47E-08	5.20E-07
ENSBTAG00000017616	622.1242	-3.0601	0.8723	-3.5082	4.51E-04	3.35E-03
ENSBTAG00000006161	153.2699	-3.0626	0.5931	-5.1640	2.42E-07	4.34E-06
ENSBTAG00000013596	138.0049	-3.0707	0.7473	-4.1088	3.98E-05	4.09E-04
ENSBTAG00000018046	3984.9821	-3.0712	0.4454	-6.8950	5.39E-12	2.20E-10
ENSBTAG00000007125	57.9615	-3.0871	0.7605	-4.0590	4.93E-05	4.93E-04
ENSBTAG00000025458	100.4196	-3.0946	0.7008	-4.4160	1.01E-05	1.22E-04
ENSBTAG00000030227	389.6385	-3.1056	0.4219	-7.3606	1.83E-13	9.11E-12
ENSBTAG00000000875	237.5543	-3.1078	0.3718	-8.3597	6.29E-17	5.04E-15
ENSBTAG00000002084	98.6722	-3.1089	0.7624	-4.0779	4.55E-05	4.59E-04
ENSBTAG00000000672	2241.5783	-3.1151	0.4570	-6.8163	9.34E-12	3.69E-10
ENSBTAG00000006403	60.6880	-3.1181	0.8742	-3.5668	3.61E-04	2.76E-03
ENSBTAG00000006385	105.9428	-3.1310	0.9186	-3.4084	6.53E-04	4.58E-03
ENSBTAG00000018431	219.4044	-3.1407	0.5135	-6.1169	9.54E-10	2.64E-08
ENSBTAG00000021273	558.0952	-3.1478	0.4512	-6.9766	3.03E-12	1.27E-10
ENSBTAG00000000124	198.6709	-3.1730	0.7735	-4.1023	4.09E-05	4.19E-04
ENSBTAG00000007615	23.6992	-3.1730	0.9936	-3.1935	1.41E-03	8.55E-03
ENSBTAG00000010989	3740.0553	-3.1774	0.3587	-8.8588	8.09E-19	8.02E-17
ENSBTAG00000004751	25.2670	-3.1966	0.9546	-3.3488	8.12E-04	5.50E-03
ENSBTAG00000034776	2338.1911	-3.2182	0.4096	-7.8561	3.96E-15	2.53E-13
ENSBTAG00000015988	1307.2099	-3.2544	0.8766	-3.7125	2.05E-04	1.71E-03
ENSBTAG00000021420	342.6957	-3.2586	0.4643	-7.0189	2.24E-12	9.54E-11
ENSBTAG00000022799	2129.2403	-3.2807	0.6812	-4.8160	1.46E-06	2.17E-05
ENSBTAG00000019722	88.7827	-3.3261	0.6020	-5.5253	3.29E-08	6.80E-07
ENSBTAG00000014259	204.2966	-3.3325	0.3625	-9.1918	3.86E-20	4.49E-18
ENSBTAG00000020199	3010.9563	-3.3799	0.3503	-9.6490	4.97E-22	7.79E-20
ENSBTAG00000018524	162.5213	-3.3799	0.3613	-9.3547	8.38E-21	1.14E-18
ENSBTAG00000017031	147.7341	-3.3823	0.7293	-4.6376	3.53E-06	4.75E-05
ENSBTAG00000014956	3950.9650	-3.3964	0.4326	-7.8510	4.13E-15	2.60E-13
ENSBTAG00000004591	164.3297	-3.4003	0.6657	-5.1082	3.25E-07	5.63E-06
ENSBTAG00000006769	18218.2151	-3.4315	0.7156	-4.7956	1.62E-06	2.37E-05
ENSBTAG00000046803	135.7843	-3.4582	0.9404	-3.6775	2.36E-04	1.93E-03
ENSBTAG00000005475	67.7211	-3.4698	0.8525	-4.0702	4.70E-05	4.72E-04

ENSBTAG00000009194	236.4377	-3.4729	0.3649	-9.5168	1.79E-21	2.68E-19
ENSBTAG00000017452	443.3350	-3.4890	0.3842	-9.0806	1.08E-19	1.17E-17
ENSBTAG00000005729	129.4857	-3.5005	0.6115	-5.7247	1.04E-08	2.38E-07
ENSBTAG00000016918	5600.5397	-3.5189	0.4494	-7.8307	4.85E-15	3.02E-13
ENSBTAG00000007827	26.4229	-3.5549	0.8895	-3.9966	6.43E-05	6.22E-04
ENSBTAG00000015329	1665.4706	-3.5799	0.3858	-9.2791	1.71E-20	2.11E-18
ENSBTAG00000010362	1226.5618	-3.5954	0.4072	-8.8288	1.06E-18	1.02E-16
ENSBTAG00000037580	22.0438	-3.6012	0.9573	-3.7619	1.69E-04	1.45E-03
ENSBTAG00000022020	64.5044	-3.6094	1.0504	-3.4362	5.90E-04	4.22E-03
ENSBTAG00000016220	358.5289	-3.7164	0.4882	-7.6128	2.68E-14	1.50E-12
ENSBTAG00000019473	67.2030	-3.7438	0.8628	-4.3389	1.43E-05	1.68E-04
ENSBTAG00000013492	212.1435	-3.7811	0.8462	-4.4686	7.88E-06	9.78E-05
ENSBTAG00000003910	19.2521	-3.7822	0.9973	-3.7925	1.49E-04	1.30E-03
ENSBTAG00000048304	20.2137	-3.8083	0.8987	-4.2375	2.26E-05	2.49E-04
ENSBTAG00000032548	57.1963	-3.8439	0.7717	-4.9808	6.33E-07	1.02E-05
ENSBTAG00000021880	110.5334	-3.9027	0.9939	-3.9266	8.61E-05	8.03E-04
ENSBTAG00000032077	144.8869	-3.9096	0.9106	-4.2934	1.76E-05	2.00E-04
ENSBTAG00000013801	112.1205	-3.9435	0.7095	-5.5586	2.72E-08	5.69E-07
ENSBTAG00000019866	7129.0681	-3.9510	0.7564	-5.2233	1.76E-07	3.25E-06
ENSBTAG00000007253	473.2857	-3.9800	0.5564	-7.1526	8.51E-13	3.88E-11
ENSBTAG00000026156	67.4961	-4.0118	0.9828	-4.0820	4.46E-05	4.53E-04
ENSBTAG00000045678	50.6366	-4.0308	1.1248	-3.5835	3.39E-04	2.61E-03
ENSBTAG00000000804	510.8196	-4.0562	0.6481	-6.2582	3.89E-10	1.17E-08
ENSBTAG00000031458	39.7929	-4.0569	1.0743	-3.7763	1.59E-04	1.37E-03
ENSBTAG00000002025	1542.3201	-4.0606	0.3089	-13.1470	1.77E-39	7.96E-37
ENSBTAG00000013416	142.2015	-4.0987	0.9676	-4.2360	2.28E-05	2.50E-04
ENSBTAG00000010070	14.5324	-4.1023	1.0387	-3.9495	7.83E-05	7.38E-04
ENSBTAG00000020018	18.1982	-4.1272	1.1113	-3.7140	2.04E-04	1.70E-03
ENSBTAG00000016741	597.0625	-4.1639	0.4467	-9.3215	1.15E-20	1.52E-18
ENSBTAG00000016732	156.2327	-4.1769	0.5065	-8.2468	1.63E-16	1.22E-14
ENSBTAG00000027075	334.9927	-4.1951	0.9759	-4.2988	1.72E-05	1.97E-04
ENSBTAG00000019065	291.9212	-4.2218	0.6438	-6.5578	5.46E-11	1.90E-09
ENSBTAG00000011246	123.8375	-4.2480	0.5767	-7.3658	1.76E-13	8.83E-12
ENSBTAG00000017866	1727.2943	-4.3079	0.9027	-4.7725	1.82E-06	2.61E-05
ENSBTAG00000003319	1723.7719	-4.3464	0.4855	-8.9526	3.47E-19	3.61E-17
ENSBTAG00000032844	1053.9122	-4.3661	0.6733	-6.4846	8.90E-11	2.99E-09
ENSBTAG00000003667	325.1328	-4.4668	0.5349	-8.3511	6.76E-17	5.36E-15
ENSBTAG00000020793	12.3443	-4.5005	1.1365	-3.9601	7.49E-05	7.11E-04
ENSBTAG00000017529	723.1021	-4.5514	0.3718	-12.2421	1.85E-34	6.40E-32
ENSBTAG00000011224	3376.2845	-4.5603	0.5294	-8.6135	7.09E-18	6.25E-16
ENSBTAG00000021189	673.1758	-4.6401	0.8770	-5.2909	1.22E-07	2.31E-06
ENSBTAG00000001783	156.0889	-4.6608	0.6068	-7.6812	1.58E-14	8.99E-13
ENSBTAG00000002151	80.6374	-4.8078	1.0712	-4.4884	7.18E-06	8.99E-05

ENSBTAG00000002983	14.4071	-4.8131	1.1091	-4.3397	1.43E-05	1.68E-04
ENSBTAG00000011461	27.5052	-4.8283	0.9149	-5.2774	1.31E-07	2.48E-06
ENSBTAG00000004624	110.6526	-4.9494	0.9935	-4.9820	6.29E-07	1.01E-05
ENSBTAG00000011381	37.3889	-4.9528	0.9072	-5.4592	4.78E-08	9.67E-07
ENSBTAG00000009725	795.0144	-5.1560	0.7154	-7.2070	5.72E-13	2.71E-11
ENSBTAG00000016754	17.4178	-5.1909	1.2014	-4.3208	1.55E-05	1.80E-04
ENSBTAG00000006231	725.1027	-5.1938	0.3858	-13.4623	2.61E-41	1.26E-38
ENSBTAG00000043948	189.4062	-5.2532	1.0109	-5.1964	2.03E-07	3.70E-06
ENSBTAG00000019052	1179.5021	-5.3444	0.8451	-6.3243	2.54E-10	8.00E-09
ENSBTAG00000021101	165.5768	-5.3838	0.8243	-6.5311	6.53E-11	2.24E-09
ENSBTAG00000007633	74.0183	-5.3909	0.9919	-5.4346	5.49E-08	1.10E-06
ENSBTAG00000003137	891.1959	-5.4487	0.5952	-9.1541	5.48E-20	6.26E-18
ENSBTAG00000044123	239.8094	-5.5099	0.6820	-8.0789	6.54E-16	4.50E-14
ENSBTAG00000006355	238.3656	-5.6117	0.5385	-10.4202	2.01E-25	4.22E-23
ENSBTAG00000013959	61.2694	-6.4335	0.8075	-7.9675	1.62E-15	1.09E-13
ENSBTAG00000019859	1455.5434	-6.4404	0.7487	-8.6021	7.83E-18	6.81E-16
ENSBTAG00000007816	34.8040	-6.4912	1.1080	-5.8582	4.68E-09	1.13E-07
ENSBTAG00000035654	115.0566	-7.6406	0.7980	-9.5744	1.02E-21	1.55E-19

Supplementary Table 3: List of 285 ERK1/2-dependent DEGs

	PD0325901 + GnRH vs Vehicle + GnRH					
ENSEMBL ID	baseMean	log2FoldChange	lfcSE	stat	pvalue	padj
ENSBTAG00000007820	45.6651	3.2592	0.5605	5.8149	6.07E-09	1.47E-06
ENSBTAG00000006902	198.4353	2.5489	0.4701	5.4222	5.89E-08	1.12E-05
ENSBTAG00000022783	103.1593	2.4955	0.4260	5.8577	4.69E-09	1.16E-06
ENSBTAG00000007316	52.3715	2.2861	0.4932	4.6353	3.56E-06	3.82E-04
ENSBTAG00000035654	61.2380	2.2632	0.5705	3.9670	7.28E-05	4.50E-03
ENSBTAG00000009725	281.1559	2.2086	0.5581	3.9576	7.57E-05	4.62E-03
ENSBTAG00000024311	21.3977	2.1614	0.5579	3.8744	1.07E-04	6.03E-03
ENSBTAG00000040386	1133.3815	2.0565	0.5327	3.8607	1.13E-04	6.26E-03
ENSBTAG00000017136	33.5374	2.0128	0.5250	3.8338	1.26E-04	6.66E-03
ENSBTAG00000038437	440.1741	1.9070	0.5026	3.7946	1.48E-04	7.44E-03
ENSBTAG00000006894	3769.2844	1.8201	0.3596	5.0614	4.16E-07	5.73E-05
ENSBTAG00000022613	21982.5177	1.7896	0.3121	5.7344	9.79E-09	2.29E-06
ENSBTAG00000012919	1847.8383	1.7414	0.3985	4.3699	1.24E-05	1.10E-03
ENSBTAG00000021963	1598.7590	1.7048	0.3009	5.6663	1.46E-08	3.30E-06
ENSBTAG00000014581	88.9310	1.6895	0.4497	3.7569	1.72E-04	8.30E-03
ENSBTAG00000012931	8093.6453	1.6388	0.3323	4.9320	8.14E-07	1.03E-04
ENSBTAG00000019633	398.7565	1.5897	0.3129	5.0808	3.76E-07	5.22E-05
ENSBTAG00000037791	992.3010	1.5702	0.3750	4.1873	2.82E-05	2.13E-03
ENSBTAG00000023372	241.9832	1.5503	0.2459	6.3048	2.88E-10	9.64E-08
ENSBTAG00000018004	1421.0878	1.5148	0.4005	3.7822	1.55E-04	7.74E-03
ENSBTAG00000010498	12856.0180	1.4962	0.4019	3.7228	1.97E-04	9.35E-03
ENSBTAG00000021492	296.9165	1.4732	0.3684	3.9985	6.37E-05	4.00E-03
ENSBTAG00000011460	1291.0142	1.4569	0.3522	4.1363	3.53E-05	2.54E-03
ENSBTAG00000018181	3003.3311	1.4535	0.1886	7.7059	1.30E-14	8.69E-12
ENSBTAG00000020647	419.2874	1.3947	0.3769	3.7007	2.15E-04	9.93E-03
ENSBTAG00000001745	23540.0186	1.3881	0.2439	5.6920	1.26E-08	2.89E-06
ENSBTAG00000004954	1143.3732	1.3262	0.3018	4.3943	1.11E-05	1.00E-03
ENSBTAG00000034222	1637.6656	1.2843	0.2879	4.4606	8.17E-06	7.74E-04
ENSBTAG00000039696	1820.2368	1.2769	0.3176	4.0208	5.80E-05	3.68E-03
ENSBTAG00000016627	305.4061	1.2739	0.3033	4.1999	2.67E-05	2.06E-03
ENSBTAG00000013889	298.6815	1.0870	0.2670	4.0710	4.68E-05	3.13E-03
ENSBTAG00000015107	7375.9786	1.0474	0.1895	5.5270	3.26E-08	6.73E-06
ENSBTAG00000043985	285.4036	1.0394	0.2525	4.1170	3.84E-05	2.69E-03
ENSBTAG00000017763	2518.4658	-1.0321	0.2561	-4.0302	5.57E-05	3.59E-03
ENSBTAG00000001941	317.5292	-1.0637	0.2630	-4.0444	5.25E-05	3.43E-03
ENSBTAG00000009055	1058.5668	-1.0897	0.2699	-4.0375	5.40E-05	3.50E-03
ENSBTAG00000000266	3123.6644	-1.1006	0.2185	-5.0363	4.75E-07	6.35E-05
ENSBTAG00000004211	2116.3581	-1.1007	0.2713	-4.0573	4.96E-05	3.29E-03
ENSBTAG00000004377	1277.8672	-1.1076	0.2757	-4.0171	5.89E-05	3.73E-03

ENSBTAG00000007596	8891.6855	-1.1724	0.2840	-4.1279	3.66E-05	2.59E-03
ENSBTAG00000004322	3890.2670	-1.1885	0.2740	-4.3381	1.44E-05	1.25E-03
ENSBTAG00000021523	7618.2061	-1.1888	0.3093	-3.8436	1.21E-04	6.60E-03
ENSBTAG00000010493	711.6268	-1.1912	0.2940	-4.0511	5.10E-05	3.34E-03
ENSBTAG00000013750	1006.8376	-1.2062	0.3244	-3.7187	2.00E-04	9.43E-03
ENSBTAG00000038428	788.2573	-1.2147	0.3085	-3.9381	8.21E-05	4.89E-03
ENSBTAG00000003012	866.9810	-1.2273	0.2667	-4.6024	4.18E-06	4.34E-04
ENSBTAG00000012818	191.7306	-1.2524	0.3315	-3.7781	1.58E-04	7.79E-03
ENSBTAG00000018082	275.8647	-1.2970	0.3383	-3.8342	1.26E-04	6.66E-03
ENSBTAG00000021717	339.4716	-1.3455	0.2182	-6.1670	6.96E-10	2.12E-07
ENSBTAG00000032369	1740.5139	-1.3636	0.2660	-5.1264	2.95E-07	4.23E-05
ENSBTAG00000005816	646.3575	-1.3686	0.3697	-3.7022	2.14E-04	9.90E-03
ENSBTAG00000009933	349.2805	-1.3827	0.3587	-3.8546	1.16E-04	6.38E-03
ENSBTAG00000008353	1715.0199	-1.3982	0.2590	-5.3981	6.74E-08	1.23E-05
ENSBTAG00000021818	1496.0128	-1.4054	0.3196	-4.3971	1.10E-05	9.94E-04
ENSBTAG00000013745	652.5756	-1.4265	0.3236	-4.4079	1.04E-05	9.58E-04
ENSBTAG00000015212	888.2039	-1.4380	0.2697	-5.3320	9.72E-08	1.70E-05
ENSBTAG00000017745	2572.0794	-1.4590	0.3302	-4.4189	9.92E-06	9.16E-04
ENSBTAG00000007705	135.4888	-1.4649	0.3600	-4.0688	4.72E-05	3.14E-03
ENSBTAG00000000816	1539.3509	-1.4710	0.3935	-3.7380	1.86E-04	8.89E-03
ENSBTAG00000031231	1703.1230	-1.4974	0.4019	-3.7256	1.95E-04	9.27E-03
ENSBTAG00000024648	2184.1692	-1.5109	0.3252	-4.6461	3.38E-06	3.68E-04
ENSBTAG00000001576	85.1045	-1.5252	0.4013	-3.8011	1.44E-04	7.34E-03
ENSBTAG00000003336	139.2104	-1.5253	0.3439	-4.4347	9.22E-06	8.57E-04
ENSBTAG00000007052	157.3967	-1.5454	0.2992	-5.1648	2.41E-07	3.56E-05
ENSBTAG00000016217	1124.6114	-1.5456	0.3691	-4.1879	2.82E-05	2.13E-03
ENSBTAG00000008091	72.2864	-1.5797	0.3720	-4.2459	2.18E-05	1.73E-03
ENSBTAG00000024503	834.6145	-1.5929	0.4234	-3.7623	1.68E-04	8.18E-03
ENSBTAG00000017457	381.4281	-1.6065	0.3680	-4.3655	1.27E-05	1.11E-03
ENSBTAG00000007530	78.0713	-1.6398	0.4193	-3.9109	9.20E-05	5.39E-03
ENSBTAG00000008401	1050.9935	-1.6421	0.3285	-4.9984	5.78E-07	7.58E-05
ENSBTAG00000016061	319.5733	-1.6452	0.4298	-3.8282	1.29E-04	6.74E-03
ENSBTAG00000010152	79.4367	-1.6679	0.4314	-3.8663	1.11E-04	6.21E-03
ENSBTAG00000002624	495.6110	-1.6815	0.4094	-4.1071	4.01E-05	2.78E-03
ENSBTAG00000016354	1117.1802	-1.6836	0.3256	-5.1706	2.33E-07	3.52E-05
ENSBTAG00000006130	334.3320	-1.6901	0.3272	-5.1651	2.40E-07	3.56E-05
ENSBTAG00000004617	91.4422	-1.7030	0.4294	-3.9659	7.31E-05	4.50E-03
ENSBTAG00000000113	568.1471	-1.7081	0.3896	-4.3838	1.17E-05	1.04E-03
ENSBTAG00000016656	610.4891	-1.7117	0.3590	-4.7678	1.86E-06	2.22E-04
ENSBTAG00000015710	269.7640	-1.7149	0.4472	-3.8345	1.26E-04	6.66E-03
ENSBTAG00000032764	222.0430	-1.7253	0.4510	-3.8253	1.31E-04	6.79E-03
ENSBTAG00000007071	659.7378	-1.7344	0.3659	-4.7407	2.13E-06	2.47E-04
ENSBTAG00000047591	224.3186	-1.7432	0.4614	-3.7779	1.58E-04	7.79E-03

ENSBTAG00000006324	857.0865	-1.7596	0.4120	-4.2707	1.95E-05	1.61E-03
ENSBTAG00000008471	170.0698	-1.7603	0.4655	-3.7813	1.56E-04	7.74E-03
ENSBTAG00000004587	378.1371	-1.7638	0.3608	-4.8884	1.02E-06	1.25E-04
ENSBTAG00000001511	1359.1914	-1.7641	0.3578	-4.9309	8.18E-07	1.03E-04
ENSBTAG00000000706	2562.4333	-1.7792	0.3835	-4.6392	3.50E-06	3.78E-04
ENSBTAG00000010365	188.2796	-1.8059	0.4641	-3.8911	9.98E-05	5.72E-03
ENSBTAG00000015127	1669.8960	-1.8208	0.4844	-3.7592	1.70E-04	8.25E-03
ENSBTAG00000006194	105.5047	-1.8318	0.4773	-3.8378	1.24E-04	6.66E-03
ENSBTAG00000021595	220.1362	-1.8417	0.4788	-3.8469	1.20E-04	6.56E-03
ENSBTAG00000010785	213.1850	-1.8573	0.2704	-6.8690	6.47E-12	2.75E-09
ENSBTAG00000021435	1119.2098	-1.8584	0.3501	-5.3083	1.11E-07	1.87E-05
ENSBTAG00000001683	1158.1737	-1.8678	0.4103	-4.5517	5.32E-06	5.38E-04
ENSBTAG00000020764	504.9261	-1.8689	0.4401	-4.2466	2.17E-05	1.73E-03
ENSBTAG00000023144	922.1884	-1.8727	0.4623	-4.0511	5.10E-05	3.34E-03
ENSBTAG00000020772	182.3079	-1.8809	0.4968	-3.7862	1.53E-04	7.64E-03
ENSBTAG00000011421	178.8995	-1.8884	0.4720	-4.0010	6.31E-05	3.97E-03
ENSBTAG00000045948	96.0723	-1.9003	0.5080	-3.7411	1.83E-04	8.81E-03
ENSBTAG00000005477	2247.4331	-1.9062	0.4787	-3.9823	6.83E-05	4.26E-03
ENSBTAG00000008631	946.7466	-1.9092	0.5062	-3.7719	1.62E-04	7.95E-03
ENSBTAG00000008596	149.9558	-1.9092	0.4221	-4.5235	6.08E-06	5.93E-04
ENSBTAG00000003490	103.7564	-1.9178	0.4854	-3.9507	7.79E-05	4.67E-03
ENSBTAG00000048122	313.7418	-1.9236	0.5061	-3.8008	1.44E-04	7.34E-03
ENSBTAG00000010447	357.3259	-1.9267	0.4948	-3.8941	9.86E-05	5.69E-03
ENSBTAG00000002082	2168.9304	-1.9324	0.3942	-4.9023	9.47E-07	1.18E-04
ENSBTAG00000045742	51.8540	-1.9324	0.5199	-3.7172	2.01E-04	9.46E-03
ENSBTAG00000034529	68.0589	-1.9377	0.4499	-4.3072	1.65E-05	1.41E-03
ENSBTAG00000006608	62.9427	-1.9476	0.5082	-3.8320	1.27E-04	6.66E-03
ENSBTAG00000006552	97.4345	-1.9519	0.4670	-4.1793	2.92E-05	2.18E-03
ENSBTAG00000013730	77.2988	-1.9628	0.5296	-3.7059	2.11E-04	9.79E-03
ENSBTAG00000016551	149.9808	-1.9715	0.4896	-4.0272	5.65E-05	3.62E-03
ENSBTAG00000024470	46.5805	-1.9765	0.4999	-3.9535	7.70E-05	4.64E-03
ENSBTAG00000027655	112.3292	-1.9801	0.4336	-4.5665	4.96E-06	5.12E-04
ENSBTAG00000016315	1484.6976	-1.9919	0.4586	-4.3432	1.40E-05	1.22E-03
ENSBTAG00000031750	252.0250	-1.9954	0.4888	-4.0822	4.46E-05	3.04E-03
ENSBTAG00000020294	412.0946	-1.9988	0.5386	-3.7112	2.06E-04	9.62E-03
ENSBTAG00000024715	108.0569	-2.0008	0.5210	-3.8404	1.23E-04	6.66E-03
ENSBTAG00000001198	93.7670	-2.0033	0.5093	-3.9332	8.38E-05	4.97E-03
ENSBTAG00000004989	135.9989	-2.0048	0.4819	-4.1602	3.18E-05	2.33E-03
ENSBTAG00000034366	599.7981	-2.0078	0.5201	-3.8602	1.13E-04	6.26E-03
ENSBTAG00000020554	130.4078	-2.0092	0.5293	-3.7956	1.47E-04	7.44E-03
ENSBTAG00000009554	241.3348	-2.0101	0.5242	-3.8342	1.26E-04	6.66E-03
ENSBTAG00000020283	49.0945	-2.0180	0.5165	-3.9070	9.35E-05	5.44E-03
ENSBTAG00000024928	544.8249	-2.0197	0.4658	-4.3355	1.45E-05	1.25E-03

ENSBTAG00000015228	3507.7063	-2.0255	0.4461	-4.5408	5.60E-06	5.58E-04
ENSBTAG00000023338	10783.5322	-2.0445	0.2994	-6.8293	8.54E-12	3.52E-09
ENSBTAG00000010645	1824.9242	-2.0448	0.4488	-4.5561	5.21E-06	5.30E-04
ENSBTAG00000005978	109.3698	-2.0501	0.5254	-3.9023	9.53E-05	5.53E-03
ENSBTAG00000031701	98.2792	-2.0565	0.5282	-3.8932	9.89E-05	5.69E-03
ENSBTAG00000009137	90.7610	-2.0614	0.5363	-3.8436	1.21E-04	6.60E-03
ENSBTAG00000000641	37.3270	-2.0690	0.5230	-3.9562	7.61E-05	4.63E-03
ENSBTAG00000006039	627.8342	-2.0721	0.5341	-3.8797	1.05E-04	5.92E-03
ENSBTAG00000046158	849.2807	-2.0788	0.4992	-4.1645	3.12E-05	2.32E-03
ENSBTAG00000047448	168.2597	-2.0830	0.5533	-3.7647	1.67E-04	8.13E-03
ENSBTAG00000037510	42.9497	-2.0853	0.5333	-3.9104	9.21E-05	5.39E-03
ENSBTAG00000011545	24.8786	-2.0864	0.5607	-3.7210	1.98E-04	9.38E-03
ENSBTAG00000021672	87.2685	-2.0875	0.5512	-3.7869	1.53E-04	7.64E-03
ENSBTAG00000009717	247.6319	-2.0902	0.5493	-3.8053	1.42E-04	7.28E-03
ENSBTAG00000003014	38.6226	-2.0927	0.5177	-4.0423	5.29E-05	3.44E-03
ENSBTAG00000012700	126.9652	-2.1007	0.4642	-4.5257	6.02E-06	5.91E-04
ENSBTAG00000010888	346.8770	-2.1065	0.5168	-4.0761	4.58E-05	3.09E-03
ENSBTAG00000005628	245.6256	-2.1067	0.5119	-4.1156	3.86E-05	2.70E-03
ENSBTAG00000008636	1383.1741	-2.1068	0.4143	-5.0858	3.66E-07	5.14E-05
ENSBTAG00000015562	87.6411	-2.1069	0.5498	-3.8324	1.27E-04	6.66E-03
ENSBTAG00000000715	104.6538	-2.1079	0.5040	-4.1820	2.89E-05	2.17E-03
ENSBTAG00000000306	91.3153	-2.1134	0.5363	-3.9406	8.13E-05	4.86E-03
ENSBTAG00000005120	20.6659	-2.1208	0.5711	-3.7135	2.04E-04	9.57E-03
ENSBTAG00000036154	13.5483	-2.1253	0.5600	-3.7951	1.48E-04	7.44E-03
ENSBTAG00000018015	28.1663	-2.1320	0.5485	-3.8869	1.02E-04	5.80E-03
ENSBTAG00000047676	47.4178	-2.1329	0.5382	-3.9629	7.40E-05	4.54E-03
ENSBTAG00000037527	157.1798	-2.1343	0.5019	-4.2524	2.12E-05	1.71E-03
ENSBTAG00000021647	33.8871	-2.1405	0.5680	-3.7681	1.65E-04	8.05E-03
ENSBTAG00000006998	123.6625	-2.1500	0.5622	-3.8243	1.31E-04	6.79E-03
ENSBTAG00000012082	164.7846	-2.1502	0.5247	-4.0981	4.17E-05	2.88E-03
ENSBTAG00000015707	70.5117	-2.1871	0.5284	-4.1391	3.49E-05	2.52E-03
ENSBTAG00000026963	84.2828	-2.2067	0.4899	-4.5042	6.66E-06	6.36E-04
ENSBTAG00000016771	5885.8844	-2.2153	0.3047	-7.2697	3.60E-13	2.11E-10
ENSBTAG00000025405	51.5758	-2.2157	0.4878	-4.5427	5.55E-06	5.57E-04
ENSBTAG00000039160	145.5827	-2.2170	0.5287	-4.1937	2.74E-05	2.11E-03
ENSBTAG00000007584	207.9883	-2.2228	0.5308	-4.1877	2.82E-05	2.13E-03
ENSBTAG00000011207	128.1104	-2.2235	0.5457	-4.0746	4.61E-05	3.10E-03
ENSBTAG00000000431	112.0932	-2.2246	0.3787	-5.8737	4.26E-09	1.07E-06
ENSBTAG00000014090	280.2712	-2.2248	0.4423	-5.0295	4.92E-07	6.51E-05
ENSBTAG00000027204	175.7894	-2.2263	0.4879	-4.5630	5.04E-06	5.17E-04
ENSBTAG00000018011	83.8480	-2.2300	0.5251	-4.2466	2.17E-05	1.73E-03
ENSBTAG00000011043	534.5203	-2.2382	0.5230	-4.2796	1.87E-05	1.57E-03
ENSBTAG00000006707	560.2696	-2.2396	0.5022	-4.4594	8.22E-06	7.74E-04

ENSBTAG00000020872	200.3854	-2.2474	0.5445	-4.1273	3.67E-05	2.59E-03
ENSBTAG00000001585	325.4032	-2.2534	0.4969	-4.5351	5.76E-06	5.69E-04
ENSBTAG00000018366	79.8980	-2.2535	0.5288	-4.2619	2.03E-05	1.65E-03
ENSBTAG00000020034	696.0466	-2.2602	0.4660	-4.8502	1.23E-06	1.51E-04
ENSBTAG00000005390	184.2788	-2.2625	0.5365	-4.2172	2.47E-05	1.93E-03
ENSBTAG00000018869	119.0481	-2.2659	0.5531	-4.0970	4.19E-05	2.88E-03
ENSBTAG00000014560	36.6389	-2.2691	0.5336	-4.2526	2.11E-05	1.71E-03
ENSBTAG00000006505	1446.9165	-2.2843	0.5340	-4.2781	1.88E-05	1.57E-03
ENSBTAG00000009493	354.7421	-2.2884	0.4295	-5.3280	9.93E-08	1.72E-05
ENSBTAG00000019272	53.9647	-2.2961	0.5633	-4.0760	4.58E-05	3.09E-03
ENSBTAG00000014237	311.6978	-2.2980	0.4840	-4.7476	2.06E-06	2.43E-04
ENSBTAG00000011578	939.0683	-2.3025	0.4463	-5.1587	2.49E-07	3.64E-05
ENSBTAG00000003692	1007.7128	-2.3174	0.3291	-7.0417	1.90E-12	9.19E-10
ENSBTAG00000013496	149.9198	-2.3224	0.5479	-4.2388	2.25E-05	1.77E-03
ENSBTAG00000005892	129.5017	-2.3230	0.5507	-4.2184	2.46E-05	1.93E-03
ENSBTAG00000013689	32.7745	-2.3288	0.5703	-4.0833	4.44E-05	3.04E-03
ENSBTAG00000021709	322.1426	-2.3387	0.5027	-4.6522	3.28E-06	3.66E-04
ENSBTAG00000006984	359.0657	-2.3440	0.5275	-4.4436	8.85E-06	8.28E-04
ENSBTAG00000016770	43.1105	-2.3569	0.5666	-4.1600	3.18E-05	2.33E-03
ENSBTAG00000020676	22.7972	-2.3797	0.5725	-4.1568	3.23E-05	2.35E-03
ENSBTAG00000006859	20.3365	-2.3814	0.5725	-4.1595	3.19E-05	2.33E-03
ENSBTAG00000005018	93.2783	-2.3839	0.5412	-4.4053	1.06E-05	9.63E-04
ENSBTAG00000008497	45.0002	-2.3943	0.5693	-4.2059	2.60E-05	2.02E-03
ENSBTAG00000014612	169.2886	-2.4008	0.5187	-4.6282	3.69E-06	3.92E-04
ENSBTAG00000001824	163.9868	-2.4046	0.5111	-4.7044	2.55E-06	2.88E-04
ENSBTAG00000007626	525.4319	-2.4150	0.5029	-4.8021	1.57E-06	1.90E-04
ENSBTAG00000024272	78.2784	-2.4205	0.5678	-4.2628	2.02E-05	1.65E-03
ENSBTAG00000022622	168.3787	-2.4255	0.4192	-5.7866	7.18E-09	1.71E-06
ENSBTAG00000013368	50.9387	-2.4268	0.5659	-4.2887	1.80E-05	1.52E-03
ENSBTAG00000004043	519.6553	-2.4321	0.5282	-4.6040	4.14E-06	4.34E-04
ENSBTAG00000015978	1082.3857	-2.4425	0.4473	-5.4606	4.75E-08	9.13E-06
ENSBTAG00000008959	104.5673	-2.4446	0.4633	-5.2767	1.32E-07	2.17E-05
ENSBTAG00000020884	688.3009	-2.4698	0.4701	-5.2534	1.49E-07	2.41E-05
ENSBTAG00000019293	28.0422	-2.4811	0.5725	-4.3335	1.47E-05	1.26E-03
ENSBTAG00000005043	2834.4199	-2.4844	0.4669	-5.3212	1.03E-07	1.76E-05
ENSBTAG00000012894	266.7741	-2.4853	0.4935	-5.0365	4.74E-07	6.35E-05
ENSBTAG00000023283	99.6254	-2.4885	0.5386	-4.6201	3.84E-06	4.05E-04
ENSBTAG00000008573	2796.9823	-2.4936	0.3676	-6.7829	1.18E-11	4.72E-09
ENSBTAG00000006466	600.2903	-2.4969	0.4677	-5.3389	9.35E-08	1.66E-05
ENSBTAG00000008142	318.9061	-2.5112	0.4251	-5.9077	3.47E-09	9.02E-07
ENSBTAG00000039731	225.3526	-2.5131	0.5043	-4.9834	6.25E-07	8.12E-05
ENSBTAG00000018280	52.5312	-2.5137	0.5580	-4.5051	6.63E-06	6.36E-04
ENSBTAG00000007079	1221.6559	-2.5196	0.4781	-5.2702	1.36E-07	2.23E-05

ENSBTAG00000006780	65.5421	-2.5213	0.5426	-4.6468	3.37E-06	3.68E-04
ENSBTAG00000034918	262.2156	-2.5366	0.5384	-4.7112	2.46E-06	2.81E-04
ENSBTAG00000014762	147.4314	-2.5410	0.5438	-4.6725	2.98E-06	3.34E-04
ENSBTAG00000009614	89.3408	-2.5438	0.4975	-5.1136	3.16E-07	4.48E-05
ENSBTAG00000002615	169.9228	-2.5454	0.4047	-6.2897	3.18E-10	1.02E-07
ENSBTAG00000016683	3632.6408	-2.5624	0.4215	-6.0791	1.21E-09	3.39E-07
ENSBTAG00000011011	133.8720	-2.5962	0.4748	-5.4680	4.55E-08	8.95E-06
ENSBTAG00000047379	90.9785	-2.6217	0.5529	-4.7420	2.12E-06	2.47E-04
ENSBTAG00000016357	443.7551	-2.6234	0.5026	-5.2196	1.79E-07	2.83E-05
ENSBTAG00000011784	618.6797	-2.6290	0.5054	-5.2021	1.97E-07	3.04E-05
ENSBTAG00000011515	281.5105	-2.6415	0.5058	-5.2224	1.77E-07	2.82E-05
ENSBTAG00000020674	199.0760	-2.6707	0.4477	-5.9660	2.43E-09	6.44E-07
ENSBTAG00000048155	697.2993	-2.6745	0.5135	-5.2082	1.91E-07	2.97E-05
ENSBTAG00000046409	51.9913	-2.6821	0.5680	-4.7217	2.34E-06	2.69E-04
ENSBTAG00000009214	269.7820	-2.6846	0.4769	-5.6291	1.81E-08	4.04E-06
ENSBTAG00000001009	331.1292	-2.6969	0.4801	-5.6171	1.94E-08	4.26E-06
ENSBTAG00000018984	142.8545	-2.6979	0.5346	-5.0462	4.51E-07	6.14E-05
ENSBTAG00000017722	105.4262	-2.7220	0.5515	-4.9359	7.98E-07	1.02E-04
ENSBTAG00000010069	5008.2430	-2.7297	0.5720	-4.7726	1.82E-06	2.18E-04
ENSBTAG00000005413	328.0298	-2.7482	0.5027	-5.4666	4.59E-08	8.95E-06
ENSBTAG00000016163	58.0262	-2.7483	0.5348	-5.1392	2.76E-07	3.99E-05
ENSBTAG00000014127	436.6241	-2.7566	0.5202	-5.2989	1.16E-07	1.95E-05
ENSBTAG00000000095	114.5427	-2.7905	0.5020	-5.5592	2.71E-08	5.68E-06
ENSBTAG00000012623	166.7796	-2.7920	0.4577	-6.1002	1.06E-09	3.03E-07
ENSBTAG00000014898	77.2625	-2.7980	0.5182	-5.3992	6.70E-08	1.23E-05
ENSBTAG00000001492	460.6527	-2.8119	0.5047	-5.5709	2.53E-08	5.39E-06
ENSBTAG00000001154	534.4186	-2.8312	0.4605	-6.1480	7.84E-10	2.34E-07
ENSBTAG00000007881	549.2791	-2.8319	0.4433	-6.3884	1.68E-10	6.04E-08
ENSBTAG00000007883	439.7353	-2.8784	0.3985	-7.2223	5.11E-13	2.76E-10
ENSBTAG00000033565	210.8597	-2.8941	0.4624	-6.2585	3.89E-10	1.21E-07
ENSBTAG00000013201	73.1055	-2.9171	0.5634	-5.1779	2.24E-07	3.42E-05
ENSBTAG00000001822	311.2196	-2.9674	0.4643	-6.3908	1.65E-10	6.04E-08
ENSBTAG00000039046	260.8241	-3.0122	0.5574	-5.4042	6.51E-08	1.22E-05
ENSBTAG00000014439	49.8320	-3.0708	0.5709	-5.3786	7.51E-08	1.35E-05
ENSBTAG00000031397	342.1739	-3.0742	0.5584	-5.5049	3.69E-08	7.41E-06
ENSBTAG00000006343	80.5291	-3.1016	0.5634	-5.5050	3.69E-08	7.41E-06
ENSBTAG00000014113	70.2286	-3.1230	0.5576	-5.6012	2.13E-08	4.60E-06
ENSBTAG00000002623	1187.2865	-3.1295	0.5109	-6.1259	9.02E-10	2.64E-07
ENSBTAG00000016593	430.1438	-3.1353	0.4497	-6.9717	3.13E-12	1.42E-09
ENSBTAG00000023179	462.5279	-3.1451	0.4435	-7.0917	1.32E-12	6.64E-10
ENSBTAG00000008223	108.2302	-3.1498	0.5205	-6.0513	1.44E-09	3.88E-07
ENSBTAG00000000436	506.5186	-3.2032	0.4455	-7.1896	6.50E-13	3.38E-10
ENSBTAG00000008792	120.3418	-3.2077	0.4586	-6.9941	2.67E-12	1.25E-09

ENSBTAG00000002215	2951.8734	-3.2305	0.5108	-6.3245	2.54E-10	8.70E-08
ENSBTAG00000008441	670.2671	-3.2374	0.3119	-10.3779	3.13E-25	7.31E-22
ENSBTAG00000037558	952.4427	-3.2714	0.5387	-6.0725	1.26E-09	3.47E-07
ENSBTAG00000006566	72.2048	-3.2920	0.5585	-5.8942	3.77E-09	9.61E-07
ENSBTAG00000006136	158.5240	-3.3196	0.5032	-6.5969	4.20E-11	1.59E-08
ENSBTAG00000012228	159.9890	-3.3217	0.4927	-6.7421	1.56E-11	6.09E-09
ENSBTAG00000018223	98.0262	-3.3402	0.4828	-6.9189	4.55E-12	2.00E-09
ENSBTAG00000014368	144.0335	-3.3724	0.5329	-6.3283	2.48E-10	8.70E-08
ENSBTAG00000016424	124.4214	-3.3761	0.5363	-6.2952	3.07E-10	1.00E-07
ENSBTAG00000011982	151.4779	-3.4422	0.4759	-7.2327	4.74E-13	2.66E-10
ENSBTAG00000010349	604.2005	-3.5274	0.3899	-9.0472	1.47E-19	1.87E-16
ENSBTAG00000025250	481.3402	-3.5511	0.4400	-8.0702	7.02E-16	5.19E-13
ENSBTAG00000035995	140.0171	-3.5717	0.4659	-7.6667	1.76E-14	1.13E-11
ENSBTAG00000009658	1176.7344	-3.8283	0.3917	-9.7744	1.45E-22	2.91E-19
ENSBTAG00000002340	131.8174	-3.9748	0.5228	-7.6029	2.89E-14	1.77E-11
ENSBTAG00000038042	366.6777	-4.0464	0.4974	-8.1355	4.10E-16	3.20E-13
ENSBTAG00000009768	368.8166	-4.1086	0.3826	-10.7401	6.60E-27	1.85E-23
ENSBTAG00000023026	239.8992	-4.1179	0.4758	-8.6539	4.98E-18	4.99E-15
ENSBTAG00000015865	418.9198	-4.1300	0.5205	-7.9342	2.12E-15	1.49E-12
ENSBTAG00000013451	261.0602	-4.1319	0.4522	-9.1379	6.37E-20	9.93E-17
ENSBTAG00000013334	1230.1896	-4.1362	0.4811	-8.5967	8.20E-18	7.68E-15
ENSBTAG00000001273	286.8242	-4.1478	0.4768	-8.6988	3.35E-18	3.62E-15
ENSBTAG00000019665	11630.9040	-4.1733	0.5034	-8.2897	1.14E-16	9.38E-14
ENSBTAG00000019123	2240.4147	-4.2225	0.5071	-8.3263	8.34E-17	7.32E-14
ENSBTAG00000000283	409.5562	-4.3874	0.3836	-11.4366	2.74E-30	1.28E-26
ENSBTAG00000025471	208.8173	-4.5476	0.4943	-9.1994	3.60E-20	6.32E-17
ENSBTAG00000019716	1971.8177	-4.5815	0.5028	-9.1122	8.07E-20	1.13E-16
ENSBTAG00000015592	688.0430	-4.5932	0.5146	-8.9263	4.41E-19	5.15E-16
ENSBTAG00000047904	1210.2309	-4.8803	0.4489	-10.8709	1.59E-27	5.57E-24
ENSBTAG00000001321	6966.0164	-5.3873	0.4111	-13.1040	3.12E-39	4.39E-35
ENSBTAG00000014555	140.1466	-5.5119	0.4596	-11.9919	3.92E-33	2.75E-29

Supplementary Table 4: List of 210 DEGs commonly regulated between two pairwise comparisons being Vehicle + GnRH vs Vehicle and PD0325901 +GnRH vs Vehicle + GnRH

	Vehicle + GnRH vs Vehicle						PD0325901 + GnRH vs Vehicle + GnRH					
ENSEMBL **ID**	baseMean	log2Fold Change	lfcSE	stat	pvalue	padj	baseMean	log2Fold Change	lfcSE	stat	pvalue	padj
ENSBTAG00000048122	327.5941	5.4845	0.7140	7.6809	1.58E-14	8.99E-13	313.7418	-1.9236	0.5061	-3.8008	1.44E-04	7.34E-03
ENSBTAG00000006505	1588.9178	8.2961	0.8419	9.8543	6.57E-23	1.17E-20	1446.9165	-2.2843	0.5340	-4.2781	1.88E-05	1.57E-03
ENSBTAG00000000715	111.0114	6.2339	0.8437	7.3891	1.48E-13	7.49E-12	104.6538	-2.1079	0.5040	-4.1820	2.89E-05	2.17E-03
ENSBTAG00000021709	355.1579	4.3869	0.8655	5.0687	4.01E-07	6.78E-06	322.1426	-2.3387	0.5027	-4.6522	3.28E-06	3.66E-04
ENSBTAG00000006039	690.8921	4.1795	0.8265	5.0568	4.26E-07	7.16E-06	627.8342	-2.0721	0.5341	-3.8797	1.05E-04	5.92E-03
ENSBTAG00000004587	376.6274	3.8306	0.5913	6.4782	9.28E-11	3.11E-09	378.1371	-1.7638	0.3608	-4.8884	1.02E-06	1.25E-04
ENSBTAG00000018015	31.1991	4.6974	1.0390	4.5211	6.15E-06	7.80E-05	28.1663	-2.1320	0.5485	-3.8869	1.02E-04	5.80E-03
ENSBTAG00000008441	807.6836	2.7425	0.4776	5.7421	9.35E-09	2.17E-07	670.2671	-3.2374	0.3119	-10.3779	3.13E-25	7.31E-22
ENSBTAG00000006566	85.5741	6.3878	1.0685	5.9782	2.26E-09	5.86E-08	72.2048	-3.2920	0.5585	-5.8942	3.77E-09	9.61E-07
ENSBTAG00000034918	294.4042	8.0883	0.9822	8.2350	1.80E-16	1.33E-14	262.2156	-2.5366	0.5384	-4.7112	2.46E-06	2.81E-04
ENSBTAG00000027204	187.9170	5.7430	0.8482	6.7706	1.28E-11	4.98E-10	175.7894	-2.2263	0.4879	-4.5630	5.04E-06	5.17E-04
ENSBTAG00000011578	1001.1047	5.1227	0.7674	6.6753	2.47E-11	9.12E-10	939.0683	-2.3025	0.4463	-5.1587	2.49E-07	3.64E-05
ENSBTAG00000018223	112.6090	6.7027	0.9003	7.4450	9.69E-14	5.02E-12	98.0262	-3.3402	0.4828	-6.9189	4.55E-12	2.00E-09
ENSBTAG00000010447	371.9605	5.2630	0.6808	7.7309	1.07E-14	6.20E-13	357.3259	-1.9267	0.4948	-3.8941	9.86E-05	5.69E-03
ENSBTAG00000019272	61.6389	5.2167	1.0583	4.9293	8.25E-07	1.29E-05	53.9647	-2.2961	0.5633	-4.0760	4.58E-05	3.09E-03
ENSBTAG00000037527	170.2118	4.1358	0.8534	4.8462	1.26E-06	1.89E-05	157.1798	-2.1343	0.5019	-4.2524	2.12E-05	1.71E-03
ENSBTAG00000001822	381.0149	2.6346	0.6275	4.1985	2.69E-05	2.91E-04	311.2196	-2.9674	0.4643	-6.3908	1.65E-10	6.04E-08
ENSBTAG00000010645	1929.3493	3.8248	0.7917	4.8314	1.36E-06	2.03E-05	1824.9242	-2.0448	0.4488	-4.5561	5.21E-06	5.30E-04
ENSBTAG00000016770	49.7905	5.2327	1.0376	5.0431	4.58E-07	7.63E-06	43.1105	-2.3569	0.5666	-4.1600	3.18E-05	2.33E-03
ENSBTAG00000001321	8261.7540	12.0968	0.7821	15.4678	5.73E-54	7.72E-51	6966.0164	-5.3873	0.4111	-13.1040	3.12E-39	4.39E-35
ENSBTAG00000017722	121.9211	5.9106	0.8493	6.9595	3.41E-12	1.43E-10	105.4262	-2.7220	0.5515	-4.9359	7.98E-07	1.02E-04
ENSBTAG00000015978	1214.7347	3.4950	0.6008	5.8169	5.99E-09	1.43E-07	1082.3857	-2.4425	0.4473	-5.4606	4.75E-08	9.13E-06
ENSBTAG00000016683	4101.8910	3.4281	0.6030	5.6854	1.31E-08	2.92E-07	3632.6408	-2.5624	0.4215	-6.0791	1.21E-09	3.39E-07
ENSBTAG00000006466	654.3450	5.7105	0.7227	7.9015	2.76E-15	1.80E-13	600.2903	-2.4969	0.4677	-5.3389	9.35E-08	1.66E-05
ENSBTAG00000031231	1742.5838	2.5274	0.5295	4.7737	1.81E-06	2.60E-05	1703.1230	-1.4974	0.4019	-3.7256	1.95E-04	9.27E-03
ENSBTAG00000007052	143.5162	7.7433	0.9536	8.1200	4.66E-16	3.29E-14	157.3967	-1.5454	0.2992	-5.1648	2.41E-07	3.56E-05
ENSBTAG00000005978	123.8618	2.9747	0.8356	3.5601	3.71E-04	2.82E-03	109.3698	-2.0501	0.5254	-3.9023	9.53E-05	5.53E-03
ENSBTAG00000048155	783.7776	4.0557	1.1877	3.4148	6.38E-04	4.50E-03	697.2993	-2.6745	0.5135	-5.2082	1.91E-07	2.97E-05
ENSBTAG00000016315	1625.7506	2.9772	0.6102	4.8787	1.07E-06	1.65E-05	1484.6976	-1.9919	0.4586	-4.3432	1.40E-05	1.22E-03
ENSBTAG00000000095	129.1531	5.5902	0.8921	6.2661	3.70E-10	1.12E-08	114.5427	-2.7905	0.5020	-5.5592	2.71E-08	5.68E-06
ENSBTAG00000017745	2731.2206	1.8411	0.5019	3.6681	2.44E-04	1.98E-03	2572.0794	-1.4590	0.3302	-4.4189	9.92E-06	9.16E-04
ENSBTAG00000016217	1221.4838	1.8640	0.4867	3.8297	1.28E-04	1.14E-03	1124.6114	-1.5456	0.3691	-4.1879	2.82E-05	2.13E-03
ENSBTAG00000008142	353.7349	3.8428	0.6144	6.2542	4.00E-10	1.19E-08	318.9061	-2.5112	0.4251	-5.9077	3.47E-09	9.02E-07
ENSBTAG00000006130	316.0747	6.2784	0.6496	9.6652	4.24E-22	6.72E-20	334.3320	-1.6901	0.3272	-5.1651	2.40E-07	3.56E-05
ENSBTAG00000008471	171.4890	4.1733	0.9106	4.5831	4.58E-06	6.00E-05	170.0698	-1.7603	0.4655	-3.7813	1.56E-04	7.74E-03
ENSBTAG00000000266	2929.2015	2.2579	0.3066	7.3639	1.79E-13	8.92E-12	3123.6644	-1.1006	0.2185	-5.0363	4.75E-07	6.35E-05
ENSBTAG00000012700	136.2740	3.8563	0.6767	5.6984	1.21E-08	2.74E-07	126.9652	-2.1007	0.4642	-4.5257	6.02E-06	5.91E-04
ENSBTAG00000008401	1158.5253	1.7883	0.5377	3.3258	8.82E-04	5.84E-03	1050.9935	-1.6421	0.3285	-4.9984	5.78E-07	7.58E-05

ENSBTAG00000023179	523.8677	5.9045	0.6573	8.9828	2.64E-19	2.82E-17	462.5279	-3.1451	0.4435	-7.0917	1.32E-12	6.64E-10
ENSBTAG00000009614	100.0067	4.5149	0.8063	5.5997	2.15E-08	4.61E-07	89.3408	-2.5438	0.4975	-5.1136	3.16E-07	4.48E-05
ENSBTAG00000014898	90.3875	3.7480	0.8319	4.5054	6.63E-06	8.36E-05	77.2625	-2.7980	0.5182	-5.3992	6.70E-08	1.23E-05
ENSBTAG00000033565	238.4475	4.8702	0.7168	6.7948	1.08E-11	4.25E-10	210.8597	-2.8941	0.4624	-6.2585	3.89E-10	1.21E-07
ENSBTAG00000025471	247.9527	7.4121	0.9136	8.1130	4.94E-16	3.45E-14	208.8173	-4.5476	0.4943	-9.1994	3.60E-20	6.32E-17
ENSBTAG00000039046	305.4257	7.8987	1.0044	7.8639	3.72E-15	2.39E-13	260.8241	-3.0122	0.5574	-5.4042	6.51E-08	1.22E-05
ENSBTAG00000005477	2360.4130	4.0406	0.6275	6.4392	1.20E-10	3.96E-09	2247.4331	-1.9062	0.4787	-3.9823	6.83E-05	4.26E-03
ENSBTAG00000013334	1444.2931	9.3142	0.8107	11.4891	1.50E-30	4.12E-28	1230.1896	-4.1362	0.4811	-8.5967	8.20E-18	7.68E-15
ENSBTAG00000015212	986.8776	1.3979	0.3732	3.7462	1.80E-04	1.53E-03	888.2039	-1.4380	0.2697	-5.3320	9.72E-08	1.70E-05
ENSBTAG00000011043	587.1969	5.2279	0.6389	8.1826	2.78E-16	2.00E-14	534.5203	-2.2382	0.5230	-4.2796	1.87E-05	1.57E-03
ENSBTAG00000020884	780.5113	3.3763	0.7402	4.5613	5.08E-06	6.57E-05	688.3009	-2.4698	0.4701	-5.2534	1.49E-07	2.41E-05
ENSBTAG00000000641	40.0206	5.2653	0.9681	5.4390	5.36E-08	1.07E-06	37.3270	-2.0690	0.5230	-3.9562	7.61E-05	4.63E-03
ENSBTAG00000013689	38.3357	4.6895	1.0766	4.3558	1.33E-05	1.57E-04	32.7745	-2.3288	0.5703	-4.0833	4.44E-05	3.04E-03
ENSBTAG00000016771	5946.4817	7.9561	0.5101	15.5962	7.72E-55	1.30E-51	5885.8844	-2.2153	0.3047	-7.2697	3.60E-13	2.11E-10
ENSBTAG00000002215	3554.3251	3.3218	0.8435	3.9379	8.22E-05	7.70E-04	2951.8734	-3.2305	0.5108	-6.3245	2.54E-10	8.70E-08
ENSBTAG00000036154	15.3533	3.9670	1.0715	3.7022	2.14E-04	1.77E-03	13.5483	-2.1253	0.5600	-3.7951	1.48E-04	7.44E-03
ENSBTAG00000013201	88.3757	4.0007	0.8574	4.6660	3.07E-06	4.19E-05	73.1055	-2.9171	0.5634	-5.1779	2.24E-07	3.42E-05
ENSBTAG00000047676	52.9694	3.7485	0.9473	3.9571	7.59E-05	7.18E-04	47.4178	-2.1329	0.5382	-3.9629	7.40E-05	4.54E-03
ENSBTAG00000018869	135.3945	4.4886	0.8393	5.3480	8.89E-08	1.74E-06	119.0481	-2.2659	0.5531	-4.0970	4.19E-05	2.88E-03
ENSBTAG00000020674	223.5519	4.0561	0.7117	5.6990	1.21E-08	2.74E-07	199.0760	-2.6707	0.4477	-5.9660	2.43E-09	6.44E-07
ENSBTAG00000008223	127.5209	3.8935	0.9997	3.8945	9.84E-05	9.06E-04	108.2302	-3.1498	0.5205	-6.0513	1.44E-09	3.88E-07
ENSBTAG00000002623	1362.8380	7.5297	0.7740	9.7279	2.29E-22	3.72E-20	1187.2865	-3.1295	0.5109	-6.1259	9.02E-10	2.64E-07
ENSBTAG00000022622	192.1988	2.9760	0.4114	7.2337	4.70E-13	2.25E-11	168.3787	-2.4255	0.4192	-5.7866	7.18E-09	1.71E-06
ENSBTAG00000037510	46.7588	4.6889	0.9902	4.7351	2.19E-06	3.08E-05	42.9497	-2.0853	0.5333	-3.9104	9.21E-05	5.39E-03
ENSBTAG00000010069	6423.0355	2.6736	0.8367	3.1953	1.40E-03	8.52E-03	5008.2430	-2.7297	0.5720	-4.7726	1.82E-06	2.18E-04
ENSBTAG00000012894	291.7767	6.9739	0.8535	8.1706	3.07E-16	2.20E-14	266.7741	-2.4853	0.4935	-5.0365	4.74E-07	6.35E-05
ENSBTAG00000016061	309.7472	5.0918	0.7749	6.5713	4.99E-11	1.77E-09	319.5733	-1.6452	0.4298	-3.8282	1.29E-04	6.74E-03
ENSBTAG00000018280	60.1686	6.2571	1.0568	5.9208	3.20E-09	8.09E-08	52.5312	-2.5137	0.5580	-4.5051	6.63E-06	6.36E-04
ENSBTAG00000012623	189.8271	4.0542	0.6190	6.5498	5.76E-11	1.99E-09	166.7796	-2.7920	0.4577	-6.1002	1.06E-09	3.03E-07
ENSBTAG00000001273	337.2373	7.1719	0.8459	8.4785	2.28E-17	1.90E-15	286.8242	-4.1478	0.4768	-8.6988	3.35E-18	3.62E-15
ENSBTAG00000004322	3346.2945	5.1600	0.5448	9.4716	2.76E-21	4.00E-19	3890.2670	-1.1885	0.2740	-4.3381	1.44E-05	1.25E-03
ENSBTAG00000021818	1523.3348	2.1835	0.3875	5.6344	1.76E-08	3.86E-07	1496.0128	-1.4054	0.3196	-4.3971	1.10E-05	9.94E-04
ENSBTAG00000020872	223.4640	5.1804	0.8212	6.3084	2.82E-10	8.82E-09	200.3854	-2.2474	0.5445	-4.1273	3.67E-05	2.59E-03
ENSBTAG00000000436	579.8521	5.0889	0.6291	8.0889	6.02E-16	4.16E-14	506.5186	-3.2032	0.4455	-7.1896	6.50E-13	3.38E-10
ENSBTAG00000015865	495.4450	8.3334	0.8907	9.3565	8.24E-21	1.13E-18	418.9198	-4.1300	0.5205	-7.9342	2.12E-15	1.49E-12
ENSBTAG00000038428	725.4953	3.0527	0.4116	7.4172	1.20E-13	6.13E-12	788.2573	-1.2147	0.3085	-3.9381	8.21E-05	4.89E-03
ENSBTAG00000010888	382.1166	3.8445	0.7601	5.0577	4.24E-07	7.14E-06	346.8770	-2.1065	0.5168	-4.0761	4.58E-05	3.09E-03
ENSBTAG00000007079	1342.4463	5.4228	0.6980	7.7696	7.88E-15	4.68E-13	1221.6559	-2.5196	0.4781	-5.2702	1.36E-07	2.23E-05
ENSBTAG00000006194	107.2551	4.9577	0.8790	5.6400	1.70E-08	3.75E-07	105.5047	-1.8318	0.4773	-3.8378	1.24E-04	6.66E-03
ENSBTAG00000015562	96.3725	6.0284	0.8559	7.0432	1.88E-12	8.20E-11	87.6411	-2.1069	0.5498	-3.8324	1.27E-04	6.66E-03
ENSBTAG00000037558	1112.0383	8.7569	0.9449	9.2675	1.91E-20	2.31E-18	952.4427	-3.2714	0.5387	-6.0725	1.26E-09	3.47E-07
ENSBTAG00000027655	127.7520	2.3075	0.4631	4.9826	6.27E-07	1.01E-05	112.3292	-1.9801	0.4336	-4.5665	4.96E-06	5.12E-04
ENSBTAG00000002340	155.9542	7.2697	1.0188	7.1353	9.66E-13	4.37E-11	131.8174	-3.9748	0.5228	-7.6029	2.89E-14	1.77E-11

ENSBTAG00000018984	163.8858	5.0499	0.8383	6.0240	1.70E-09	4.56E-08	142.8545	-2.6979	0.5346	-5.0462	4.51E-07	6.14E-05
ENSBTAG00000006343	95.3510	6.7426	1.0380	6.4956	8.27E-11	2.79E-09	80.5291	-3.1016	0.5634	-5.5050	3.69E-08	7.41E-06
ENSBTAG00000047904	1431.0922	10.5501	0.8595	12.2752	1.23E-34	4.37E-32	1210.2309	-4.8803	0.4489	-10.8709	1.59E-27	5.57E-24
ENSBTAG00000000706	2573.7218	3.8447	0.5065	7.5906	3.18E-14	1.74E-12	2562.4333	-1.7792	0.3835	-4.6392	3.50E-06	3.78E-04
ENSBTAG00000001576	95.9240	1.5823	0.4901	3.2284	1.24E-03	7.75E-03	85.1045	-1.5252	0.4013	-3.8011	1.44E-04	7.34E-03
ENSBTAG00000005120	23.7657	4.7800	1.1564	4.1337	3.57E-05	3.72E-04	20.6659	-2.1208	0.5711	-3.7135	2.04E-04	9.57E-03
ENSBTAG00000025250	553.0634	6.6539	0.7125	9.3386	9.76E-21	1.32E-18	481.3402	-3.5511	0.4400	-8.0702	7.02E-16	5.19E-13
ENSBTAG00000014612	190.5284	4.0545	0.8751	4.6330	3.60E-06	4.85E-05	169.2886	-2.4008	0.5187	-4.6282	3.69E-06	3.92E-04
ENSBTAG00000017457	416.0405	1.9160	0.5195	3.6880	2.26E-04	1.86E-03	381.4281	-1.6065	0.3680	-4.3655	1.27E-05	1.11E-03
ENSBTAG00000005043	3386.5329	2.4584	0.6333	3.8821	1.04E-04	9.46E-04	2834.4199	-2.4844	0.4669	-5.3212	1.03E-07	1.76E-05
ENSBTAG00000046409	61.2862	5.7135	0.9803	5.8283	5.60E-09	1.34E-07	51.9913	-2.6821	0.5680	-4.7217	2.34E-06	2.69E-04
ENSBTAG00000038042	431.1692	8.5962	0.9602	8.9524	3.48E-19	3.61E-17	366.6777	-4.0464	0.4974	-8.1355	4.10E-16	3.20E-13
ENSBTAG00000015707	76.3656	6.6953	1.0245	6.5355	6.34E-11	2.18E-09	70.5117	-2.1871	0.5284	-4.1391	3.49E-05	2.52E-03
ENSBTAG00000015228	3759.7133	3.3038	0.7716	4.2819	1.85E-05	2.09E-04	3507.7063	-2.0255	0.4461	-4.5408	5.60E-06	5.58E-04
ENSBTAG00000001154	606.2876	4.4443	0.6166	7.2080	5.68E-13	2.70E-11	534.4186	-2.8312	0.4605	-6.1480	7.84E-10	2.34E-07
ENSBTAG00000019293	34.5205	3.4063	1.0339	3.2947	9.85E-04	6.40E-03	28.0422	-2.4811	0.5725	-4.3335	1.47E-05	1.26E-03
ENSBTAG00000017763	2247.6335	2.7630	0.3848	7.1796	6.99E-13	3.22E-11	2518.4658	-1.0321	0.2561	-4.0302	5.57E-05	3.59E-03
ENSBTAG00000000283	481.0707	6.0077	0.5616	10.6982	1.04E-26	2.37E-24	409.5562	-4.3874	0.3836	-11.4366	2.74E-30	1.28E-26
ENSBTAG00000006780	74.1900	5.6700	1.0256	5.5284	3.23E-08	6.71E-07	65.5421	-2.5213	0.5426	-4.6468	3.37E-06	3.68E-04
ENSBTAG00000002624	506.4162	3.0441	0.6821	4.4629	8.09E-06	1.00E-04	495.6110	-1.6815	0.4094	-4.1071	4.01E-05	2.78E-03
ENSBTAG00000006984	421.5356	3.0164	0.6852	4.4024	1.07E-05	1.29E-04	359.0657	-2.3440	0.5275	-4.4436	8.85E-06	8.28E-04
ENSBTAG00000000431	115.6526	5.8634	0.6804	8.6180	6.81E-18	6.04E-16	112.0932	-2.2246	0.3787	-5.8737	4.26E-09	1.07E-06
ENSBTAG00000006707	608.9144	5.0087	0.6952	7.2044	5.83E-13	2.75E-11	560.2696	-2.2396	0.5022	-4.4594	8.22E-06	7.74E-04
ENSBTAG00000016593	496.6370	4.2463	0.7036	6.0353	1.59E-09	4.26E-08	430.1438	-3.1353	0.4497	-6.9717	3.13E-12	1.42E-09
ENSBTAG00000006608	67.4652	3.9000	0.8138	4.7924	1.65E-06	2.40E-05	62.9427	-1.9476	0.5082	-3.8320	1.27E-04	6.66E-03
ENSBTAG00000031397	404.1610	6.0031	0.8209	7.3130	2.61E-13	1.28E-11	342.1739	-3.0742	0.5584	-5.5049	3.69E-08	7.41E-06
ENSBTAG00000019123	2648.8416	8.7258	0.7078	12.3279	6.41E-35	2.34E-32	2240.4147	-4.2225	0.5071	-8.3263	8.34E-17	7.32E-14
ENSBTAG00000015710	266.4422	5.2398	0.6552	7.9975	1.27E-15	8.60E-14	269.7640	-1.7149	0.4472	-3.8345	1.26E-04	6.66E-03
ENSBTAG00000014368	168.5765	6.7992	0.9656	7.0413	1.91E-12	8.29E-11	144.0335	-3.3724	0.5329	-6.3283	2.48E-10	8.70E-08
ENSBTAG00000007881	607.7495	5.9727	0.8089	7.3840	1.54E-13	7.76E-12	549.2791	-2.8319	0.4433	-6.3884	1.68E-10	6.04E-08
ENSBTAG00000007626	579.6998	5.1881	0.7118	7.2892	3.12E-13	1.51E-11	525.4319	-2.4150	0.5029	-4.8021	1.57E-06	1.90E-04
ENSBTAG00000006859	24.1786	4.7045	1.1645	4.0400	5.35E-05	5.29E-04	20.3365	-2.3814	0.5725	-4.1595	3.19E-05	2.33E-03
ENSBTAG00000011421	191.7185	3.2160	0.6634	4.8478	1.25E-06	1.88E-05	178.8995	-1.8884	0.4720	-4.0010	6.31E-05	3.97E-03
ENSBTAG00000008091	81.9241	1.5478	0.4691	3.2995	9.69E-04	6.31E-03	72.2864	-1.5797	0.3720	-4.2459	2.18E-05	1.73E-03
ENSBTAG00000031750	266.8580	4.3600	0.6994	6.2338	4.55E-10	1.35E-08	252.0250	-1.9954	0.4888	-4.0822	4.46E-05	3.04E-03
ENSBTAG00000013451	305.3331	7.6876	0.7630	10.0754	7.09E-24	1.37E-21	261.0602	-4.1319	0.4522	-9.1379	6.37E-20	9.93E-17
ENSBTAG00000026963	89.6813	6.1490	0.9549	6.4394	1.20E-10	3.96E-09	84.2828	-2.2067	0.4899	-4.5042	6.66E-06	6.36E-04
ENSBTAG00000001585	364.9243	3.4498	0.7103	4.8571	1.19E-06	1.82E-05	325.4032	-2.2534	0.4969	-4.5351	5.76E-06	5.69E-04
ENSBTAG00000012082	183.0709	3.6516	0.9030	4.0440	5.26E-05	5.21E-04	164.7846	-2.1502	0.5247	-4.0981	4.17E-05	2.88E-03
ENSBTAG00000009717	273.1533	5.2859	0.8346	6.3333	2.40E-10	7.59E-09	247.6319	-2.0902	0.5493	-3.8053	1.42E-04	7.28E-03
ENSBTAG00000005892	145.1695	6.3447	1.0164	6.2422	4.31E-10	1.28E-08	129.5017	-2.3230	0.5507	-4.2184	2.46E-05	1.93E-03
ENSBTAG00000015127	1836.2790	2.6560	0.6347	4.1843	2.86E-05	3.07E-04	1669.8960	-1.8208	0.4844	-3.7592	1.70E-04	8.25E-03
ENSBTAG00000047448	185.6885	5.8566	0.8406	6.9670	3.24E-12	1.36E-10	168.2597	-2.0830	0.5533	-3.7647	1.67E-04	8.13E-03

ENSBTAG00000013730	81.9317	6.0302	0.9603	6.2794	3.40E-10	1.04E-08	77.2988	-1.9628	0.5296	-3.7059	2.11E-04	9.79E-03
ENSBTAG00000014127	498.5516	5.1029	0.8118	6.2859	3.26E-10	1.00E-08	436.6241	-2.7566	0.5202	-5.2989	1.16E-07	1.95E-05
ENSBTAG00000014090	301.1245	4.0289	0.7453	5.4058	6.45E-08	1.28E-06	280.2712	-2.2248	0.4423	-5.0295	4.92E-07	6.51E-05
ENSBTAG00000000306	99.4737	5.5107	0.8869	6.2134	5.18E-10	1.51E-08	91.3153	-2.1134	0.5363	-3.9406	8.13E-05	4.86E-03
ENSBTAG00000025405	54.9222	5.6817	0.9620	5.9059	3.51E-09	8.74E-08	51.5758	-2.2157	0.4878	-4.5427	5.55E-06	5.57E-04
ENSBTAG00000024648	2202.0628	2.5443	0.4461	5.7035	1.17E-08	2.67E-07	2184.1692	-1.5109	0.3252	-4.6461	3.38E-06	3.68E-04
ENSBTAG00000019665	13716.7991	10.2751	0.7304	14.0677	6.00E-45	4.49E-42	11630.9040	-4.1733	0.5034	-8.2897	1.14E-16	9.38E-14
ENSBTAG00000024470	48.8652	4.8490	0.9601	5.0504	4.41E-07	7.38E-06	46.5805	-1.9765	0.4999	-3.9535	7.70E-05	4.64E-03
ENSBTAG00000020034	739.0268	6.3440	0.6159	10.2995	7.08E-25	1.40E-22	696.0466	-2.2602	0.4660	-4.8502	1.23E-06	1.51E-04
ENSBTAG00000006552	99.8226	5.6552	0.8550	6.6144	3.73E-11	1.34E-09	97.4345	-1.9519	0.4670	-4.1793	2.92E-05	2.18E-03
ENSBTAG00000021672	96.6246	4.7017	0.9933	4.7334	2.21E-06	3.10E-05	87.2685	-2.0875	0.5512	-3.7869	1.53E-04	7.64E-03
ENSBTAG00000009493	383.4560	3.8837	0.7443	5.2177	1.81E-07	3.35E-06	354.7421	-2.2884	0.4295	-5.3280	9.93E-08	1.72E-05
ENSBTAG00000039160	162.5770	3.8234	0.9261	4.1285	3.65E-05	3.79E-04	145.5827	-2.2170	0.5287	-4.1937	2.74E-05	2.11E-03
ENSBTAG00000011011	149.0978	4.6640	0.7564	6.1659	7.01E-10	1.99E-08	133.8720	-2.5962	0.4748	-5.4680	4.55E-08	8.95E-06
ENSBTAG00000005628	262.1524	6.1583	0.7888	7.8075	5.83E-15	3.57E-13	245.6256	-2.1067	0.5119	-4.1156	3.86E-05	2.70E-03
ENSBTAG00000001511	1516.7744	1.9343	0.4747	4.0746	4.61E-05	4.65E-04	1359.1914	-1.7641	0.3578	-4.9309	8.18E-07	1.03E-04
ENSBTAG00000007530	74.5105	6.2911	0.8900	7.0691	1.56E-12	6.87E-11	78.0713	-1.6398	0.4193	-3.9109	9.20E-05	5.39E-03
ENSBTAG00000007596	7645.0201	5.1053	0.3753	13.6039	3.79E-42	2.05E-39	8891.6855	-1.1724	0.2840	-4.1279	3.66E-05	2.59E-03
ENSBTAG00000024928	602.3739	2.9281	0.6016	4.8668	1.13E-06	1.75E-05	544.8249	-2.0197	0.4658	-4.3355	1.45E-05	1.25E-03
ENSBTAG00000011784	698.6093	4.8425	0.6847	7.0724	1.52E-12	6.73E-11	618.6797	-2.6290	0.5054	-5.2021	1.97E-07	3.04E-05
ENSBTAG00000014113	82.9052	6.0174	0.9800	6.1401	8.25E-10	2.30E-08	70.2286	-3.1230	0.5576	-5.6012	2.13E-08	4.60E-06
ENSBTAG00000034366	642.7311	4.8042	0.8060	5.9604	2.52E-09	6.49E-08	599.7981	-2.0078	0.5201	-3.8602	1.13E-04	6.26E-03
ENSBTAG00000019716	2339.4418	10.4515	0.8099	12.9050	4.22E-38	1.72E-35	1971.8177	-4.5815	0.5028	-9.1122	8.07E-20	1.13E-16
ENSBTAG00000011515	313.3737	7.2380	0.7774	9.3102	1.28E-20	1.64E-18	281.5105	-2.6415	0.5058	-5.2224	1.77E-07	2.82E-05
ENSBTAG00000016163	66.1293	6.3396	1.0548	6.0103	1.85E-09	4.93E-08	58.0262	-2.7483	0.5348	-5.1392	2.76E-07	3.99E-05
ENSBTAG00000020764	558.3394	2.4395	0.6001	4.0651	4.80E-05	4.81E-04	504.9261	-1.8689	0.4401	-4.2466	2.17E-05	1.73E-03
ENSBTAG00000020676	27.3285	4.3083	1.1093	3.8836	1.03E-04	9.41E-04	22.7972	-2.3797	0.5725	-4.1568	3.23E-05	2.35E-03
ENSBTAG00000014762	167.8739	5.5106	0.8616	6.3958	1.60E-10	5.22E-09	147.4314	-2.5410	0.5438	-4.6725	2.98E-06	3.34E-04
ENSBTAG00000008636	1416.6220	3.8755	1.2145	3.1911	1.42E-03	8.59E-03	1383.1741	-2.1068	0.4143	-5.0858	3.66E-07	5.14E-05
ENSBTAG00000008631	994.6071	4.8202	0.6748	7.1435	9.10E-13	4.13E-11	946.7466	-1.9092	0.5062	-3.7719	1.62E-04	7.95E-03
ENSBTAG00000023283	112.1392	6.0171	0.9084	6.6239	3.50E-11	1.27E-09	99.6254	-2.4885	0.5386	-4.6201	3.84E-06	4.05E-04
ENSBTAG00000002615	193.2870	3.1639	0.5056	6.2571	3.92E-10	1.18E-08	169.9228	-2.5454	0.4047	-6.2897	3.18E-10	1.02E-07
ENSBTAG00000016424	145.9206	6.7050	0.9567	7.0088	2.40E-12	1.02E-10	124.4214	-3.3761	0.5363	-6.2952	3.07E-10	1.00E-07
ENSBTAG00000021647	38.4542	5.1538	1.1442	4.5045	6.65E-06	8.38E-05	33.8871	-2.1405	0.5680	-3.7681	1.65E-04	8.05E-03
ENSBTAG00000024503	836.2579	3.1378	0.7883	3.9806	6.88E-05	6.62E-04	834.6145	-1.5929	0.4234	-3.7623	1.68E-04	8.18E-03
ENSBTAG00000007705	135.3153	2.6071	0.5731	4.5492	5.39E-06	6.92E-05	135.4888	-1.4649	0.3600	-4.0688	4.72E-05	3.14E-03
ENSBTAG00000046158	894.0882	7.2365	0.6577	11.0028	3.71E-28	9.09E-26	849.2807	-2.0788	0.4992	-4.1645	3.12E-05	2.32E-03
ENSBTAG00000011982	174.5282	6.7023	0.8733	7.6744	1.66E-14	9.41E-13	151.4779	-3.4422	0.4759	-7.2327	4.74E-13	2.66E-10
ENSBTAG00000023338	10841.4875	5.0041	0.2256	22.1815	5.18E-109	6.99E-105	10783.5322	-2.0445	0.2994	-6.8293	8.54E-12	3.52E-09
ENSBTAG00000010785	246.1412	1.6481	0.3398	4.8509	1.23E-06	1.86E-05	213.1850	-1.8573	0.2704	-6.8690	6.47E-12	2.75E-09
ENSBTAG00000047379	104.1963	6.8126	1.0372	6.5682	5.09E-11	1.79E-09	90.9785	-2.6217	0.5529	-4.7420	2.12E-06	2.47E-04
ENSBTAG00000020772	197.5829	3.0209	0.8625	3.5026	4.61E-04	3.40E-03	182.3079	-1.8809	0.4968	-3.7862	1.53E-04	7.64E-03
ENSBTAG00000001009	381.7169	3.4333	0.8153	4.2110	2.54E-05	2.77E-04	331.1292	-2.6969	0.4801	-5.6171	1.94E-08	4.26E-06

ENSBTAG00000006998	142.0297	3.5511	1.0625	3.3422	8.31E-04	5.60E-03	123.6625	-2.1500	0.5622	-3.8243	1.31E-04	6.79E-03
ENSBTAG00000021717	295.7356	7.4516	0.6796	10.9648	5.64E-28	1.36E-25	339.4716	-1.3455	0.2182	-6.1670	6.96E-10	2.12E-07
ENSBTAG00000007071	736.1500	1.8845	0.5498	3.4279	6.08E-04	4.32E-03	659.7378	-1.7344	0.3659	-4.7407	2.13E-06	2.47E-04
ENSBTAG00000021435	1103.6723	4.5949	0.8180	5.6171	1.94E-08	4.22E-07	1119.2098	-1.8584	0.3501	-5.3083	1.11E-07	1.87E-05
ENSBTAG00000003012	833.4196	2.3160	0.4506	5.1401	2.75E-07	4.85E-06	866.9810	-1.2273	0.2667	-4.6024	4.18E-06	4.34E-04
ENSBTAG00000018011	96.1160	3.2113	0.7662	4.1913	2.77E-05	3.00E-04	83.8480	-2.2300	0.5251	-4.2466	2.17E-05	1.73E-03
ENSBTAG00000008573	3183.0775	2.8894	0.5187	5.5699	2.55E-08	5.35E-07	2796.9823	-2.4936	0.3676	-6.7829	1.18E-11	4.72E-09
ENSBTAG00000021523	7193.4909	2.5543	0.3675	6.9505	3.64E-12	1.50E-10	7618.2061	-1.1888	0.3093	-3.8436	1.21E-04	6.60E-03
ENSBTAG00000005413	369.4671	5.7197	0.8321	6.8735	6.26E-12	2.54E-10	328.0298	-2.7482	0.5027	-5.4666	4.59E-08	8.95E-06
ENSBTAG00000008497	52.2648	5.6190	1.1157	5.0363	4.75E-07	7.85E-06	45.0002	-2.3943	0.5693	-4.2059	2.60E-05	2.02E-03
ENSBTAG00000013368	60.0560	4.0366	0.9453	4.2703	1.95E-05	2.19E-04	50.9387	-2.4268	0.5659	-4.2887	1.80E-05	1.52E-03
ENSBTAG00000005018	105.2350	4.9667	0.8369	5.9348	2.94E-09	7.51E-08	93.2783	-2.3839	0.5412	-4.4053	1.06E-05	9.63E-04
ENSBTAG00000014439	59.8535	5.7330	1.1121	5.1552	2.53E-07	4.54E-06	49.8320	-3.0708	0.5709	-5.3786	7.51E-08	1.35E-05
ENSBTAG00000014555	167.1680	7.8684	0.9540	8.2476	1.62E-16	1.22E-14	140.1466	-5.5119	0.4596	-11.9919	3.92E-33	2.75E-29
ENSBTAG00000013496	177.8908	2.8881	0.9020	3.2019	1.37E-03	8.38E-03	149.9198	-2.3224	0.5479	-4.2388	2.25E-05	1.77E-03
ENSBTAG00000008959	115.7302	4.1530	0.6486	6.4032	1.52E-10	4.99E-09	104.5673	-2.4446	0.4633	-5.2767	1.32E-07	2.17E-05
ENSBTAG00000016354	1117.6477	3.1754	0.5543	5.7283	1.01E-08	2.33E-07	1117.1802	-1.6836	0.3256	-5.1706	2.33E-07	3.52E-05
ENSBTAG00000009768	428.0743	6.7269	0.6021	11.1717	5.61E-29	1.40E-26	368.8166	-4.1086	0.3826	-10.7401	6.60E-27	1.85E-23
ENSBTAG00000007883	484.1313	5.5354	0.8188	6.7605	1.37E-11	5.31E-10	439.7353	-2.8784	0.3985	-7.2223	5.11E-13	2.76E-10
ENSBTAG00000010349	704.8808	4.2818	0.5453	7.8520	4.09E-15	2.59E-13	604.2005	-3.5274	0.3899	-9.0472	1.47E-19	1.87E-16
ENSBTAG00000001198	98.1866	6.8707	1.0257	6.6987	2.10E-11	7.86E-10	93.7670	-2.0033	0.5093	-3.9332	8.38E-05	4.97E-03
ENSBTAG00000014560	40.9786	4.1742	0.9150	4.5619	5.07E-06	6.56E-05	36.6389	-2.2691	0.5336	-4.2526	2.11E-05	1.71E-03
ENSBTAG00000024715	121.3413	3.0180	0.7827	3.8559	1.15E-04	1.04E-03	108.0569	-2.0008	0.5210	-3.8404	1.23E-04	6.66E-03
ENSBTAG00000009214	309.4067	3.6443	0.7504	4.8566	1.19E-06	1.82E-05	269.7820	-2.6846	0.4769	-5.6291	1.81E-08	4.04E-06
ENSBTAG00000039731	266.9642	2.7743	0.7717	3.5949	3.25E-04	2.52E-03	225.3526	-2.5131	0.5043	-4.9834	6.25E-07	8.12E-05
ENSBTAG00000015592	818.7520	8.6058	0.9271	9.2821	1.66E-20	2.07E-18	688.0430	-4.5932	0.5146	-8.9263	4.41E-19	5.15E-16
ENSBTAG00000020294	465.7080	2.9639	0.9382	3.1592	1.58E-03	9.40E-03	412.0946	-1.9988	0.5386	-3.7112	2.06E-04	9.62E-03
ENSBTAG00000000816	1468.6712	3.9694	0.3952	10.0441	9.76E-24	1.85E-21	1539.3509	-1.4710	0.3935	-3.7380	1.86E-04	8.89E-03
ENSBTAG00000024272	91.5317	4.7780	0.9511	5.0238	5.07E-07	8.32E-06	78.2784	-2.4205	0.5678	-4.2628	2.02E-05	1.65E-03
ENSBTAG00000019633	113.9207	4.1412	0.6420	6.4504	1.12E-10	3.70E-09	398.7565	1.5897	0.3129	5.0808	3.76E-07	5.22E-05
ENSBTAG00000023372	72.6267	4.1447	0.7948	5.2146	1.84E-07	3.39E-06	241.9832	1.5503	0.2459	6.3048	2.88E-10	9.64E-08
ENSBTAG00000006902	22.4172	4.8543	1.1455	4.2376	2.26E-05	2.49E-04	198.4353	2.5489	0.4701	5.4222	5.89E-08	1.12E-05
ENSBTAG00000038437	75.2286	6.1053	0.9395	6.4987	8.10E-11	2.75E-09	440.1741	1.9070	0.5026	3.7946	1.48E-04	7.44E-03
ENSBTAG00000012931	2250.6450	3.8842	0.3403	11.4129	3.60E-30	9.72E-28	8093.6453	1.6388	0.3323	4.9320	8.14E-07	1.03E-04
ENSBTAG00000021492	85.2751	6.4059	0.8989	7.1261	1.03E-12	4.64E-11	296.9165	1.4732	0.3684	3.9985	6.37E-05	4.00E-03
ENSBTAG00000011460	439.0249	1.9905	0.4255	4.6786	2.89E-06	3.97E-05	1291.0142	1.4569	0.3522	4.1363	3.53E-05	2.54E-03
ENSBTAG00000004617	349.5694	-2.0715	0.4822	-4.2960	1.74E-05	1.98E-04	91.4422	-1.7030	0.4294	-3.9659	7.31E-05	4.50E-03
ENSBTAG00000004954	1615.9688	-2.2934	0.3423	-6.6998	2.09E-11	7.82E-10	1143.3732	1.3262	0.3018	4.3943	1.11E-05	1.00E-03
ENSBTAG00000043985	512.7240	-2.4106	0.4008	-6.0149	1.80E-09	4.80E-08	285.4036	1.0394	0.2525	4.1170	3.84E-05	2.69E-03
ENSBTAG00000006894	3926.8669	-2.1926	0.6900	-3.1779	1.48E-03	8.91E-03	3769.2844	1.8201	0.3596	5.0614	4.16E-07	5.73E-05
ENSBTAG00000018181	3099.0358	-1.7533	0.3017	-5.8112	6.20E-09	1.47E-07	3003.3311	1.4535	0.1886	7.7059	1.30E-14	8.69E-12
ENSBTAG00000009725	795.0144	-5.1560	0.7154	-7.2070	5.72E-13	2.71E-11	281.1559	2.2086	0.5581	3.9576	7.57E-05	4.62E-03
ENSBTAG00000035654	115.0566	-7.6406	0.7980	-9.5744	1.02E-21	1.55E-19	61.2380	2.2632	0.5705	3.9670	7.28E-05	4.50E-03

Supplementary Table 5: KEGG Pathway analysis using DAVID to list 5 pathways that were significantly altered (P<0.05) between Vehicle + GnRH vs Vehicle and PD0325901 +GnRH vs Vehicle + GnRH

Pathway ID	KEGG pathway name	No. of genes	P-value
Vehicle + GnRH vs Vehicle			
04630	JAK-STAT signaling pathway	33	1.3E-4
04668	TNF signaling pathway	35	4.1E-9
04010	MAPK signaling pathway	45	1.3E-3
04370	VEGF signaling pathway	14	59.3E-3
01100	Metabolic pathways	156	2.9E-2
PD0325901 +GnRH vs Vehicle + GnRH			
04668	TNF signaling pathway	17	1.4E-10
04060	Cytokine-cytokine receptor interaction	20	9.8E-8
04062	Chemokine signaling pathway	16	2.0E-6
04010	MAPK signaling pathway	12	9.6E-3
04066	HIF-1 signaling pathway	7	1.1E-2

Supplementary Table 6: Primers used in real-time PCR experiments

Gene*	Forward Primer	Reverse Primer
**Target Genes**		
ADAMTS1	CTGGCAGAAACAGCACAACC	TCCGCCATGCCAAGAGTATC
CYP17A1	CCATCAGAGAAGTGCTCCGAAT	GCCAATGCTGGAGTCAATGA
EGR1	TCCCCTGTTCSCAATGGTTT	TGGGAGAAAAGGTGGTTGTC
FSHR	AGCCCCTTGTCACAACTCTATGTC	GTTCCTCACCGTGAGGTAGATGT
LHR	GCACAGCAAGGAGACCAAATAA	TTGGGTAAGCAGAAACCATAGTCA
PAPPA	CAGAATGCACTGTTACCTGGA	GCTGATCCCAATTCTCTTTCA
SCARB1	ACATCCTGGTCTTGAGTGCG	CCTTGAAGGGGAGCGAGTTT
SLC16A1	TAAACTGCTGTGGTGGCTGGA	ACTTGCCCCCTTTTTCGTCT
STAR	GAGATGGCTGGAAGAAGGTG	GCCAGATAACCCCATCTCAA
STAT3	CTGCAGCAGAAGGTTAGCTACAAA	TTCTAAACAGCTCCACGATTCTCTC
TIMP1	GACATCCGGTTCATCTACACC	ACCAGCAGCATAGGTCTTGG
TNFAIP6	GCTCACGGATGGGGATTCAA	CGTGCTTCCCTGTGGTAGAC
**Reference Genes**		
ACTINB	TGTGGATCAGCAAGCAGGAGTA	TGCGCAAGTTAGGTTTTGTCA
CYCLOPHILIN	GGTCATCGGTCTCTTTGGAA	TCCTTGATCACACGATGGAA
L19	GCCAACTCCCGTCAGCAGA	TGGCTGTACCCTTCCGCTT

**ADAMTS1:* a disintegrin and metalloproteinase with thrombospondin motif 1 ; *CYP17A1*:cytochrome P450 family 17 subfamily A member 1; *EGR1:* early growth response 1; *FSHR:* follicle-stimulating hormone receptor ; *LHR:* luteinizing hormone receptor ; *PAPPA:* pregnancy-associated plasma protein A ; *SCARB1:* scavenger receptor class B member 1; *SLC16A1*: monocarboxylate transporter 1; *STAR*: steroidogenic acute regulatory protein; *STAT3*: signal transducer and activator of transcription 3; *TIMP1:* tissue inhibitor of matrix metalloproteinases 1; *TNFAI*P6:tumor necrosis factor alpha induced protein 6

Supplementary Figure 2: Protein extracts used for immunoblots were obtained from granulosa cells aspirated from an individual ovulating follicle in each cow. Given the limited amount of protein obtained, we cut the membranes to probe for the proteins of different sizes.

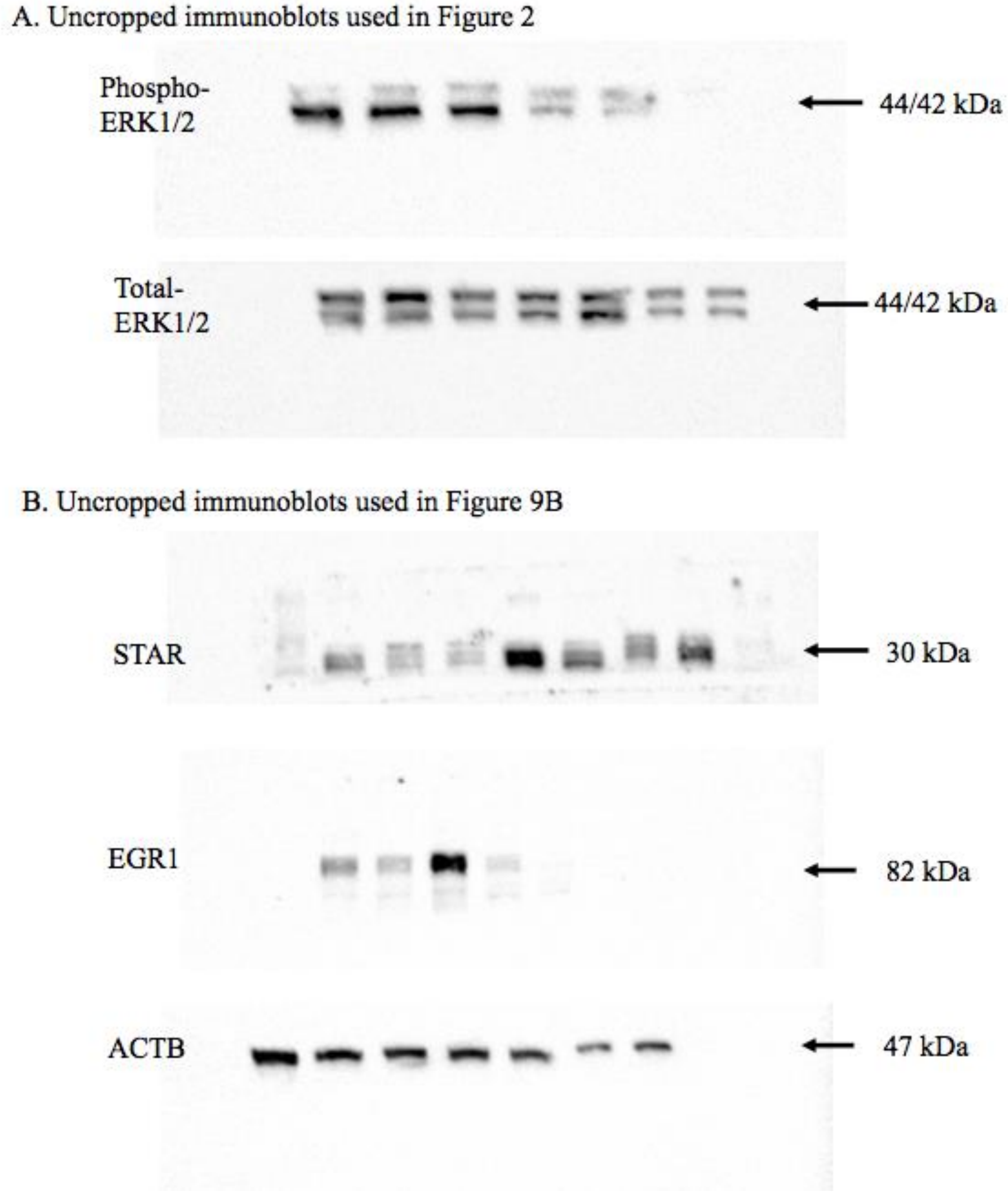

Supplementary Table 7: Antibodies used for immunoblot analyses

Antibody	Concentration	Expected Antibody Size	Manufacturer	References
ACTINB	1:1000	47 kDa	Abcam (ab8227)	1-4
EGR1	1:1000	82 kDa	Santa Cruz (sc-189)	3,4
Phospho-p44/42 MAPK (Phospho-ERK1/2) (Thr202/Tyr204)	1:1000	44 and 42 kDa	Cell Signaling (#4376)	1-4
P44/42 MAPK (Total-ERK1/2)	1:1000	44 and 42 kDa	Cell Signaling (#4695)	1-4
STAR	1:750	30 kDa	Santa Cruz (sc-25806)	3,5,6
Goat pAb to Rb IgG	1:10 000		Abcam (ab6721)	3,4

REFERENCES

1 Ilha, G. F. *et al.* Lack of FSH support enhances LIF–STAT3 signaling in granulosa cells of atretic follicles in cattle. *Reproduction (Cambridge, England)* **150**, 395-403 (2015).

2 Gasperin, B. G. *et al.* Functional status of STAT3 and MAPK3/1 signaling pathways in granulosa cells during bovine follicular deviation. *Theriogenology* **83**, 353-359 (2015).

3 da Rosa, P. *et al.* Mechanistic target of rapamycin is activated in bovine granulosa cells after LH surge but is not essential for ovulation. *Reproduction in domestic animals = Zuchthygiene* **51**, 766-773, doi:10.1111/rda.12745 (2016).

4 da Rosa, P. R. A. *et al.* Reversible meiotic arrest of bovine oocytes by EGFR inhibition and follicular hemisections. *Theriogenology* **99**, 53-62, doi:10.1016/j.theriogenology.2017.05.014 (2017).

5 Woclawek-Potocka, I. *et al.* Effects of lysophopatidic acid on tumor necrosis factor alpha and interferon gamma action in the bovine corpus luteum. *Molecular and cellular endocrinology* **377**, 103-111, doi:10.1016/j.mce.2013.07.005 (2013).

6 Kowalczyk-Zieba, I. *et al.* Lysophosphatidic acid action in the bovine corpus luteum -an in vitro study. *J Reprod Dev* **58**, 661-671 (2012).
